# Supplementary material for: In Vitro Anti-Candida Activity and Action Mode of Benzoxazole Derivatives
Source: Molecules. 2021 Aug 18;26(16):5008. doi: 10.3390/molecules26165008 (PMC8398596; doi:10.3390/molecules26165008)

Supplementary Materials

In vitro anti-*Candida* activity and action mode of benzoxazole derivatives

Monika Staniszewska[1,*], Łukasz Kuryk[2,3], Aleksander Gryciuk[4], Joanna Kawalec[4], Marta Rogalska[4], Joanna Baran[1,4], Edyta Łukowska-Chojnacka[4], Anna Kowalkowska[4,]**

[1,*]Warsaw University of Technology, Centre for Advanced Materials and Technologies CEZAMAT, Poleczki 19, 02-822, Poland, mstaniszewska@pw.edu.pl (MS)

[2] Department of Virology, National Institute of Public Health-National Institute of Hygiene, Chocimska 24, 00-791 Warsaw, Poland

[3]Clinical Science, Targovax Oy, Saukonpaadenranta 2, 00180 Helsinki, Finland

[4*]Warsaw University of Technology, Faculty of Chemistry, Noakowskiego St 3, 00-664 Warsaw, Poland, e-mail: anna.kowalkowska@pw.edu.pl (AK)

Table of Contents

1. Analytical data of compounds **5 – 8** **p. 2-7**

2. ^{1}H and ^{13}C NMR spectra of compounds **5 – 8** **p. 8-28**

3. HR-MS analysis of compounds **5 – 8** **p. 29-39**

4. Antifungal assays **p. 40-44**

1. Analytical data of compounds 5 – 8

2-(1,3-benzoxazol-2-ylsulfanyl)-1-phenylethanone (**5a**) [1–4]: white needles, m.p. 115-116 °C (EtOH) (lit. 124-125 °C [1], 113-115 °C [2]); IR (Nujol, cm^{-1}) ν 1680 (C=O); ^1H NMR (500 MHz, $CDCl_3$) δ = 4.98 (s, 2H, CH_2), 7.24-7.30 (m, 2H, Ar), 7.44-7.46 (m, 1H, Ar), 7.51-7.55 (m, 2H, Ar), 7.58-7.60 (m, 1H, Ar), 7.63-7.66 (m, 1H, Ar), 8.08-8.10 (m, 2H, Ar).

2-(1,3-benzoxazol-2-ylsulfanyl)-1-(4-fluorophenyl)ethanone (**5b**): pale brown crystals, m.p. 114-115 °C (EtOH); IR (Nujol, cm^{-1}) ν 1696 (C=O); ^1H NMR (500 MHz, $CDCl_3$) δ = 4.91 (s, 2H, CH_2), 7.16-7.21 (m, 2H, Ar), 7.23-7.29 (m, 2H, Ar), 7.43-7.45 (m, 1H, Ar), 7.56-7.58 (m, 1H, Ar), 8.09-8.13 (m, 2H, Ar); ^{13}C NMR (125 MHz, $CDCl_3$) δ = 40.83, 110.02, 116.10 (d, J = 22.50 Hz), 118.40, 124.11, 124.39, 131.32 (d, J = 9.78 Hz), 131.63 (d, J = 2.93 Hz), 141.62, 152.04, 163.89, 166.27 (d, J = 257.26 Hz), 190.88; HRMS: calculated for $C_{15}H_{11}FNO_2S$ $[M+H]^+$: 288.04890. Found: 288.04861, mass error -0.00029, ca 1.0 ppm.

2-(1,3-benzoxazol-2-ylsulfanyl)-1-(4-chlorophenyl)ethanone (**5c**): white needles, m.p. 121-122 °C (EtOH) (lit. 129-130 °C [1]); IR (Nujol, cm^{-1}) ν 1709 (C=O); ^1H NMR (500 MHz, $CDCl_3$) δ = 4.89 (s, 2H, CH_2), 7.22-7.28 (m, 2H, Ar), 7.42-7.43 (m, 1H, Ar), 7.47-7.49 (m, 2H, Ar), 7.54-7.56 (m, 1H, Ar), 7.99-8.02 (m, 2H, Ar); ^{13}C NMR (125 MHz, $CDCl_3$) δ = 40.77, 110.03, 118.42, 124.11, 124.39, 129.23, 129.95, 133.51, 140.63, 141.66, 152.06, 163.77, 191.32; HRMS: calculated for $C_{15}H_{11}ClNO_2S$ $[M+H]^+$: 304.01935. Found: 304.01912, mass error -0.00023, ca 0.76 ppm.

2-(1,3-benzoxazol-2-ylsulfanyl)-1-(4-bromophenyl)ethanone (**5d**): white crystals, m.p. 137-138 °C (EtOH) (lit. 138-139 °C [1], 129-131 °C [4]); IR (Nujol, cm^{-1}) ν 1689 (C=O); ^1H NMR (500 MHz, $CDCl_3$) δ = 4.88 (s, 2H, CH_2), 7.22-7.28 (m, 2H, Ar), 7.42-7.43 (m, 1H, Ar), 7.54-7.56 (m, 1H, Ar), 7.64-7.66 (m, 2H, Ar), 7.92-7.93 (m, 2H, Ar); ^{13}C NMR (125 MHz, $CDCl_3$) δ = 40.72, 110.03, 118.43, 124.12, 124.39, 129.40, 130.01, 132.23, 133.91, 141.66, 152.06, 163.74, 191.53; HRMS: calculated for $C_{15}H_{11}BrNO_2S$ $[M+H]^+$: 347.96884. Found: 347.96878, mass error -0.00006, ca 0.2 ppm.

2-(1,3-benzoxazol-2-ylsulfanyl)-1-(2,4-difluorophenyl)ethanone (**5e**): white crystals, m.p. 93-94 °C (EtOH); IR (Nujol, cm^{-1}) ν 1678 (C=O); ^1H NMR (500 MHz, $CDCl_3$) δ = 4.82 (d, J = 3.18 Hz, 2H, CH_2), 6.94-6.99 (m, 1H, Ar), 7.01-7.04 (m, 1H, Ar), 7.23-7.29 (m, 2H, Ar), 7.43-7.45 (m, 1H, Ar), 7.55-7.57 (m, 1H, Ar), 8.00-8.05 (m, 1H, Ar); ^{13}C NMR (125 MHz, $CDCl_3$) δ = 43.94 (d, J = 9.78 Hz), 104.90 (dd, J = 27.39 Hz, J = 25,43 Hz), 109.95, 112.72 (dd, J = 21.52 Hz, J = 3.91 Hz), 118.54, 120.47 (dd, J = 12.72 Hz, J = 3.91 Hz), 124.02, 124.31, 133.23 (dd, J = 10.76 Hz, J = 3.91 Hz), 141.76, 152.02, 162.86 (dd, J = 258.23 Hz, J = 12.72 Hz), 163.49, 166.41 (dd, J = 259.21 Hz, J = 12.72

Hz), 188.90 (d, J = 4.89 Hz); HRMS: calculated for $C_{15}H_{10}F_2NO_2S$ $[M+H]^+$: 306.03948. Found: 306.03953, mass error 0.00005, ca 0.2 ppm.

2-(1,3-benzoxazol-2-ylsulfanyl)-1-(2,4-dichlorophenyl)ethanone (**5f**): pale brown crystals, m.p. 86-87 °C (EtOH); IR (Nujol, cm^{-1}) ν 1684 (C=O); ^1H NMR (500 MHz, $CDCl_3$) δ = 4.74 (s, 2H, CH_2), 7.24-7.30 (m, 2H, Ar), 7.35 (dd, J = 8.31 Hz, J = 1.96 Hz, 1H, Ar), 7.42-7.44 (m, 2H, Ar), 7.49 (d, J = 1.96 Hz, 1H, Ar), 7.54-7.56 (m, 1H, Ar), 7.65 (d, J = 8.31 Hz, 1H, Ar); ^{13}C NMR (125 MHz, $CDCl_3$) δ = 42.64, 110.01, 118.49, 124.15, 124.40, 127.53, 130.37, 131.31, 132.35, 135.52, 138.38, 141.61, 152.11, 162.28, 194.30; HRMS: calculated for $C_{15}H_{10}Cl_2NO_2S$ $[M+H]^+$: 337.98038. Found: 337.98019, mass error -0.00019, ca 0.6 ppm.

2-(1,3-benzoxazol-2-ylsulfanyl)-1-(2,5-dichlorophenyl)ethanone (**5g**): yellow crystals, m.p. 88-89 °C (EtOH); IR (Nujol, cm^{-1}) ν 1690 (C=O); ^1H NMR (500 MHz, $CDCl_3$) δ = 4.71 (s, 2H, CH_2), 7.24-7.31 (m, 2H, Ar), 7.38-7.45 (m, 3H, Ar), 7.56-7.58 (m, 1H, Ar), 7.67-7.68 (m, 1H, Ar); ^{13}C NMR (125 MHz, $CDCl_3$) δ = 42.44, 110.02, 118.51, 124.19, 124.46, 129.33, 130.02, 131.57, 132.37, 133.31, 138.71, 141.55, 152.16, 163.22, 194.31; HRMS: calculated for $C_{15}H_{10}Cl_2NO_2S$ $[M+H]^+$: 337.98038. Found: 337.98088, mass error 0.0005, ca 1.5 ppm.

2-(1,3-benzoxazol-2-ylsulfanyl)-1-(3,4-dichlorophenyl)ethanone (**5h**): brown crystals, m.p. 129-130 °C (EtOH); IR (Nujol, cm^{-1}) ν 1704 (C=O); ^1H NMR (500 MHz, $CDCl_3$) δ = 4.86 (s, 2H, CH_2), 7.24-7.31 (m, 2H, Ar), 7.44-7.46 (m, 1H, Ar), 7.57-7.59 (m, 1H, Ar), 7.61 (d, J = 8.31 Hz, 1H, Ar), 7.92 (dd, J = 8.31 Hz, J = 1.96 Hz, 1H, Ar), 8.19 (d, J = 1.96 Hz); ^{13}C NMR (125 MHz, $CDCl_3$) δ = 40.40, 110.04, 118.43, 124.18, 124.43, 127.51, 130.53, 130.99, 133.66, 134.70, 138.74, 141.56, 152.08, 163.47, 190.50; HRMS: calculated for $C_{15}H_{10}Cl_2NO_2S$ $[M+H]^+$: 337.98038. Found: 337.98065, mass error 0.00027, ca 0.8 ppm.

2-(1,3-benzoxazol-2-ylsulfanyl)-1-(2,3,4-trichlorophenyl)ethanone (**5i**): pale beige crystals, m.p. 106-107 °C (EtOH); IR (Nujol, cm^{-1}) ν 1720 (C=O); ^1H NMR (500 MHz, $CDCl_3$) δ = 4.66 (s, 2H, CH_2), 7.24-7.30 (m, 2H, Ar), 7.42-7.44 (m, 2H, Ar), 7.46 (s, 2H, Ar), 7.53-7.55 (m, 2H, Ar); ^{13}C NMR (125 MHz, $CDCl_3$) δ = 42.19, 100.04, 118.47, 124.22, 124.45, 127.62, 128.75, 130.99, 132.90, 137.19, 137.98, 141.51, 152.14, 163.07, 194.54; HRMS: calculated for $C_{15}H_9Cl_3NO_2S$ $[M+H]^+$: 371.94141. Found: 371.94162, mass error 0.00021, ca 0.6 ppm.

2-(1,3-benzoxazol-2-ylsulfanyl)-1-(2,4,5-trichlorophenyl)ethanone (**5j**): beige crystals, m.p. 96-97 °C (EtOH); IR (Nujol, cm^{-1}) ν 1713 (C=O); ^1H NMR (500 MHz, $CDCl_3$) δ = 4.68 (s, 2H, CH_2), 7.24-7.31 (m, 2H, Ar), 7.43-7.45 (m, 2H, Ar), 7.55-7.57 (m, 2H, Ar), 7.58 (s, 1H, Ar), 7.84

(s, 1H, Ar); ^{13}C NMR (125 MHz, $CDCl_3$) δ = 42.29, 110.05, 118.50, 124.24, 124.49, 129.94, 131.88, 131.80, 131.99, 136.57, 136.71, 141.51, 152.18, 163.11, 193.33; HRMS: calculated for $C_{15}H_9Cl_3NO_2S$ $[M+H]^+$: 371.94141. Found: 371.94167, mass error 0.00026, ca 0.7 ppm.

2-(1,3-benzoxazol-2-ylsulfanyl)-1-(2,4,6-trichlorophenyl)ethanone (**5k**): white crystals, m.p. 140-141 °C (EtOH); IR (Nujol, cm^{-1}) ν 1724 (C=O); ^1H NMR (500 MHz, $CDCl_3$) δ = 4.71 (s, 2H, CH_2), 7.25-7.31 (m, 2H, Ar), 7.36 (s, 2H, Ar), 7.43-7.45 (m, 2H, Ar), 7.56-7.58 (m, 2H, Ar); ^{13}C NMR (125 MHz, $CDCl_3$) δ = 43.11, 109.99, 118.59, 124.22, 124.47, 128.35, 128.39, 131.88, 135.84, 136.67, 141.62, 152.03, 162.61, 193.67; HRMS: calculated for $C_{15}H_9Cl_3NO_2S$ $[M+H]^+$: 371.94141. Found: 371.94171, mass error 0.0003, ca 0.8 ppm.

2-[(5-bromo-1,3-benzoxazol-2-yl)sulfanyl]-1-phenylethanone (**6a**): white solid, m.p. 125-126 °C; IR (Nujol, cm^{-1}) ν 1692 (C=O); ^1H NMR (500 MHz, $CDCl_3$) δ = 4.95 (s, 2H, CH_2), 7.31 (d, J = 8.56 Hz, 1H, Ar), 7.35-7.37 (m, 1H, Ar), 7.52-7.55 (m, 2H, Ar), 7.64-7.67 (m, 1H, Ar), 7.70 (d, J = 1.96 Hz, 1H, Ar), 8.06-8.09 (m, 2H, Ar); ^{13}C NMR (125 MHz, $CDCl_3$) δ = 41.17, 111.11, 117.17, 121.46, 126.93, 128.52, 128.92, 134.12, 135.10, 143.27, 151.03, 165.66, 192.09; HRMS: calculated for $C_{15}H_{11}BrNO_2S$ $[M+H]^+$: 347.96884. Found: 347.96895, mass error 0.00111, ca 0.3 ppm.

2-[(5-bromo-1,3-benzoxazol-2-yl)sulfanyl]-1-(4-fluorophenyl)ethanone (**6b**): white needles, m.p. 107-108 °C (EtOH); IR (Nujol, cm^{-1}) ν 1676 (C=O); ^1H NMR (500 MHz, $CDCl_3$) δ = 4.90 (s, 2H, CH_2), 7.18-7.23 (m, 2H, Ar), 7.31 (d, J = 8.56 Hz, 1H, Ar), 7.37 (dd, J = 8.56 Hz, J = 1.71 Hz, 1H, Ar), 7.70 (d, J = 1.71 Hz, 1H, Ar), 8.09-8.13 (m, 2H, Ar); ^{13}C NMR (125 MHz, $CDCl_3$) δ = 40.90, 111.14, 116.16 (d, J = 21.52 Hz), 117.21, 121.46, 127.00, 131.30 (d, J = 9.78 Hz), 131.58 (d, J = 2.93 Hz), 143.20, 151.05, 165.29, 166.44 (d, J = 225.95 Hz), 190.61; HRMS: calculated for $C_{15}H_{10}BrFNO_2S$ $[M+H]^+$: 365.95942. Found: 365.95979, mass error 0.00037, ca 1 ppm.

2-[(5,7-dibromo-1,3-benzoxazol-2-yl)sulfanyl]-1-phenylethanone (**7a**): white solid, m.p. 159-160 °C (decomp.); IR (Nujol, cm^{-1}) ν 1678 (C=O); ^1H NMR (500 MHz, $CDCl_3$) δ = 4.94 (s, 2H, CH_2), 7.52-7.55 (m, 3H, Ar), 7.62 (d, J = 1.71 Hz, 1H, Ar), 7.64-7.67 (m, 1H, Ar), 8.06-8.08 (m, 2H, Ar); ^{13}C NMR (125 MHz, $CDCl_3$) δ = 41.26, 102.45, 117.46, 120.54, 128.53, 128.96, 129.49, 134.18, 135.07, 143.42, 149.51, 166.23, 191.83; HRMS: calculated for $C_{15}H_{10}Br_2NO_2S$ $[M+H]^+$: 427.87730. Found: 427.87776, mass error 0.00046, ca 1.1 ppm.

2-[(5,7-dibromo-1,3-benzoxazol-2-yl)sulfanyl]-1-(4-fluorophenyl)ethanone (**7b**): white solid, m.p. 164-165 °C; IR (Nujol, cm^{-1}) ν 1672 (C=O); ^1H NMR (500 MHz, $CDCl_3$) δ = 4.89 (s, 2H, CH_2), 7.17-7.22 (m, 2H, Ar), 7.42-7.44 (m, 2H, Ar), 8.07-8.11 (m, 2H, Ar); ^{13}C NMR (125 MHz, $CDCl_3$)

δ = 41.00, 102.41, 126.18 (d, J = 21.52 Hz), 120.47, 130.03, 131.28 (d, J = 9.78 Hz), 131.51 (d, J = 2.93 Hz), 133.06, 142.99, 149.90, 165.87, 166.32 (d, J = 257.25 Hz), 190.31; HRMS: calculated for $C_{15}H_9Br_2FNO_2S$ [M+H]$^+$: 445.86788. Found: 445.86772, mass error -0.00016, ca 0.4 ppm.

2-(1,3-benzoxazol-2-ylsulfanyl)-1-(4-fluorophenyl)ethanol (**8b**): pale beige crystals, m.p.

84-85 °C (hexane/EtOAc 10/1); IR (Nujol, cm^{-1}) ν 3303 (C–O), 1136 (C–O); ^{1}H NMR (500 MHz, $CDCl_3$) δ = 3.50 (dd, J = 14.43 Hz, J = 8.07 Hz, 1H, C*H*HS), 3.66 (dd, J = 14.43 Hz, J = 3.42 Hz, 1H, CH*H*S), 4.41 (d, J = 3.42 Hz, OH), 5.19-5.21 (m, 1H, C*H*OH), 7.05-7.10 (m, 2H, Ar), 7.26-7.33 (m, 2H, Ar), 7.43-7.47 (m, 3H, Ar), 7.60-7.62 (m, 1H, Ar); ^{13}C NMR (125 MHz, $CDCl_3$) δ = 41.18, 73.05, 110.03, 115.41 (d, J = 21.52 Hz), 118.34, 124.25, 124.54, 127.53 (d, J = 7.83 Hz), 138.18 (d, J = 2.93), 141.19, 152.03, 162.42 (d, J = 246.50 Hz), 165.61; HRMS: calculated for $C_{15}H_{13}FNO_2S$ [M+H]$^+$: 290.06455. Found: 290.06424, mass error -0,00031, ca 1.1 ppm.

2-(1,3-benzoxazol-2-ylsulfanyl)-1-(4-chlorophenyl)ethanol (**8c**): pale beige crystals, m.p. 78-79 °C (hexane/EtOAc 10/1); IR (Nujol, cm^{-1}) ν 3345 (C–O), 1140 (C–O); ^{1}H NMR (500 MHz, $CDCl_3$) δ = 3.48 (dd, J = 14.43 Hz, J = 8.07 Hz, 1H, C*H*HS), 3.66 (dd, J = 14.43 Hz, J = 3.18 Hz, 1H, CH*H*S), 5.20 (dd, J = 8.07 Hz, J = 3.18 Hz, 1H, C*H*OH), 7.26-7.33 (m, 2H, Ar), 7.34-7.37 (m, 2H, Ar), 7.40-7.43 (m, 2H, Ar), 7.45-7.47 (m, 1H, Ar), 7.59-7.61 (m, 1H, Ar); ^{13}C NMR (125 MHz, $CDCl_3$) δ = 41.07, 72.98, 110.04, 118.32, 124.28, 124.57, 127.23, 128.68, 133.67, 140.88, 141.09, 152.01, 165.61; HRMS: calculated for $C_{15}H_{13}ClNO_2S$ [M+H]$^+$: 306.03500. Found: 306.03513, mass error 0.00013, ca 0.4 ppm.

2-(1,3-benzoxazol-2-ylsulfanyl)-1-(4-bromophenyl)ethanol (**8d**): pale beige crystals, m.p. 92-93 °C (hexane/EtOAc 10/1); IR (Nujol, cm^{-1}) ν 3235 (C–O), 1140 (C–O); ^{1}H NMR (500 MHz, $CDCl_3$) δ = 3.48 (dd, J = 14.43 Hz, J = 8.07 Hz, 1H, C*H*HS), 3.66 (dd, J = 14.43 Hz, J = 3.18 Hz, 1H, CH*H*S), 4.54 (br s, 1H, OH), 5.19 (d, 1H, J = 8.07 Hz, J = 3.18 Hz, C*H*OH), 7.26-7.33 (m, 2H, Ar), 7.34-7.37 (m, 2H, Ar), 7.45-7.47 (m, 1H, Ar), 7.50-7.53 (m, 2H, Ar), 7.60-7.62 (m, 1H, Ar); ^{13}C NMR (125 MHz, $CDCl_3$) δ = 41.02, 73.10, 110.06, 118.35, 121.82, 124.29, 124.58, 127.58, 131.63, 141.12, 141.43, 152.04, 165.61; HRMS: calculated for $C_{15}H_{13}BrNO_2S$ [M+H]$^+$: 349.98449. Found: 349.98453, mass error 0.00004, ca 0.1 ppm.

2-(1,3-benzoxazol-2-ylsulfanyl)-1-(2,4-difluorophenyl)ethanol (**8e**): pale rose crystals, m.p. 48-49 °C; IR (Nujol, cm^{-1}) ν 3573 (C–O), 1132 (C–O); ^{1}H NMR (500 MHz, $CDCl_3$) δ = 3.60 (ddd, J = 14.67 Hz, J = 7.34 Hz, J = 0.49 Hz, 1H, C*H*HS), 3.72 (dd, J = 14.67 Hz, J = 3.18 Hz, 1H, CH*H*S), 5.25 (d, J = 4.16 Hz, 1H, OH), 5.47-5.51 (m, 1H, C*H*OH), 6.79-6.83 (m, 1H, Ar), 6.89-6.93 (m, 1H, Ar), 7.27-7.30 (m, 1H, Ar), 7.31-7.34 (m, 1H, Ar), 7.45-7.47 (m, 1H, Ar), 7.60-7.65

(m, 2H, Ar); ^{13}C NMR (125 MHz, $CDCl_3$) δ = 39.69, 68.22, 103.65 (t, J = 25.43 Hz), 110.09, 111.33 (dd, J = 21.52 Hz, J = 3.91 Hz), 118.30, 124.37, 124.64, 125.45 (dd, J = 13.69 Hz, J = 3.91 Hz), 128.74 (dd, J = 9.78 Hz, d = 5.87 Hz), 140.88, 152.08, 159.43 (dd, J = 248.45 Hz, 11.74 Hz), 162.51 (dd, J = 248.50 Hz, J = 12.72 Hz), 166.16; HRMS: calculated for $C_{15}H_{12}F_2NO_2S$ $[M+H]^+$: 308.05513. Found: 308.05496, mass error -0.00017, ca 0.6 ppm.

2-(1,3-benzoxazol-2-ylsulfanyl)-1-(2,4-dichlorophenyl)ethanol (**8f**): white crystals, m.p. 93-94 °C (hexane/EtOAc 10/1); IR (Nujol, cm^{-1}) ν 3443 (C–O), 1138 (C–O); ^{1}H NMR (500 MHz, $CDCl_3$) δ = 3.58 (dd, J = 14.92 Hz, J = 6.85 Hz, 1H, C*H*HS), 3.72 (dd, J = 14.92 Hz, J = 2.69 Hz, 1H, CH*H*S), 5.53-5.56 (m, 1H, C*H*OH), 5.65 (d, J = 3.91 Hz, 1H, OH), 7.27-7.35 (m, 3H, Ar), 7.38 (d, J = 1.96 Hz, 1H, Ar), 7.45-7.47 (m, 1H, Ar), 7.60-7.62 (m, 1H, Ar), 7.68-7.70 (m, 1H, Ar); ^{13}C NMR (125 MHz, $CDCl_3$) δ = 39.03, 70.84, 110.13, 118.28, 124.44, 124.69, 127.35, 128.82, 129.12, 132.05, 134.04, 138.44, 140.78, 152.10, 166.37; HRMS: calculated for $C_{15}H_{12}Cl_2NO_2S$ $[M+H]^+$: 339.99603. Found: 339.99597, mass error -0.00006, ca 0.2 ppm.

2-(1,3-benzoxazol-2-ylsulfanyl)-1-(3,4-dichlorophenyl)ethanol (**8h**): white needles, m.p. 76-77 °C (hexane/EtOAc 10/1); IR (Nujol, cm^{-1}) ν 3326 (C–O), 1140 (C–O); ^{1}H NMR (500 MHz, $CDCl_3$) δ = 3.46 (dd, J = 14.67 Hz, J = 7.83 Hz, 1H, C*H*HS), 3.66 (dd, J = 14.67 Hz, J = 3.18 Hz, 1H, CH*H*S), 4.80 (br s, 1H, OH), 5.17-5.22 (m, 1H, C*H*OH), 7.27-7.34 (m, 3H, Ar), 7.45 (d, J = 8.31 Hz, 1H, Ar), 7.45-7.47 (m, 1H, Ar), 7.70-7.62 (m, 2H, Ar); ^{13}C NMR (125 MHz, $CDCl_3$) δ = 40.96, 72.67, 110.10, 118.35, 124.40, 124.65, 125.22, 127.96, 130.48, 131.84, 132.71, 140.99, 142.70, 152.07, 165.56; HRMS: calculated for $C_{15}H_{12}Cl_2NO_2S$ $[M+H]^+$: 339.99603. Found: 339.99596, mass error -0.00007, ca 0.2 ppm.

2-(1,3-benzoxazol-2-ylsulfanyl)-1-(2,3,4-trichlorophenyl)ethanol (**8i**): beige crystals, m.p. 119-120 °C (hexane/EtOAc 10/1); IR (Nujol, cm^{-1}) ν 3265 (C–O), 1140 (C–O); ^{1}H NMR (500 MHz, $CDCl_3$) δ = 3.59 (dd, J = 14.92 Hz, J = 6.60 Hz, 1H, C*H*HS), 3.73 (dd, J = 14.92 Hz, J = 2.45 Hz, 1H, CH*H*S), 5.55-5.58 (m, 1H, C*H*OH), 5.87 (d, J = 3.91 Hz, OH), 7.28-7.35 (m, 2H, Ar), 7.44 (d, J = 8.56 Hz, 1H, Ar), 7.46-7.48 (m, 1H, Ar), 7.60-7.62 (m, 1H, Ar), 7.64 (dd, J = 8.56 Hz, J = 0.49 Hz, 1H, Ar); ^{13}C NMR (125 MHz, $CDCl_3$) δ = 38.79, 71.68, 110.16, 118.28, 124.54, 124.75, 126.19, 128.40, 131.17, 131.68, 133.31, 140.57, 140.68, 152.13, 166.36; HRMS: calculated for $C_{15}H_{11}Cl_3NO_2S$ $[M+H]^+$: 373.95706. Found: 373.95718, mass error 0.00012, ca 0.3 ppm.

2-(1,3-benzoxazol-2-ylsulfanyl)-1-(2,4,5-trichlorophenyl)ethanol (**8j**): pale yellow crystals, m.p. 123-124 °C (hexane/EtOAc 10/1); IR (Nujol, cm^{-1}) ν 3240 (C–O), 1140 (C–O); ^{1}H NMR (500 MHz, $CDCl_3$) δ = δ = 3.57 (dd, J = 14.92 Hz, J = 6.85 Hz, 1H, C*H*HS), 3.69 (dd, J = 14.92 Hz, J = 2.69 Hz, 1H, CH*H*S), 5.51 (dd, J = 6.85 Hz, J = 2.20 Hz, 1H, C*H*OH), 5.92 (br s, 1H, OH),

7.29-7.39 (m, 2H, Ar), 7.47-7.48 (m, 1H, Ar), 7.47 (s, 1H, Ar), 7.61-7.63 (m, 1H, Ar), 7.87 (s, 1H, Ar); ^{13}C NMR (125 MHz, $CDCl_3$) δ = 38.86, 70.95, 110.19, 118.31, 124.57, 124.79, 129.52, 129.87, 130.64, 131.76, 132.36, 140.04, 140.64, 152.15, 166.38; HRMS: calculated for $C_{15}H_{11}Cl_3NO_2S$ $[M+H]^+$: 373.95706. Found: 373.95711, mass error 0.00005, ca 0.1 ppm.

References

1. Loghmani-Khouzani, H.; Hajiheidari, D. Synthesis of difluorinated β-ketosulfones and novel gem-difluoromethylsulfone-containing heterocycles as fluorinated building blocks. *J. Fluor. Chem.* **2010**, *131*, 561–569, doi:10.1016/j.jfluchem.2009.12.022.

2. Varun, B.V.; Gadde, K.; Prabhu, K.R. Synthesis of *α*-sulfenyl monoketones: Via a metal-free oxidative cross dehydrogenative coupling (CDC) reaction. *Org. Biomol. Chem.* **2016**, *14*, 7665–7670, doi:10.1039/c6ob01243d.

3. Yang, Z.; Li, J.; Hua, J.; Yang, T.; Yi, J.; Zhou, C. KI/K 2 S 2 O 8 -Mediated *α*-C-H Sulfenylation of Carbonyl Compounds with (Hetero)Aryl Thiols. *Synlett* **2017**, *28*, 2325–2329, doi:10.1055/s-0036-1588483.

4. Md. Khaja Mohinuddin, P.; Gangi Reddy, N.C. Zinc oxide catalyzed solvent-free mechanochemical route for C-S bond construction: A sustainable process. *European J. Org. Chem.* **2017**, *2017*, 1207–1214, doi:10.1002/ejoc.201601425.

2. ^{1}H and ^{13}C NMR spectra of compounds 5 – 8

2-(1,3-benzoxazol-2-ylsulfanyl)-1-phenylethanone (**5a**)

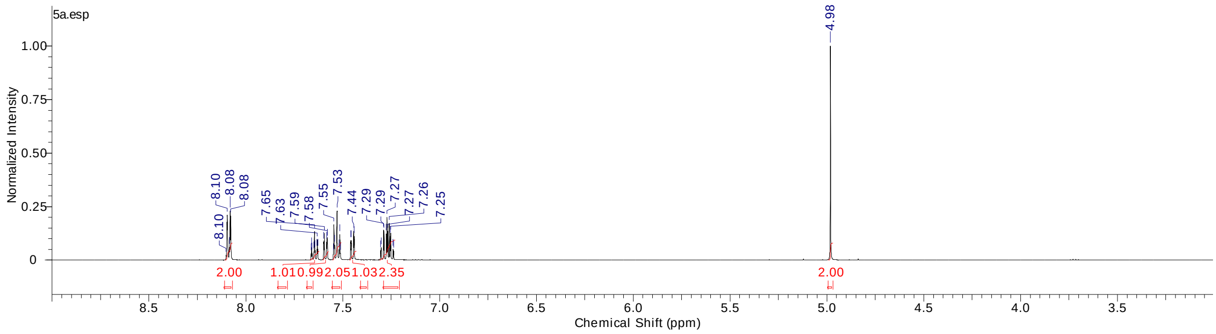

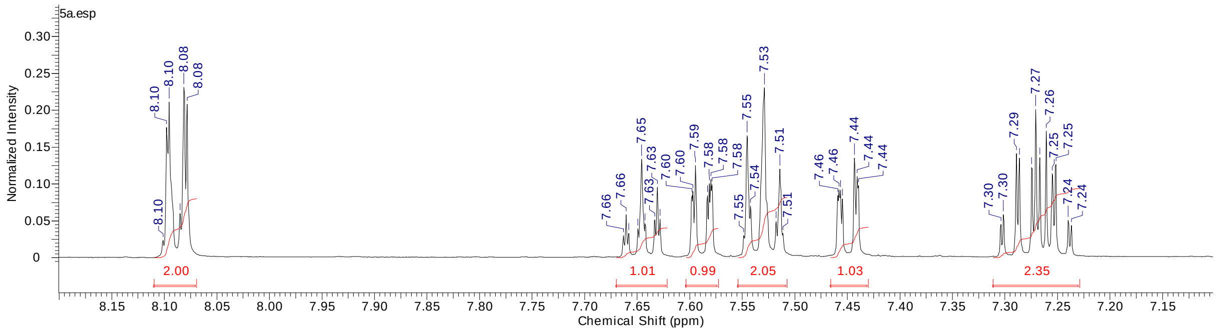

2-(1,3-benzoxazol-2-ylsulfanyl)-1-(4-fluorophenyl)ethanone (**5b**)

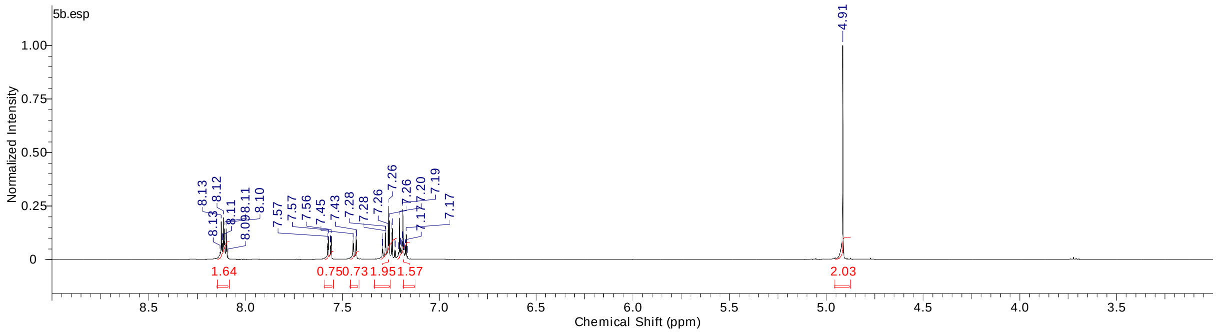

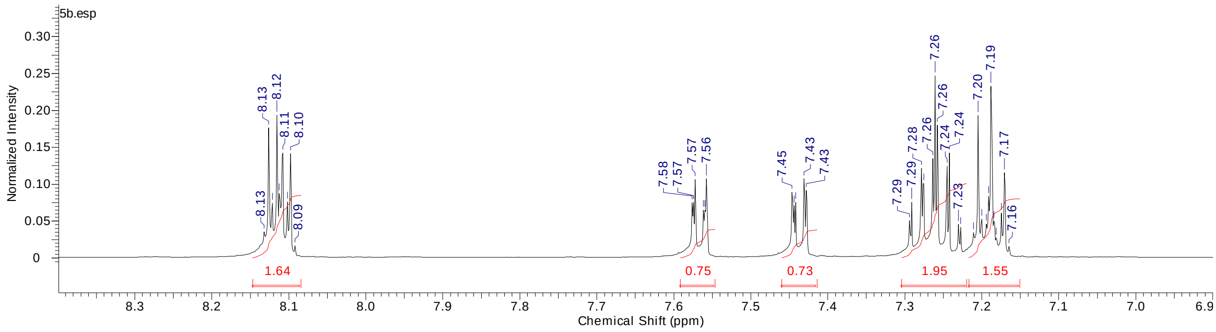

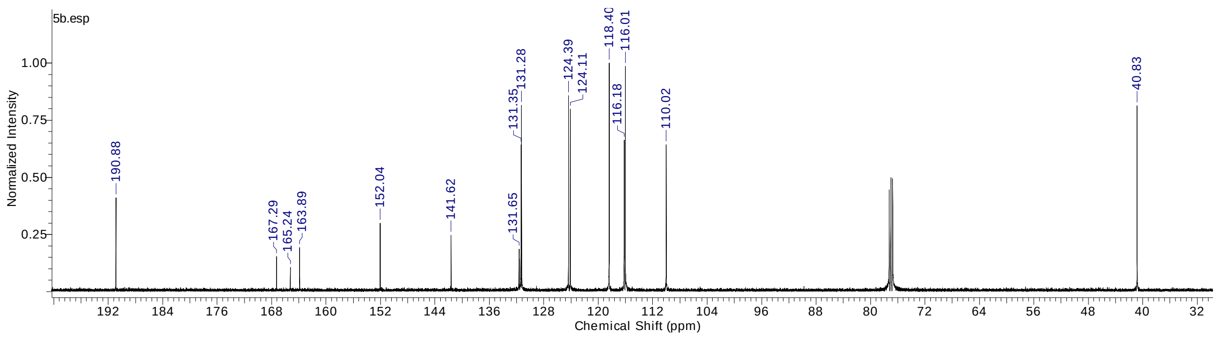

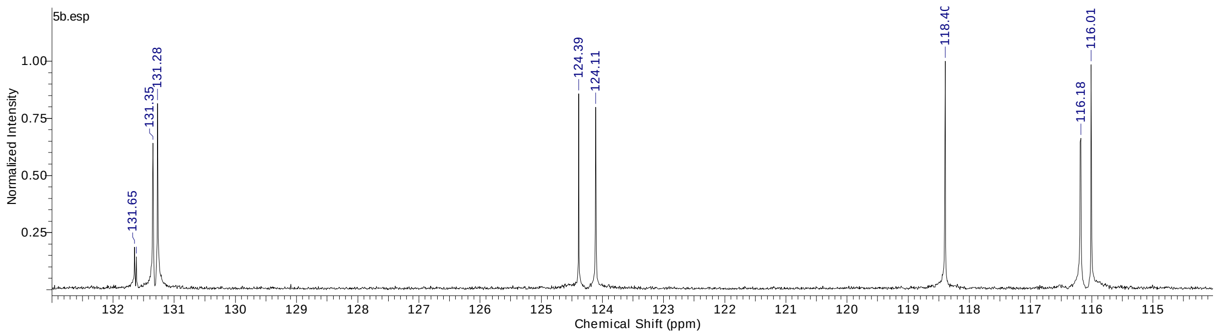

2-(1,3-benzoxazol-2-ylsulfanyl)-1-(4-chlorophenyl)ethanone (**5c**)

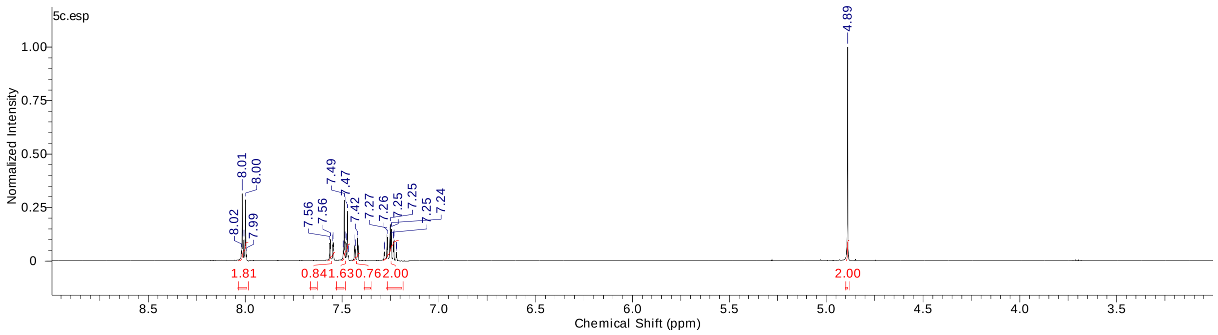

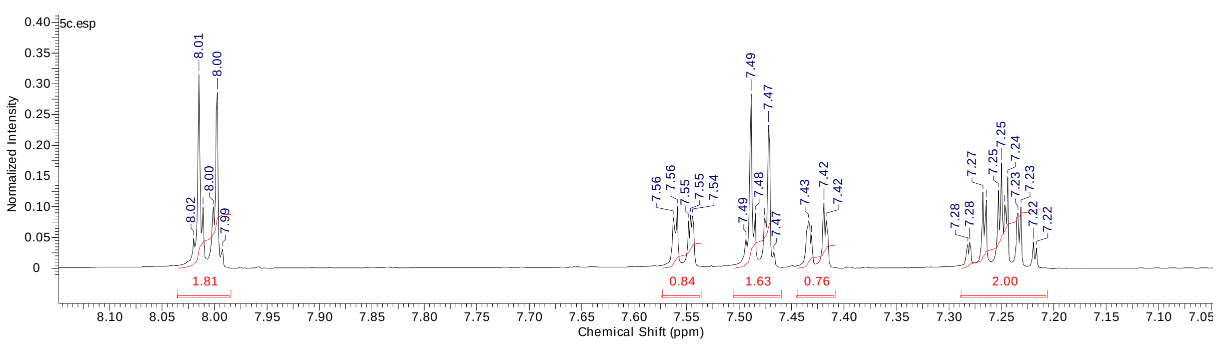

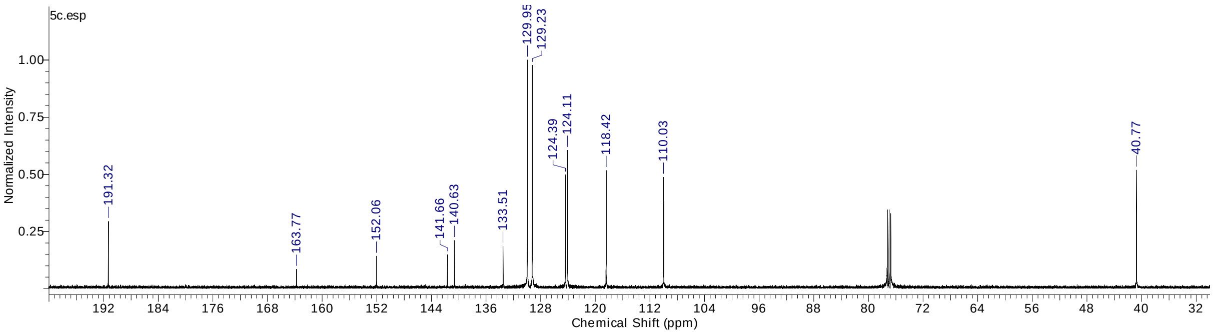

2-(1,3-benzoxazol-2-ylsulfanyl)-1-(4-bromophenyl)ethanone (**5d**)

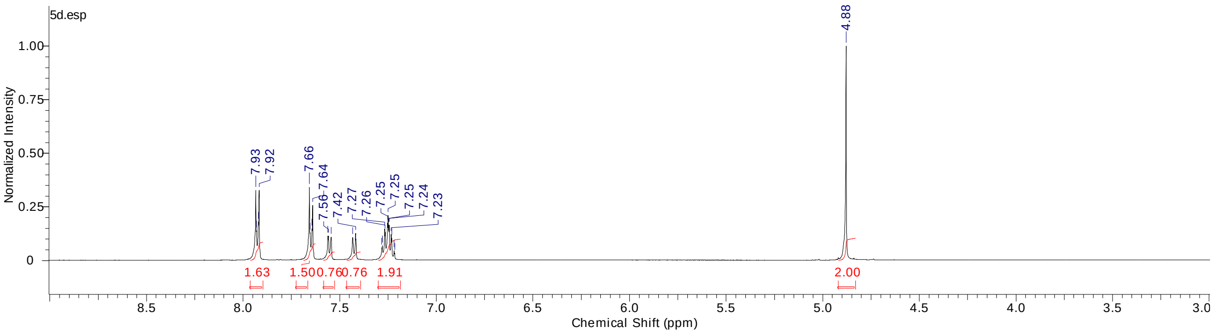

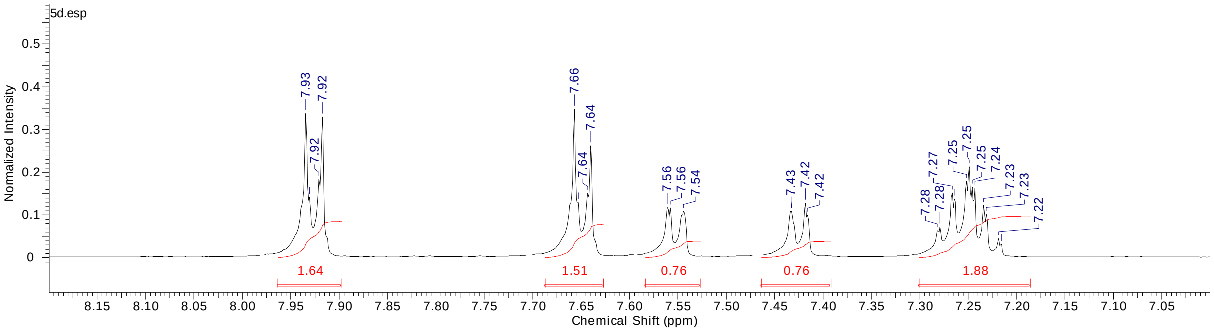

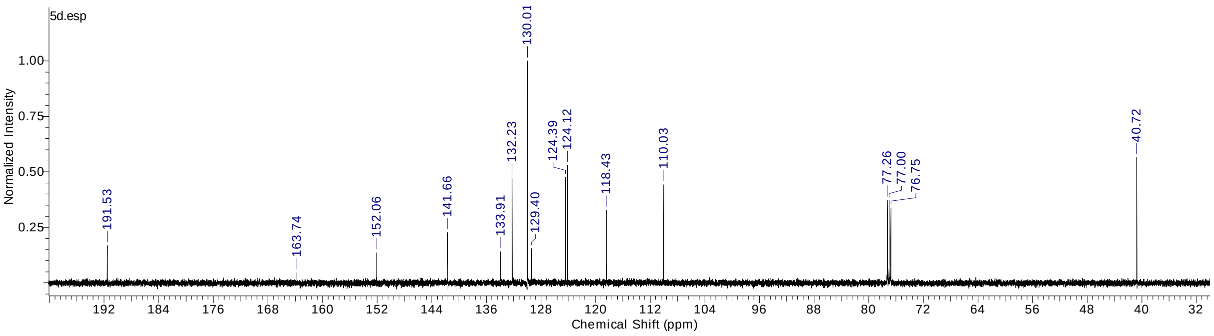

2-(1,3-benzoxazol-2-ylsulfanyl)-1-(2,4-difluorophenyl)ethanone (**5e**)

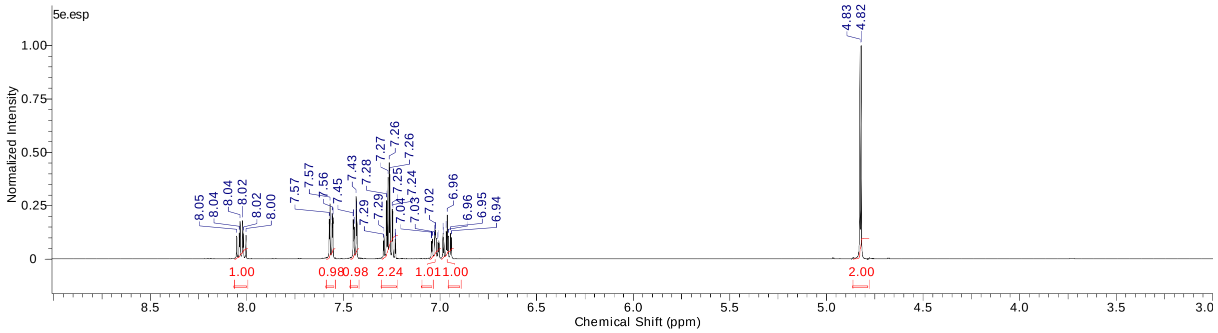

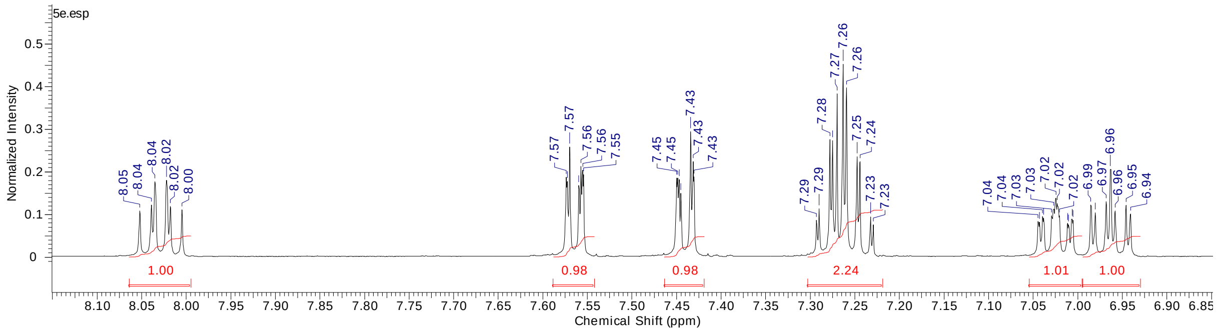

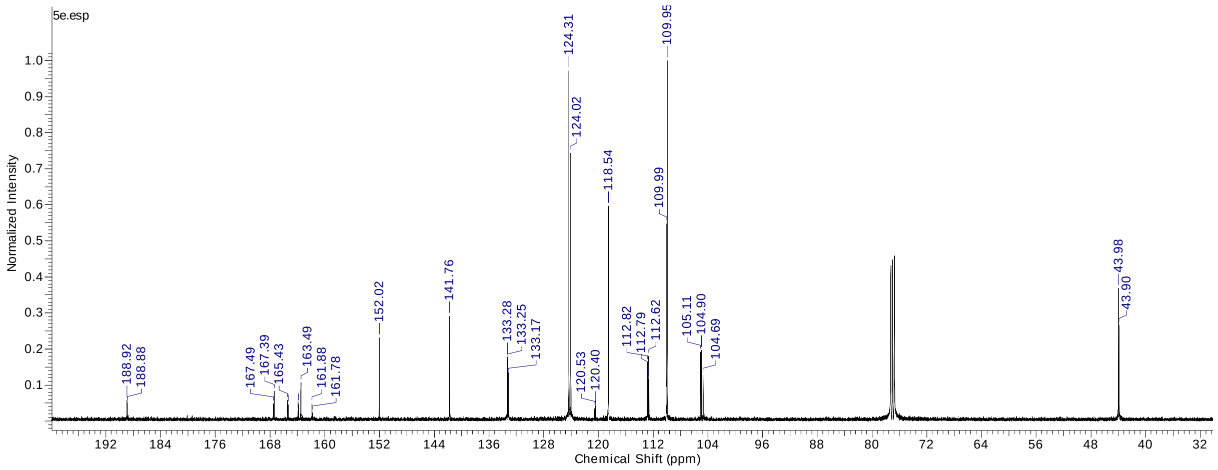

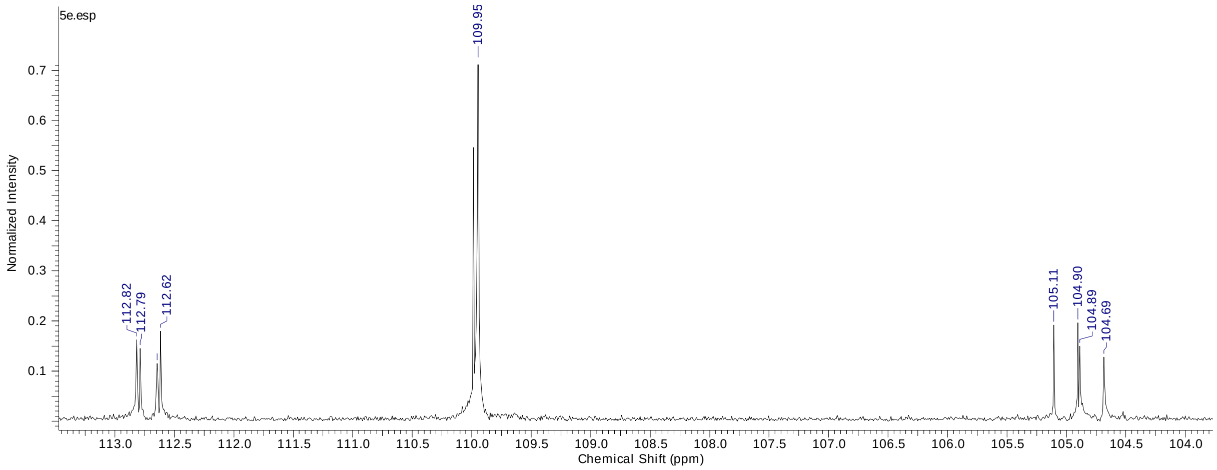
5e.esp
112.82
112.79
112.62
109.95
105.11
104.90
104.89
104.69
Normalized Intensity
Chemical Shift (ppm)

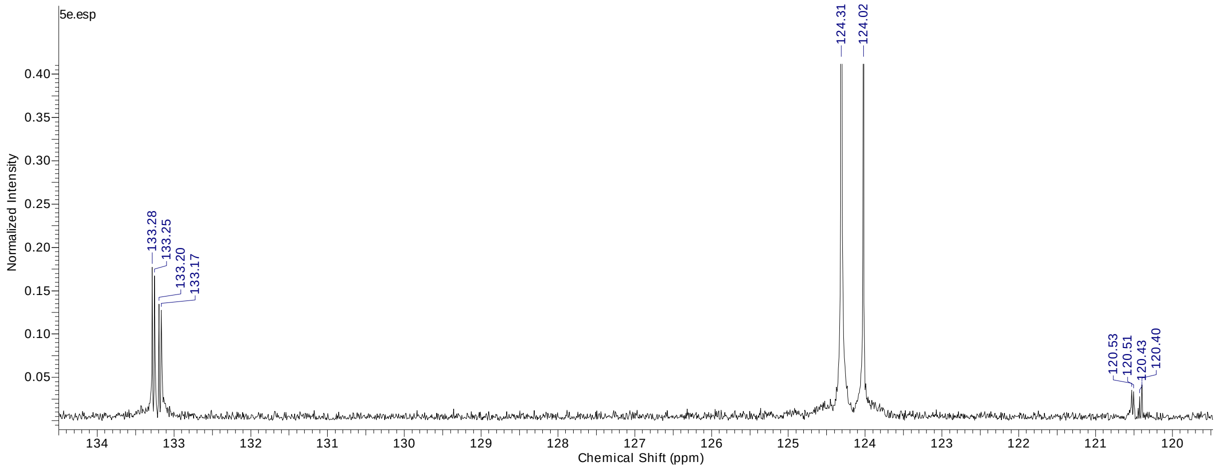
5e.esp
133.28
133.25
133.20
133.17
124.31
124.02
120.53
120.51
120.43
120.40
Normalized Intensity
Chemical Shift (ppm)

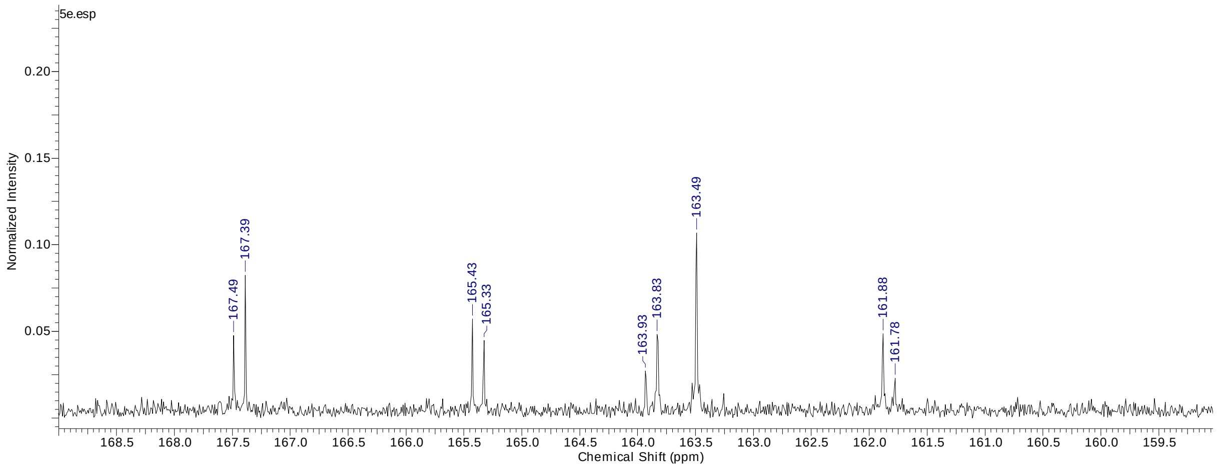
5e.esp
167.49
167.39
165.43
165.33
163.93
163.83
163.49
161.88
161.78
Normalized Intensity
Chemical Shift (ppm)

2-(1,3-benzoxazol-2-ylsulfanyl)-1-(2,4-dichlorophenyl)ethanone (**5f**)

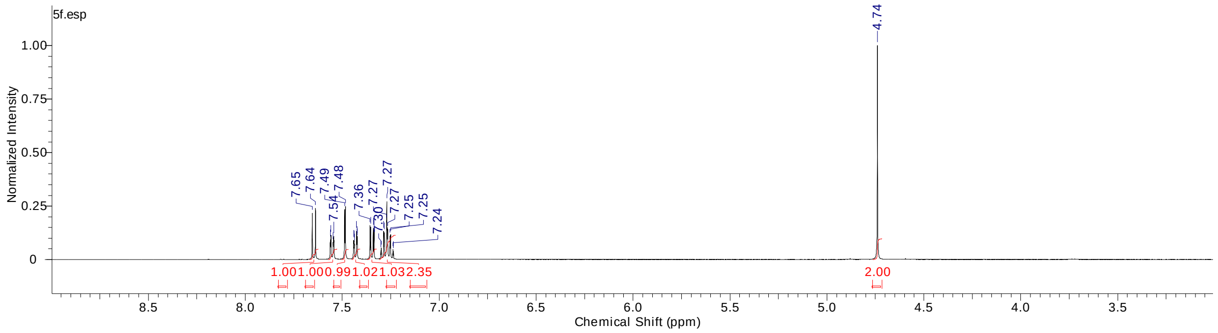

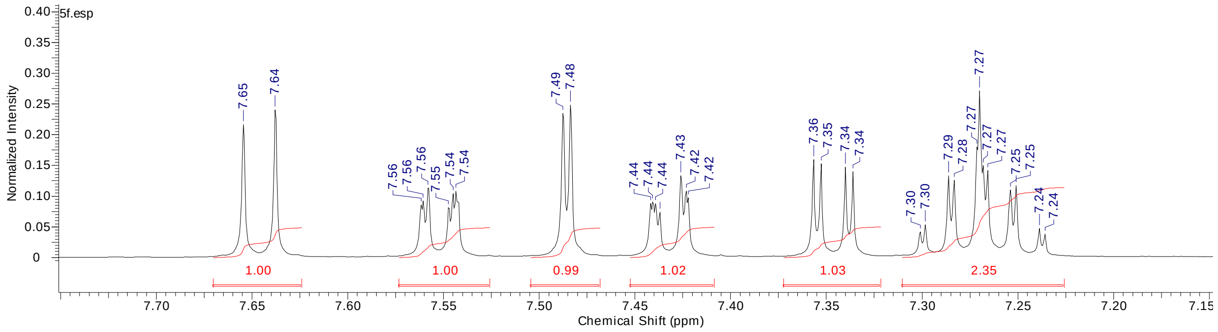

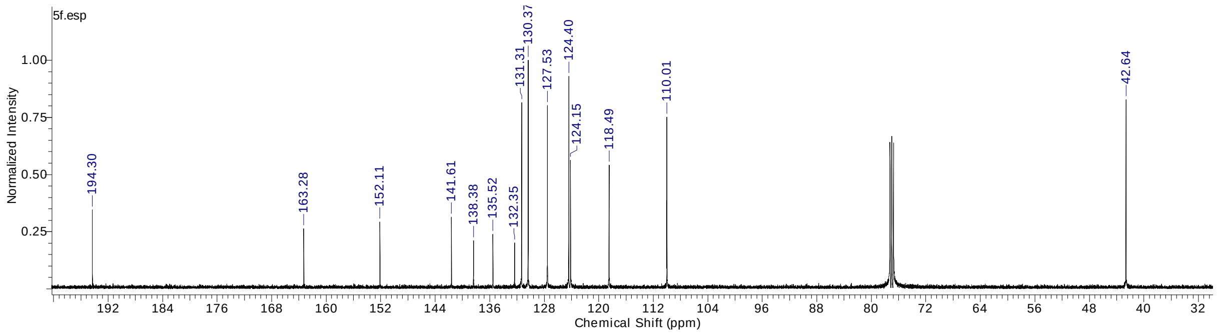

2-(1,3-benzoxazol-2-ylsulfanyl)-1-(2,5-dichlorophenyl)ethanone (**5g**)

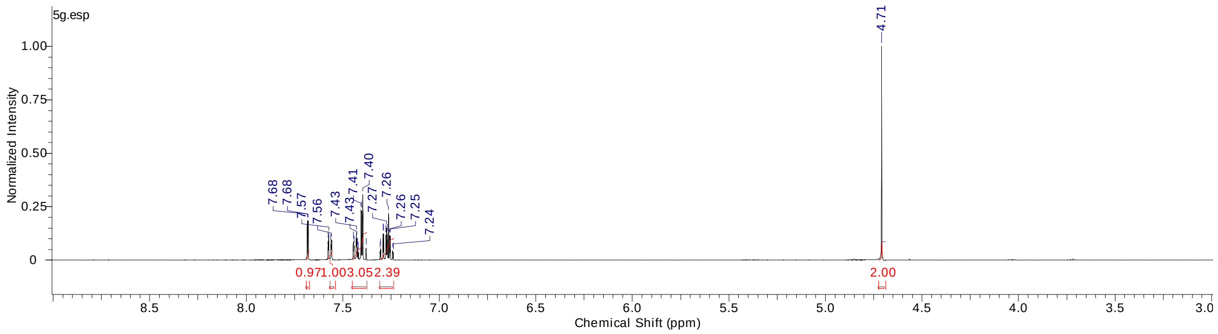

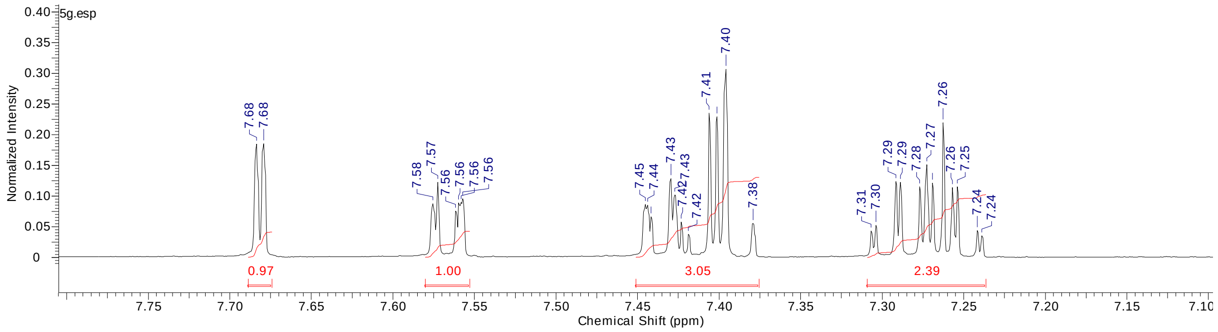

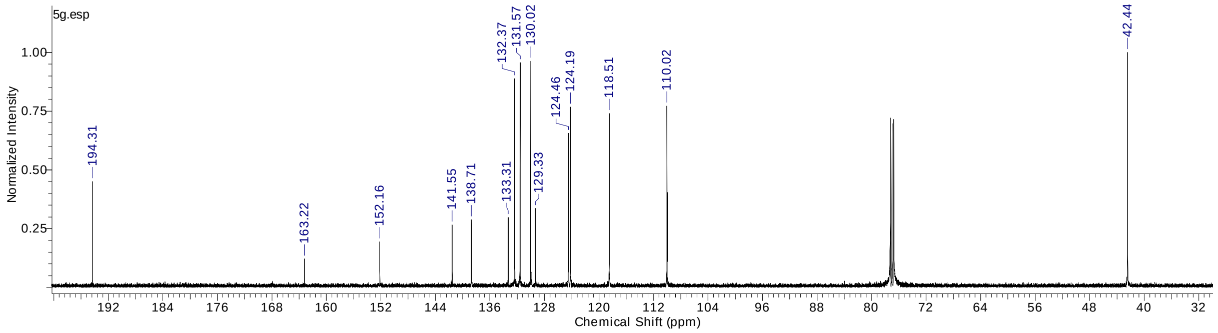

2-(1,3-benzoxazol-2-ylsulfanyl)-1-(3,4-dichlorophenyl)ethanone (**5h**)

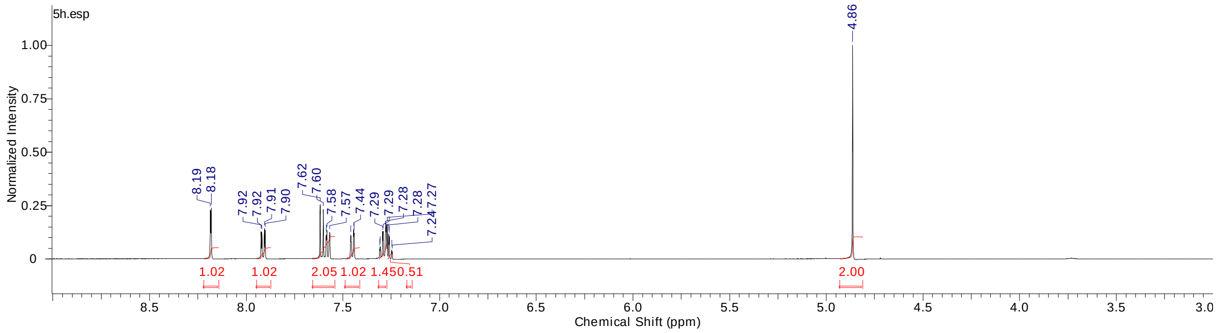

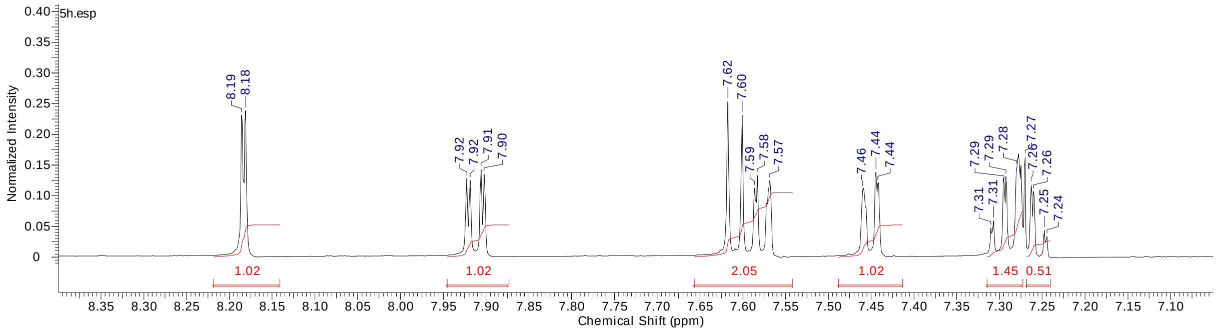

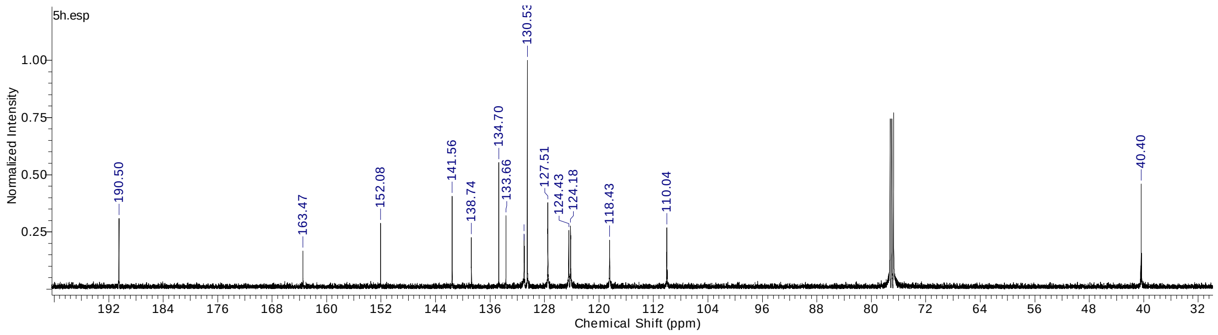

2-(1,3-benzoxazol-2-ylsulfanyl)-1-(2,3,4-trichlorophenyl)ethanone (**5i**)

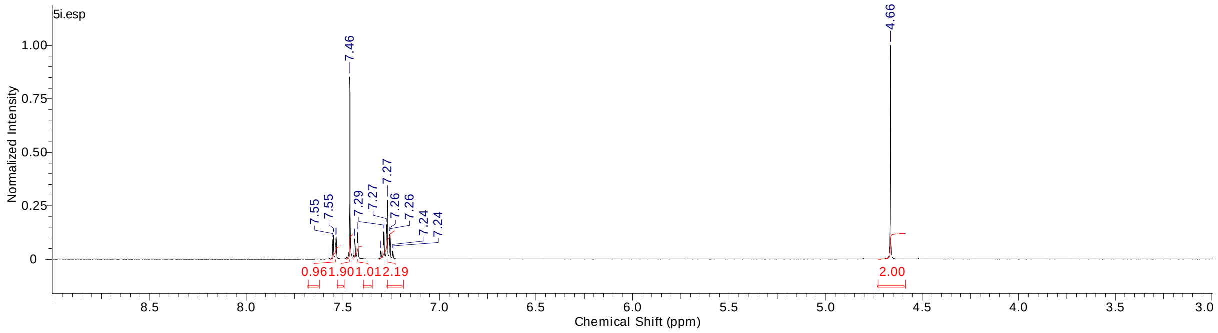

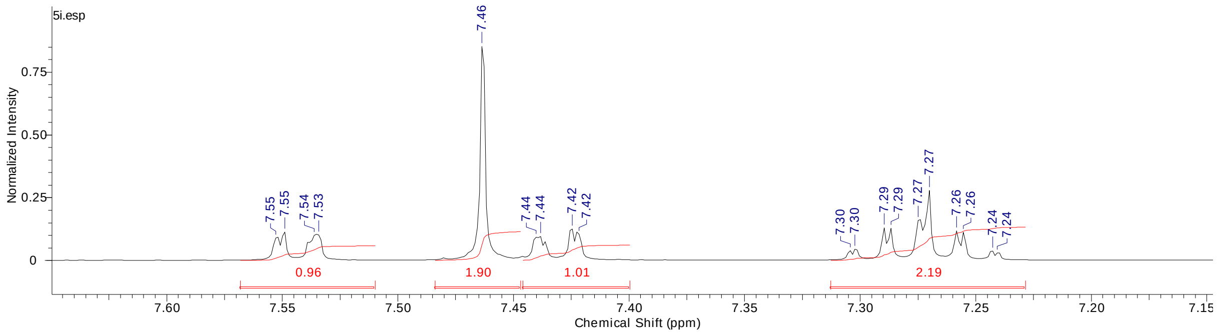

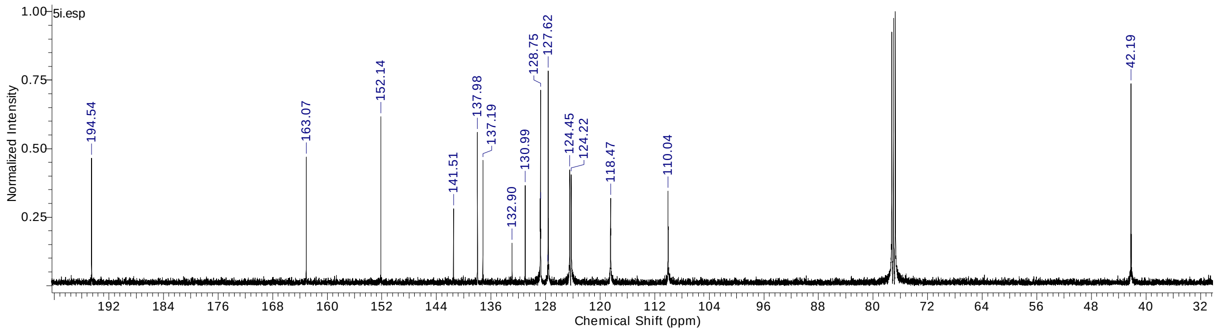

2-(1,3-benzoxazol-2-ylsulfanyl)-1-(2,4,5-trichlorophenyl)ethanone (**5j**)

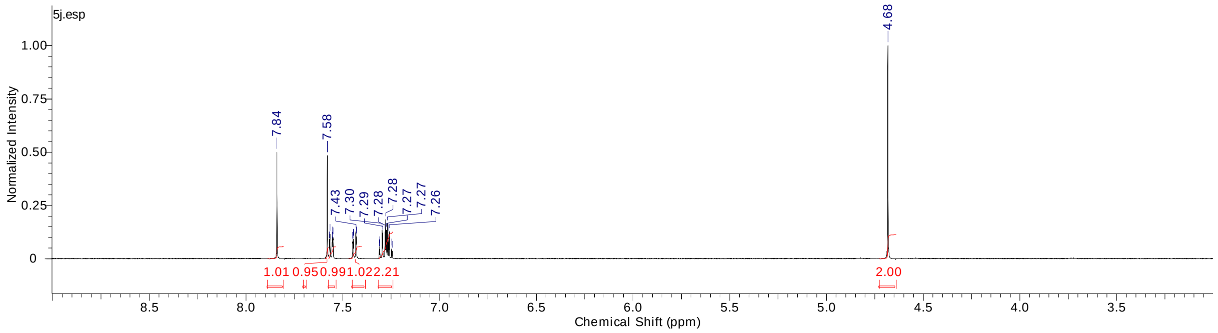

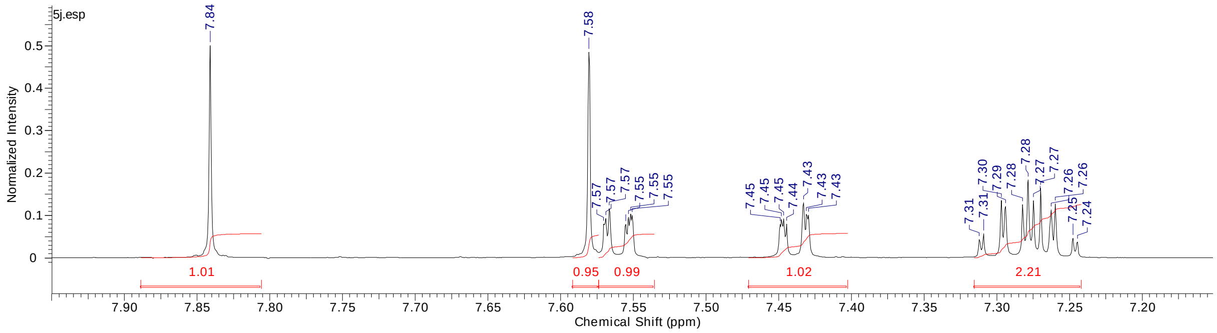

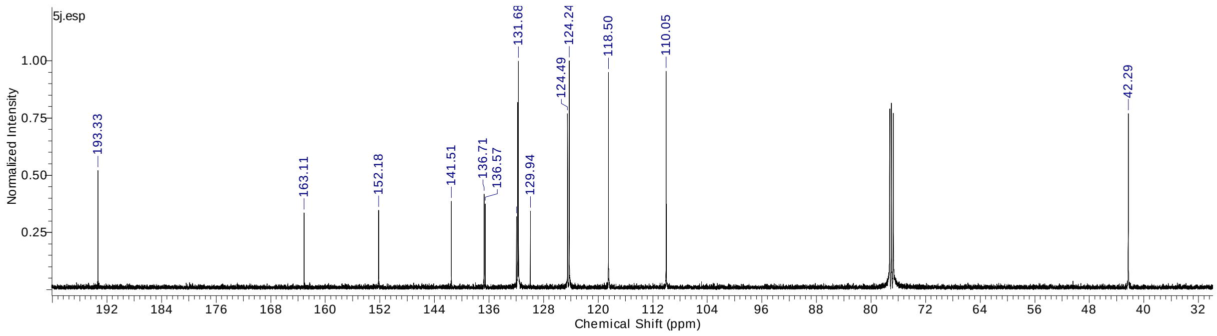

2-(1,3-benzoxazol-2-ylsulfanyl)-1-(2,4,6-trichlorophenyl)ethanone (**5k**)

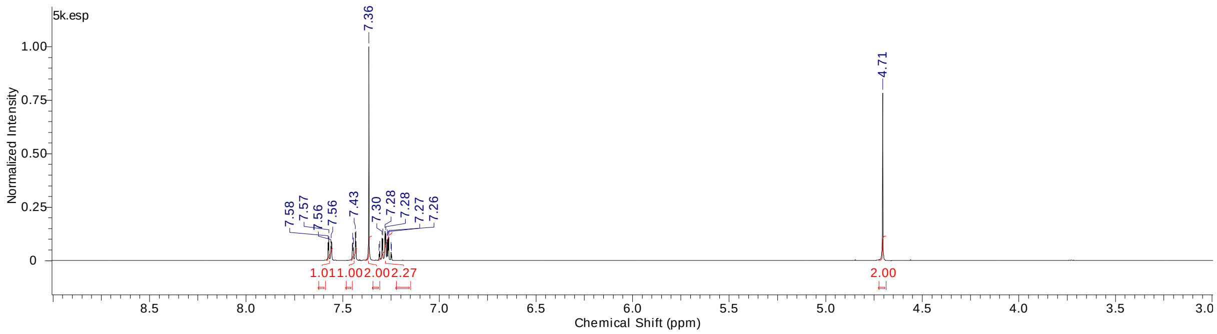

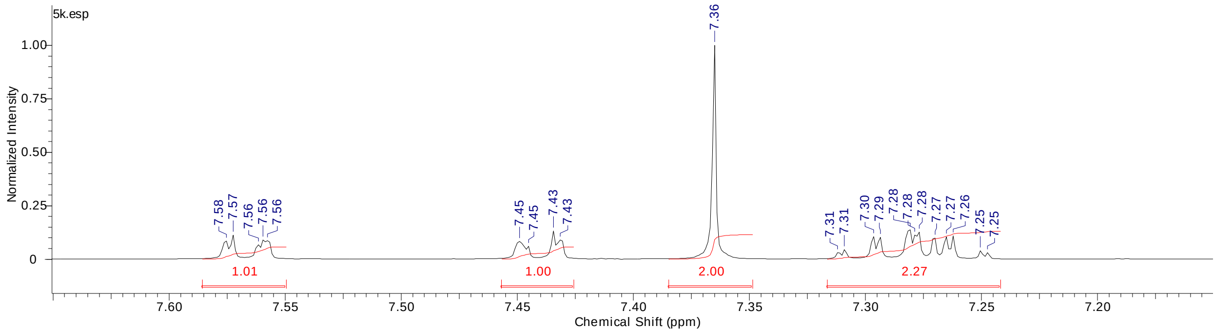

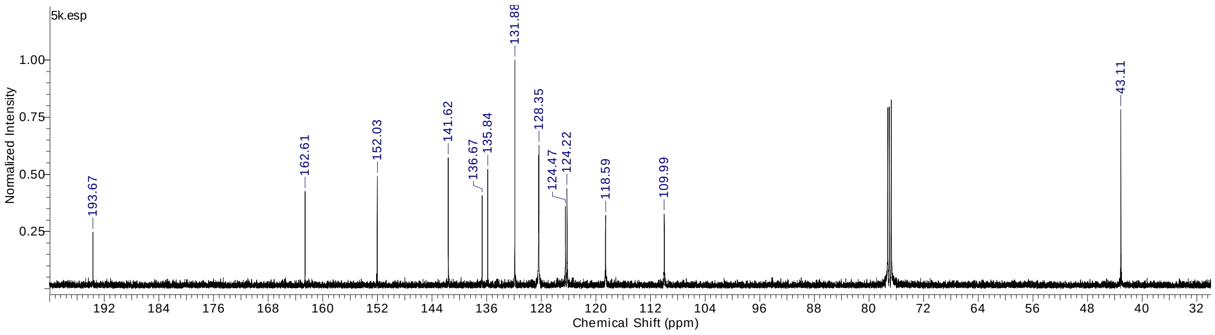

2-[(5-bromo-1,3-benzoxazol-2-yl)sulfanyl]-1-phenylethanone (**6a**)

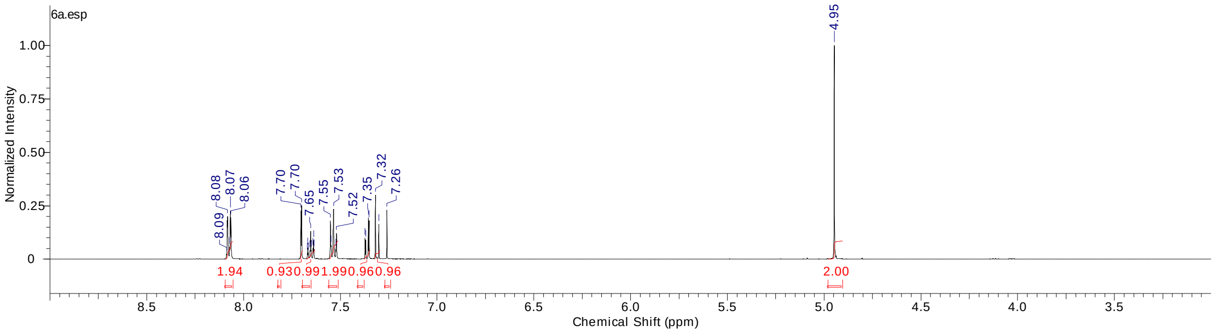

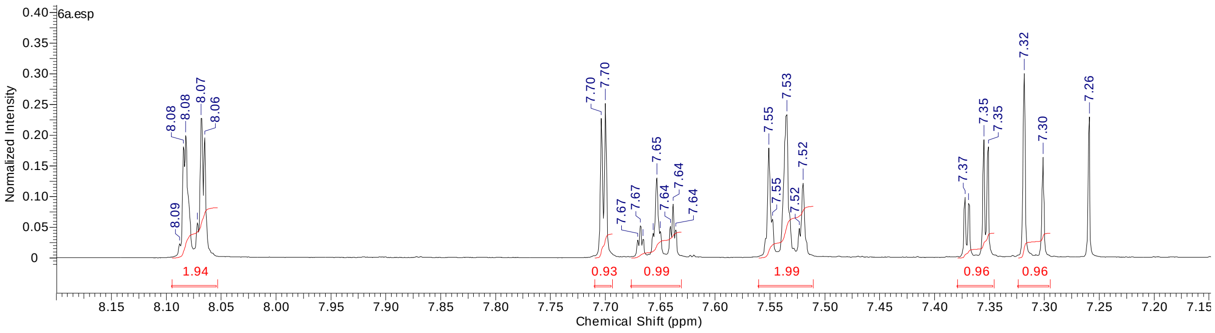

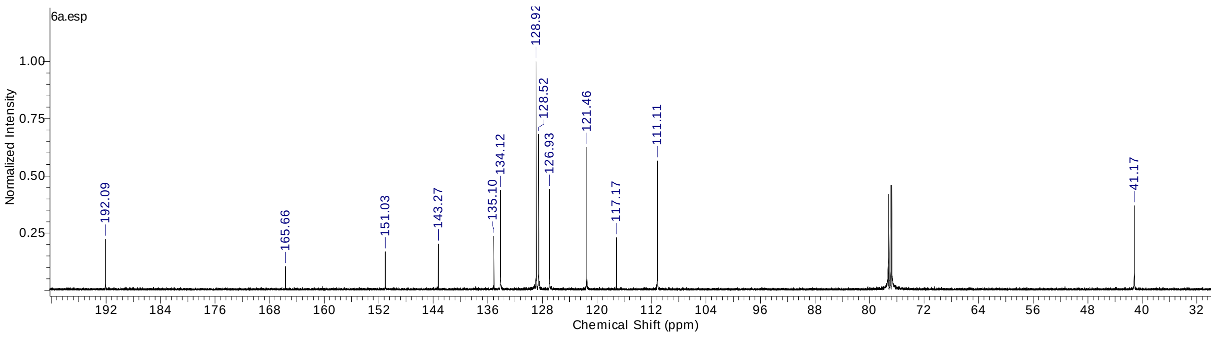

2-[(5-bromo-1,3-benzoxazol-2-yl)sulfanyl]-1-(4-fluorophenyl)ethanone (**6b**)

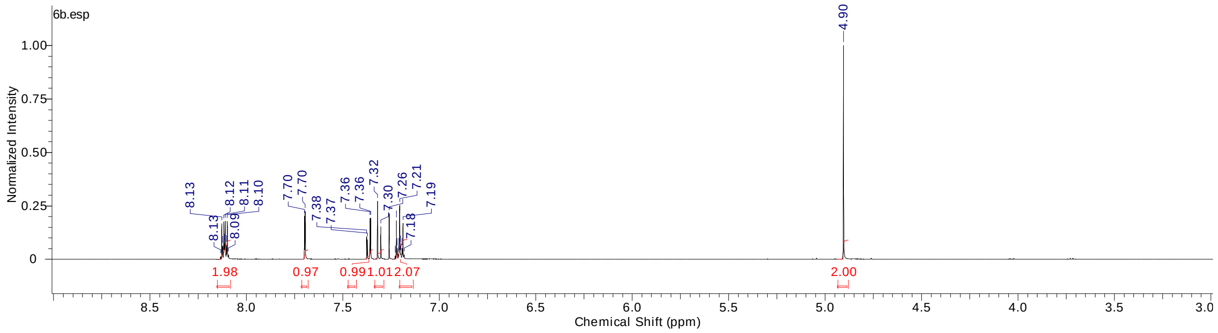

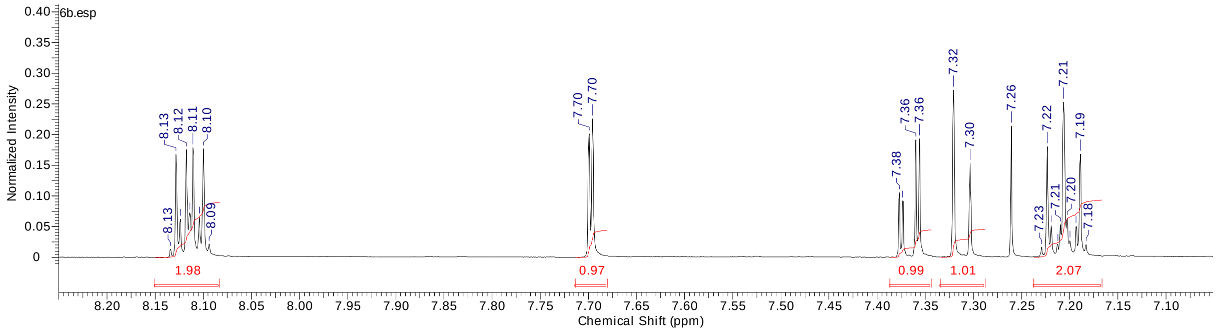

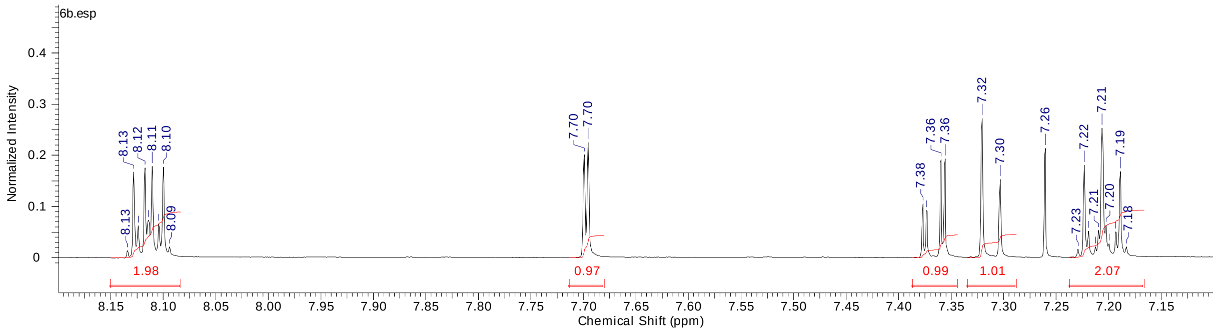

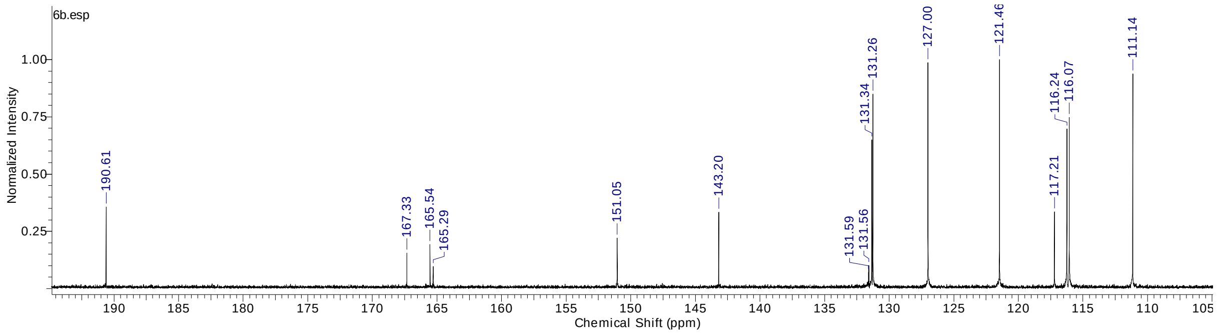

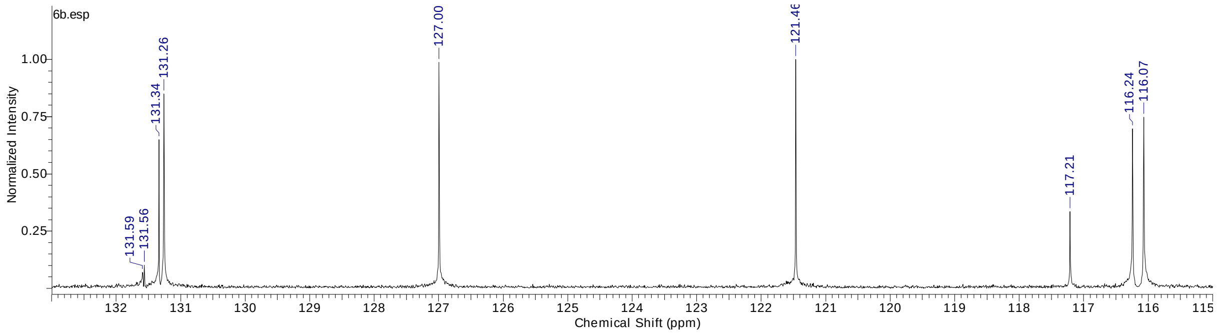

2-[(5,7-dibromo-1,3-benzoxazol-2-yl)sulfanyl]-1-phenylethanone (**7a**)

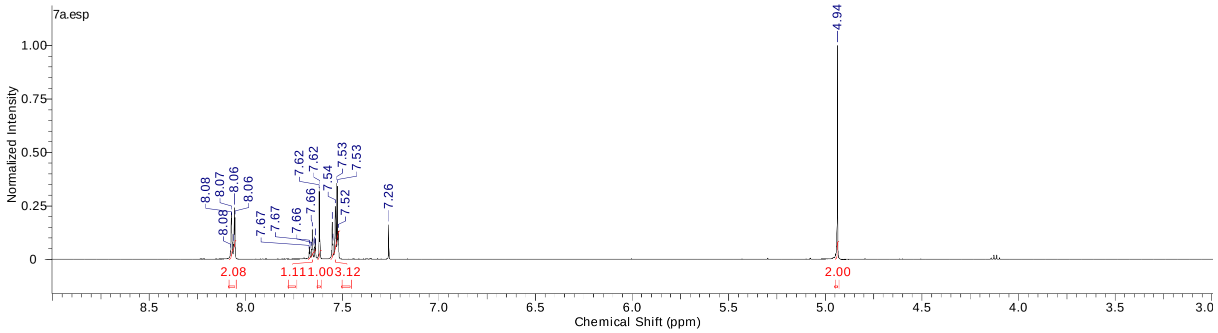

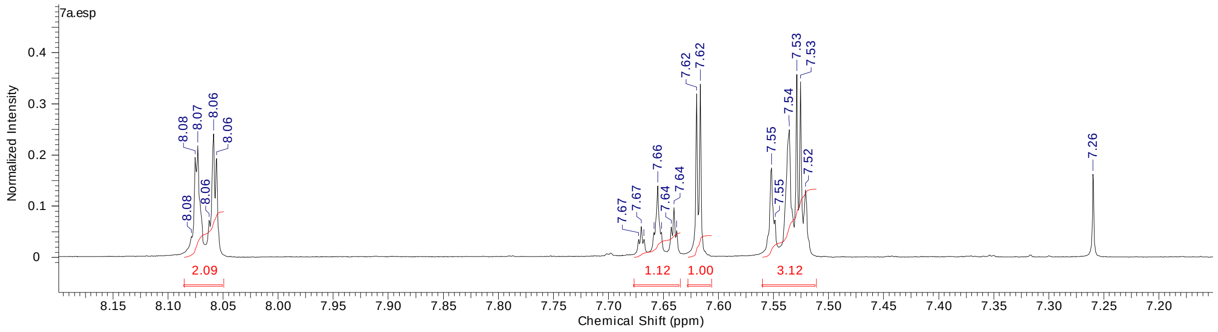

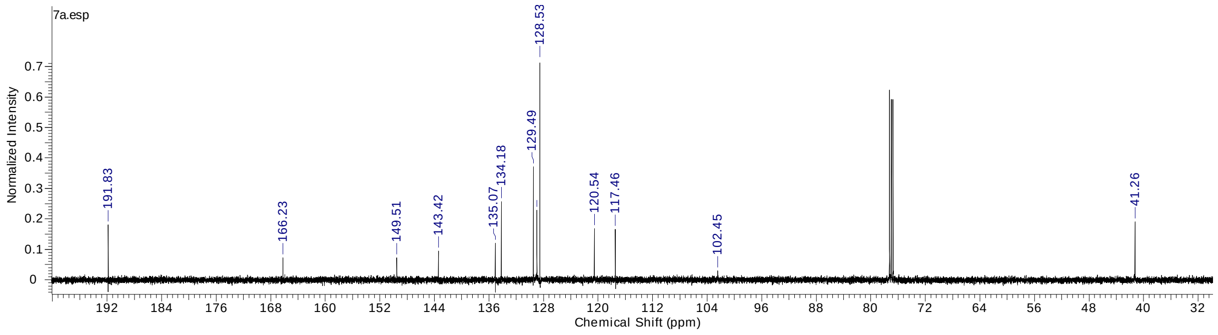

2-[(5,7-dibromo-1,3-benzoxazol-2-yl)sulfanyl]-1-(4-fluorophenyl)ethanone (**7b**)

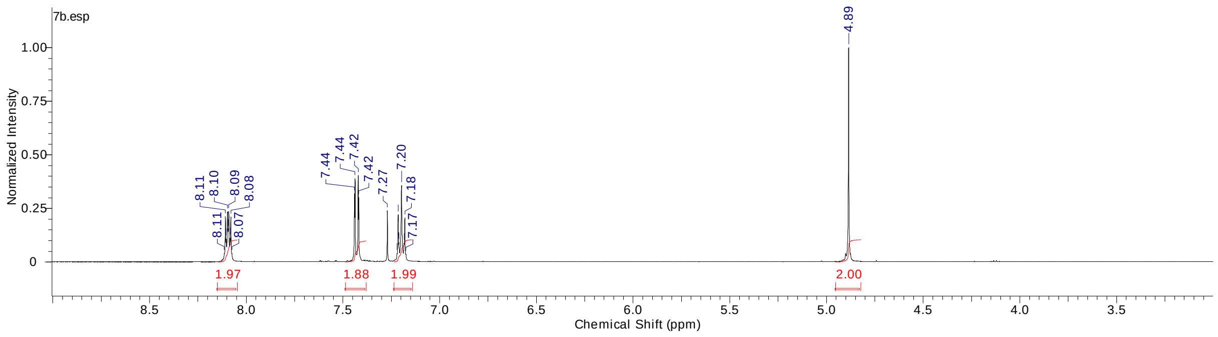

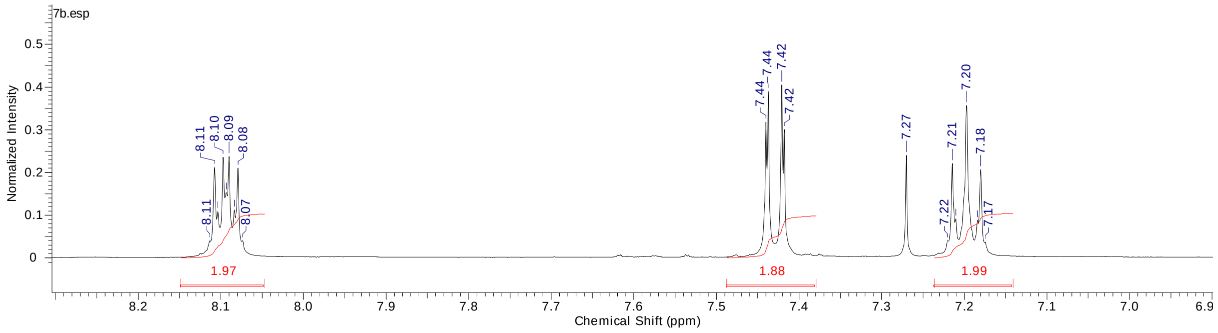

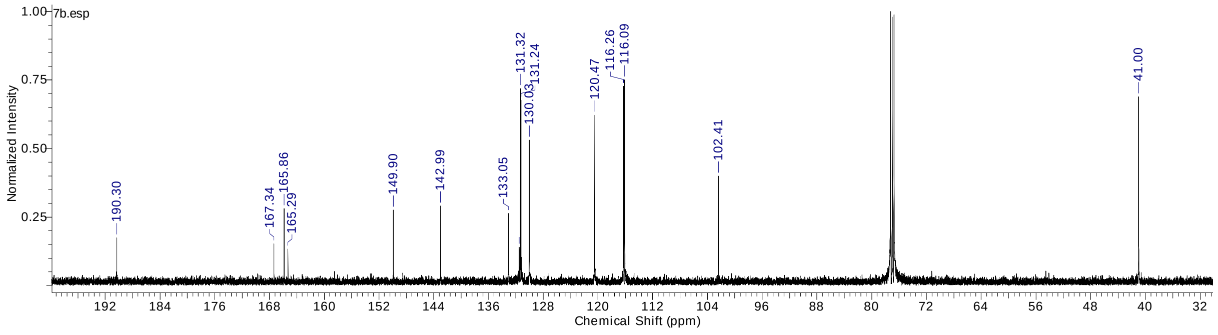

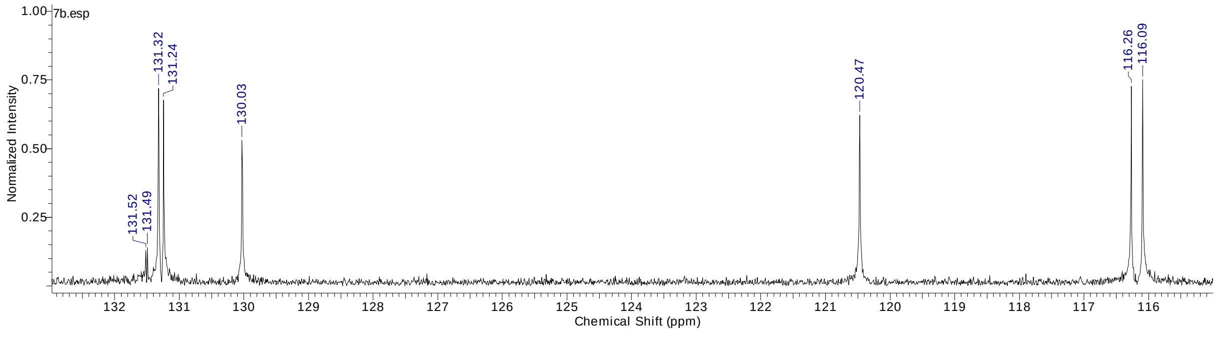

2-(1,3-benzoxazol-2-ylsulfanyl)-1-(4-fluorophenyl)ethanol (**8b**)

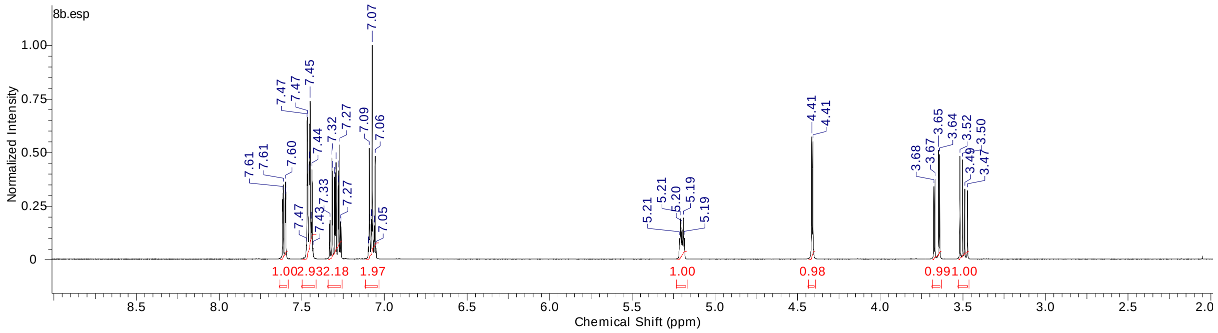

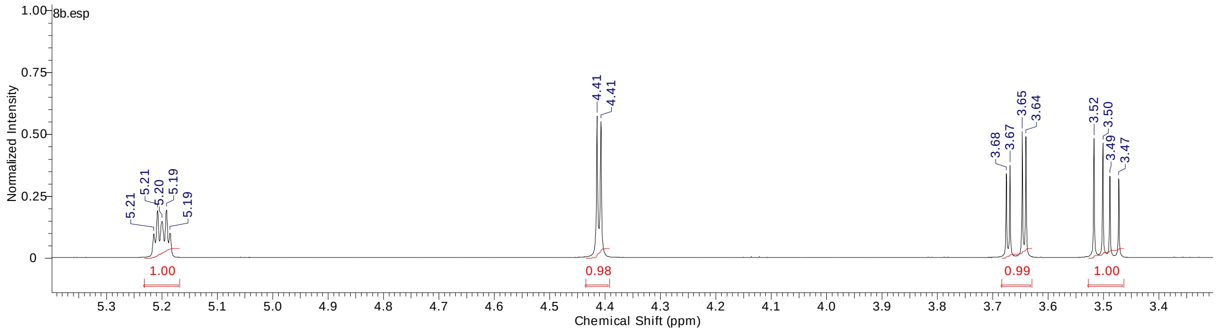
8b.esp
Normalized Intensity
Chemical Shift (ppm)
5.21 5.21 5.20 5.19 5.19
4.41 4.41
3.68 3.67 3.65 3.64
3.52 3.50 3.49 3.47
1.00 0.98 0.99 1.00

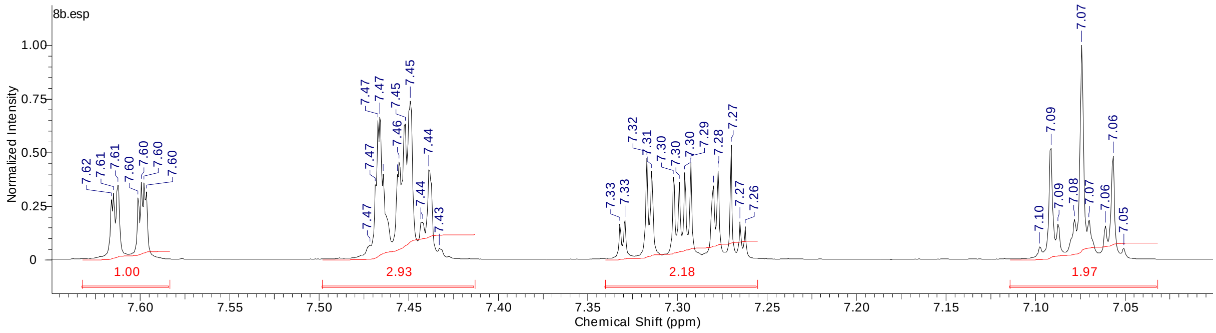
8b.esp
Normalized Intensity
Chemical Shift (ppm)
7.62 7.61 7.61 7.60 7.60 7.60 7.60
7.47 7.47 7.47 7.47 7.46 7.45 7.45 7.44 7.44 7.43
7.33 7.33 7.32 7.31 7.30 7.30 7.30 7.29 7.28 7.27 7.27 7.26
7.10 7.09 7.09 7.08 7.07 7.07 7.06 7.06 7.05
1.00 2.93 2.18 1.97

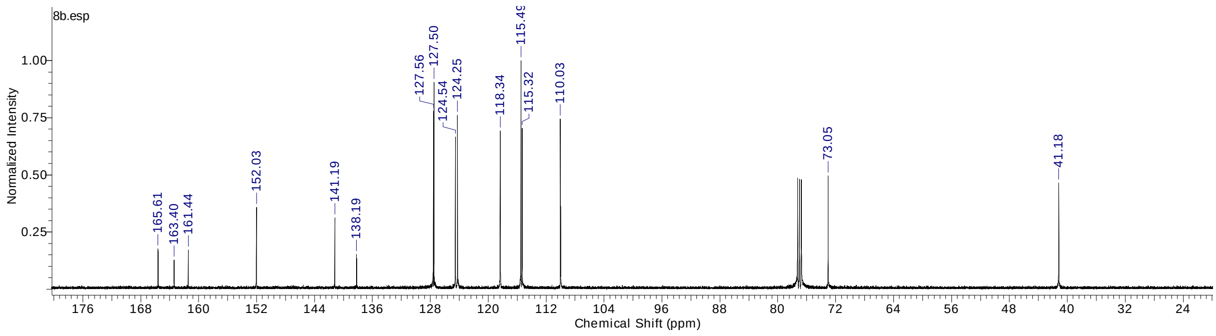
8b.esp
Normalized Intensity
Chemical Shift (ppm)
165.61 163.40 161.44 152.03 141.19 138.19 127.56 127.50 124.54 124.25 118.34 115.49 115.32 110.03 73.05 41.18

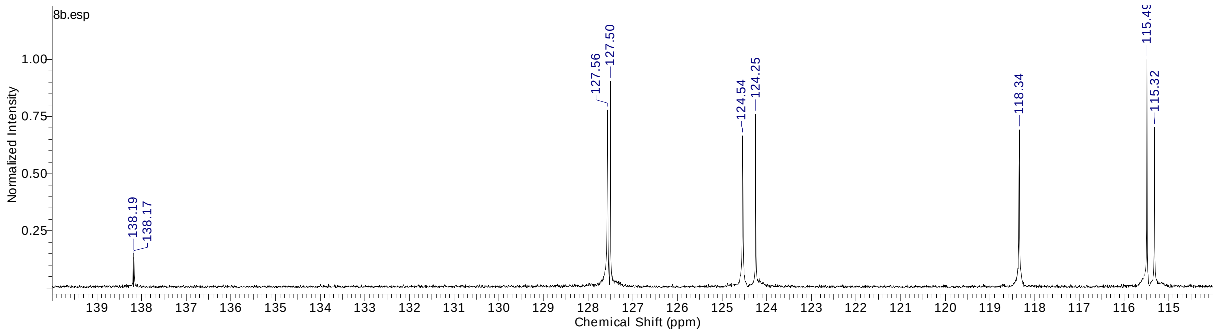
8b.esp
Normalized Intensity
Chemical Shift (ppm)
138.19 138.17 127.56 127.50 124.54 124.25 118.34 115.49 115.32

2-(1,3-benzoxazol-2-ylsulfanyl)-1-(4-chlorophenyl)ethanol (**8c**)

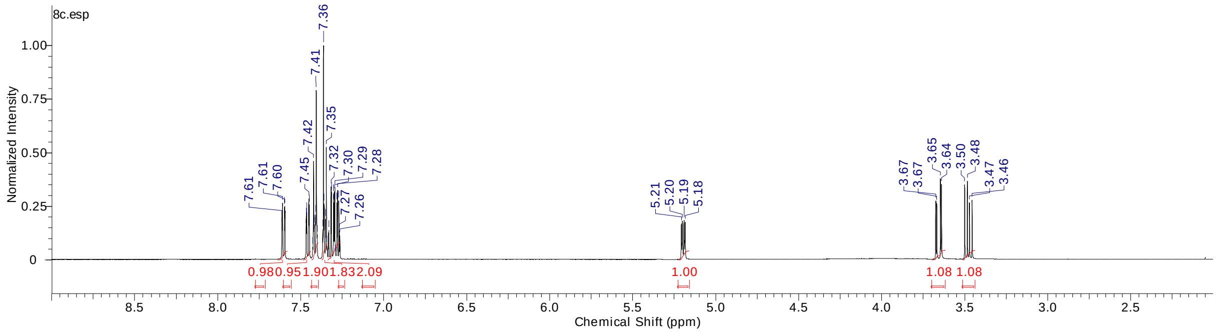

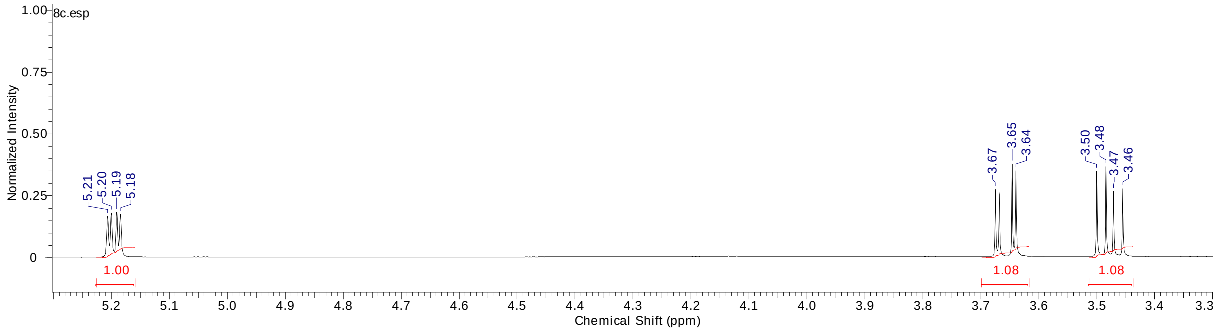

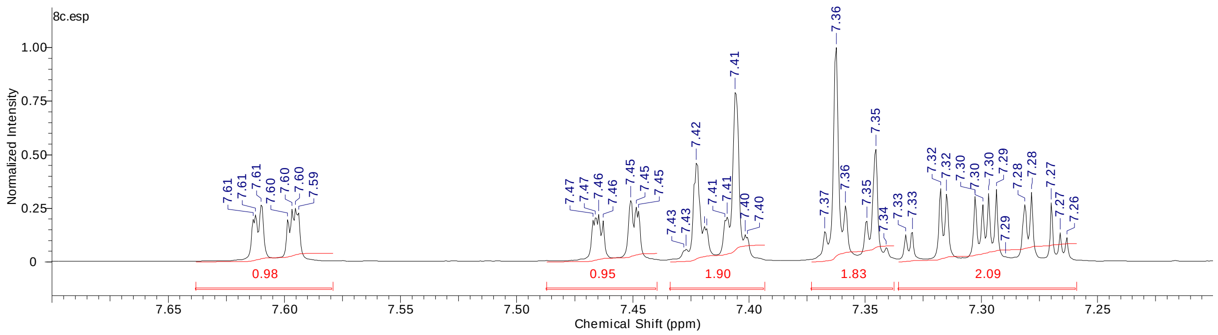

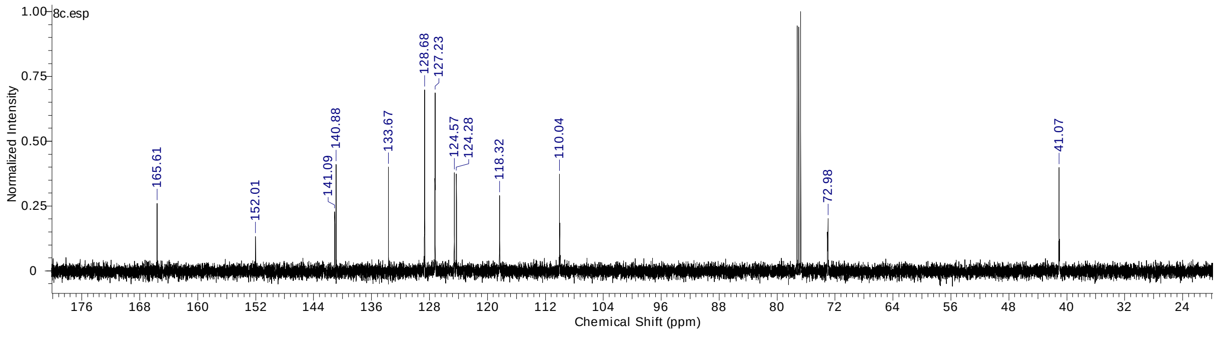

2-(1,3-benzoxazol-2-ylsulfanyl)-1-(4-bromophenyl)ethanol (**8d**)

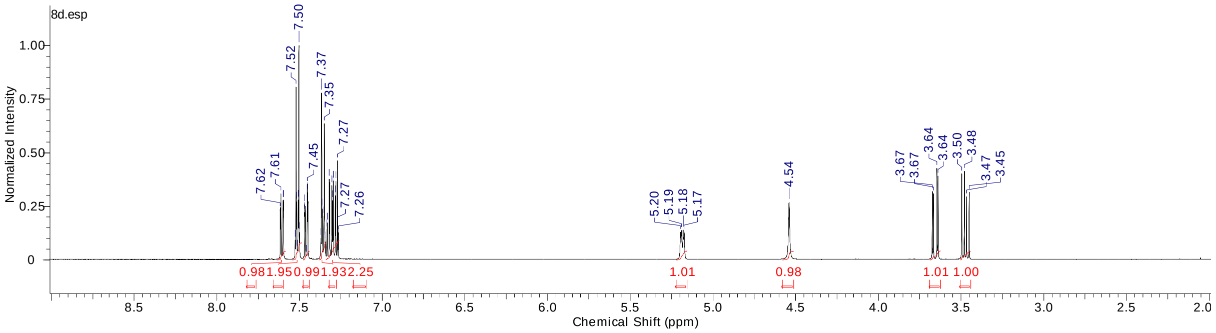

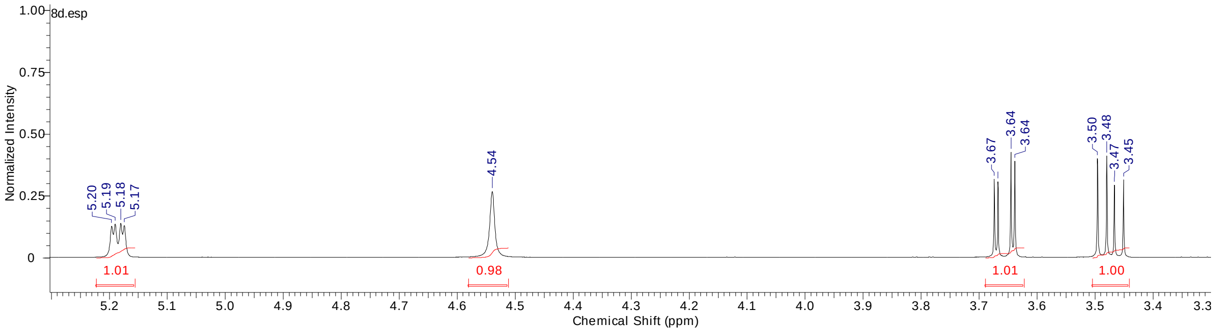

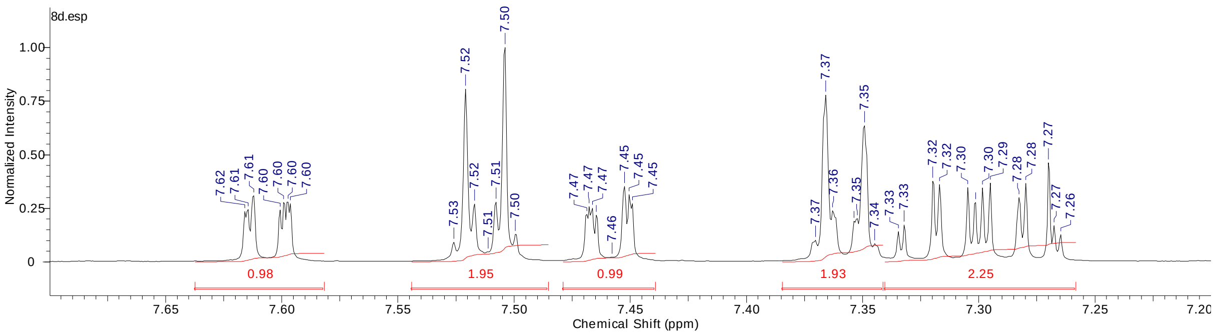

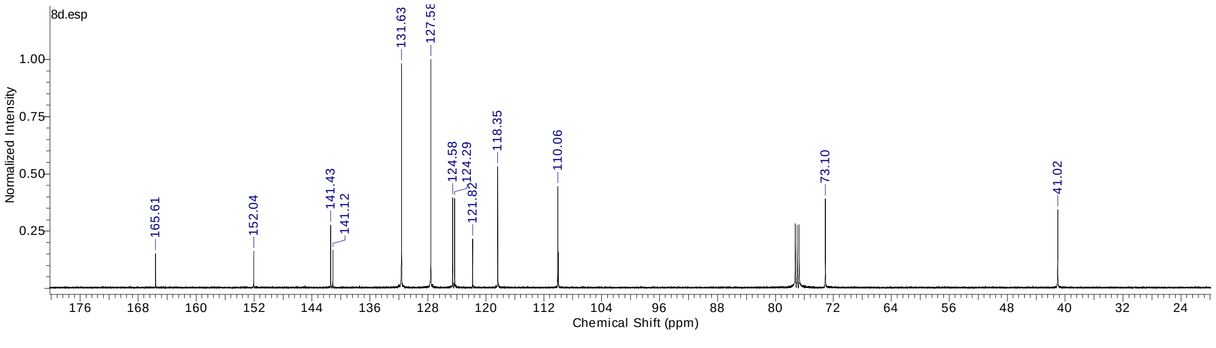

2-(1,3-benzoxazol-2-ylsulfanyl)-1-(2,4-difluorophenyl)ethanol (**8e**)

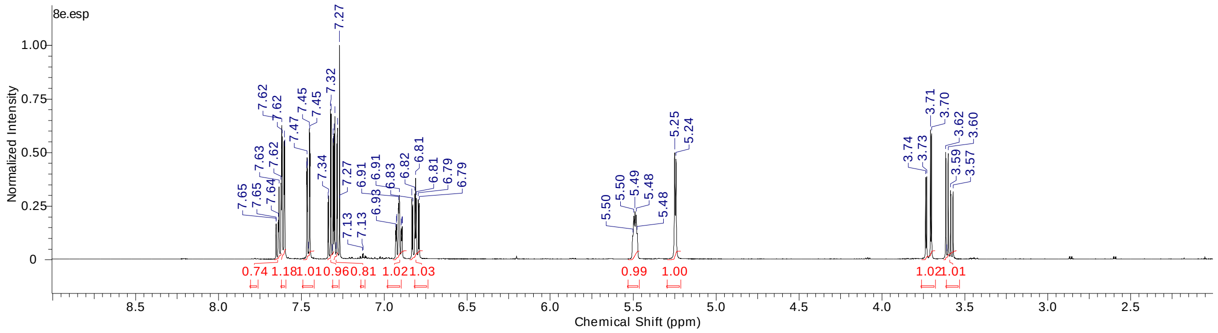

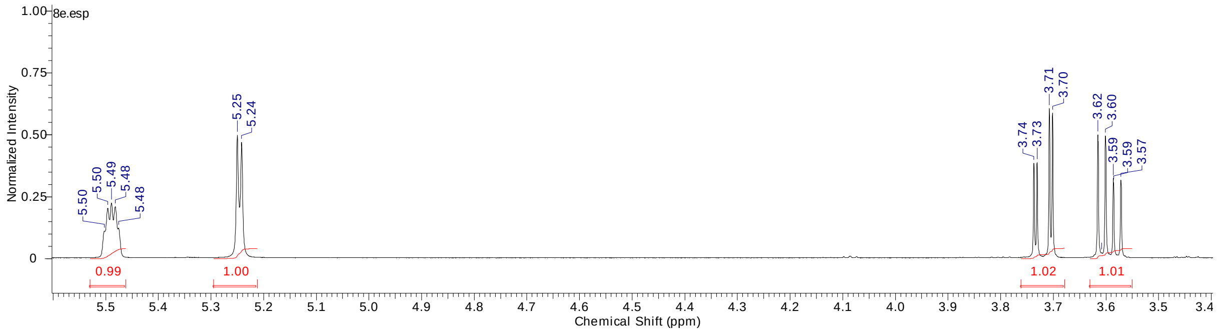

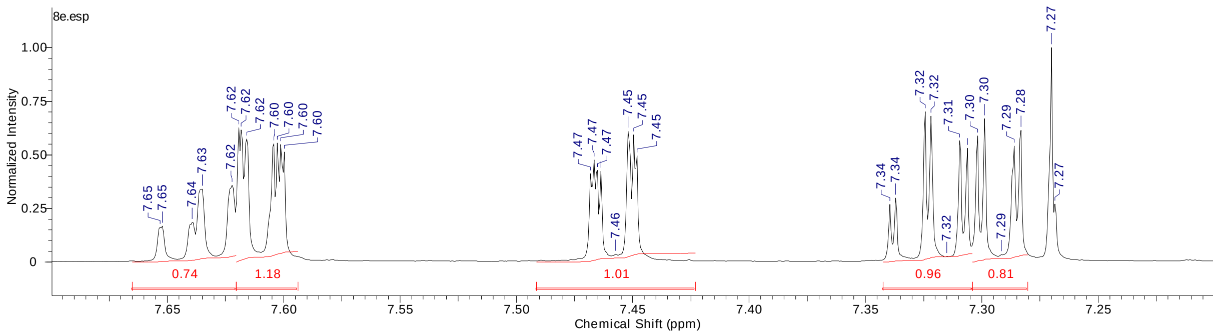

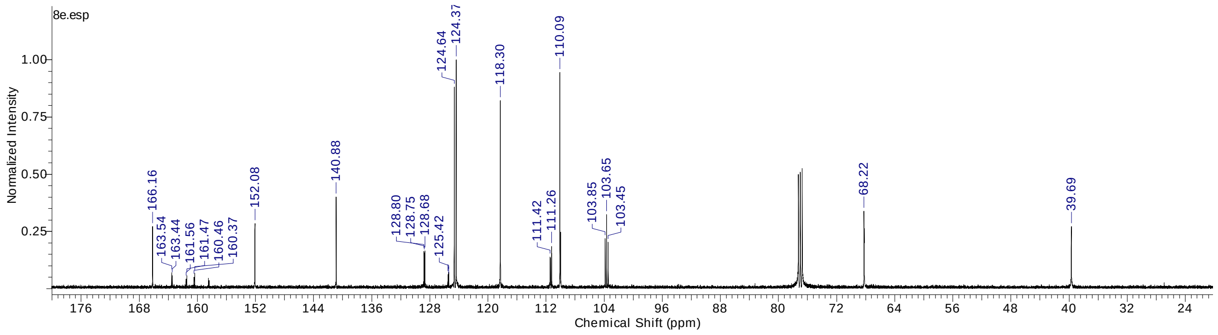

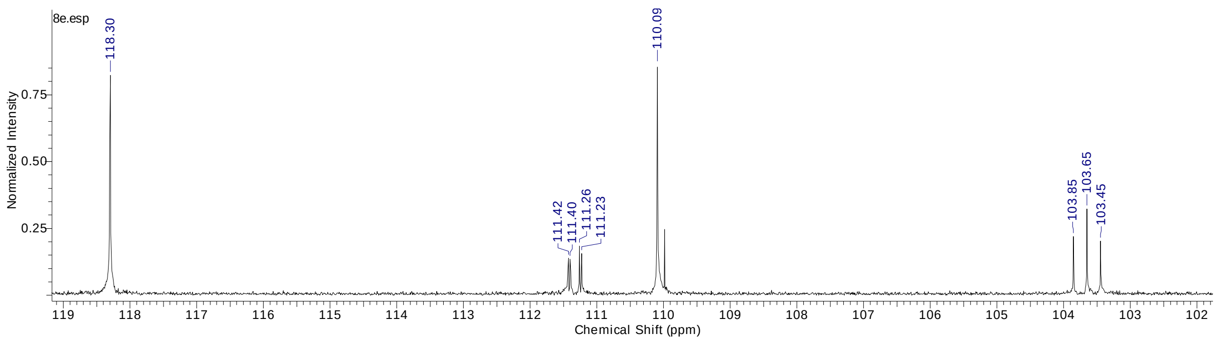

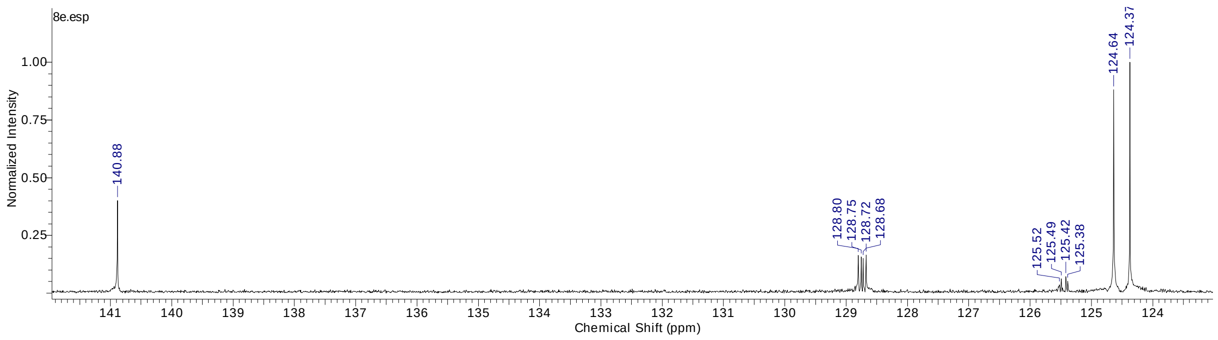

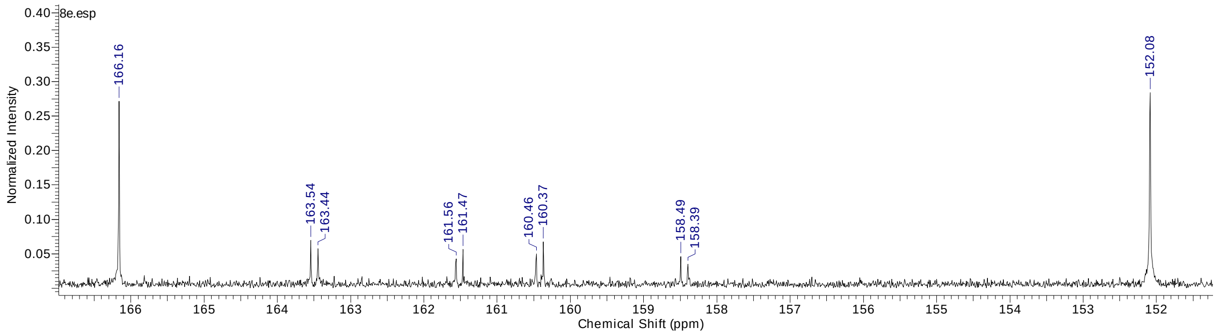

2-(1,3-benzoxazol-2-ylsulfanyl)-1-(2,4-dichlorophenyl)ethanol (**8f**)

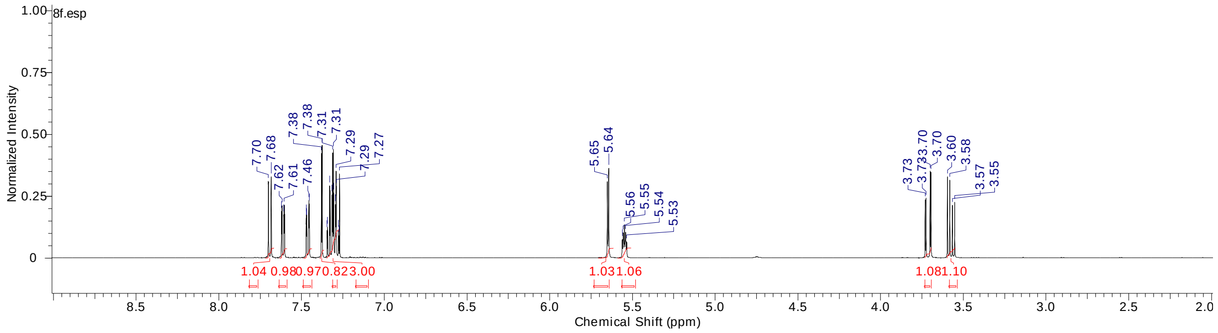

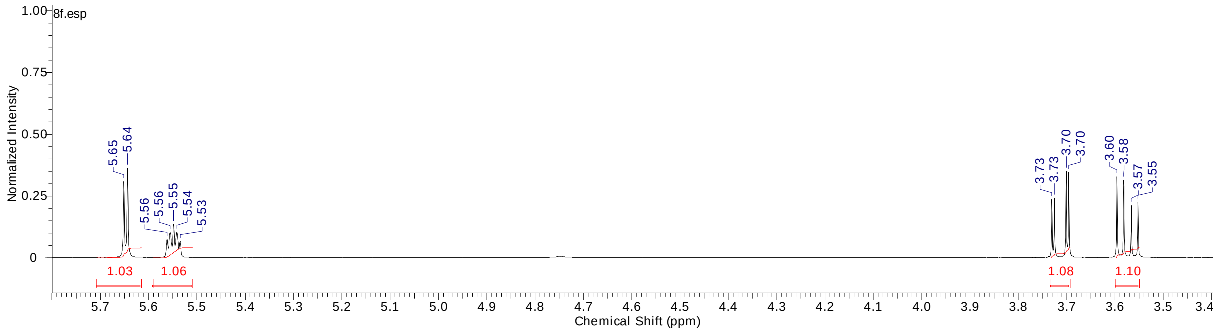

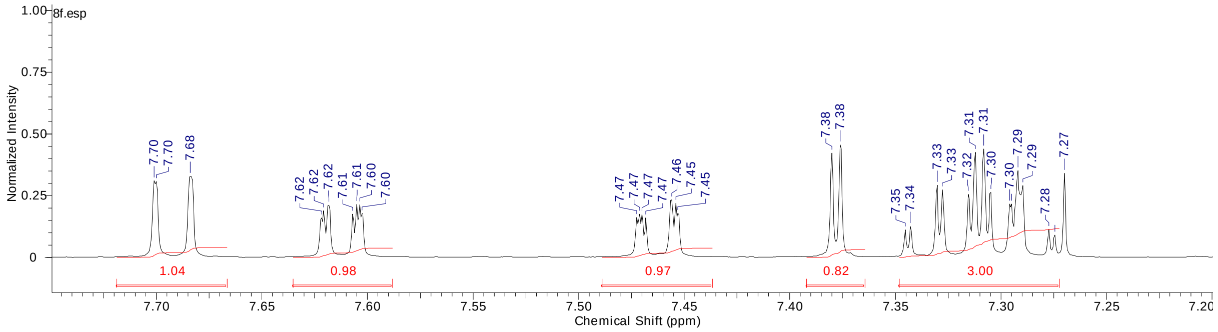

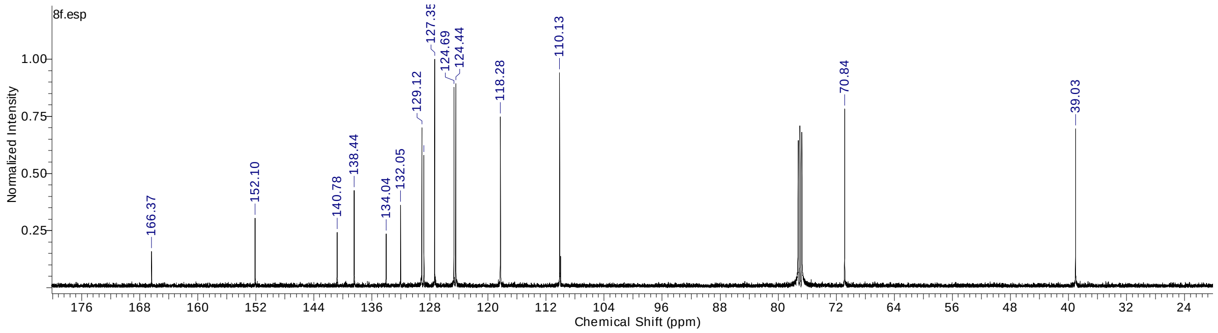

2-(1,3-benzoxazol-2-ylsulfanyl)-1-(3,4-dichlorophenyl)ethanol (**8h**)

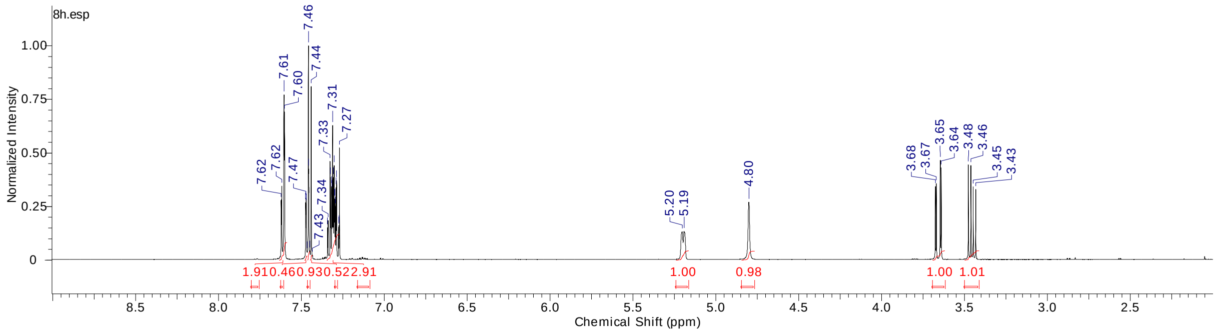

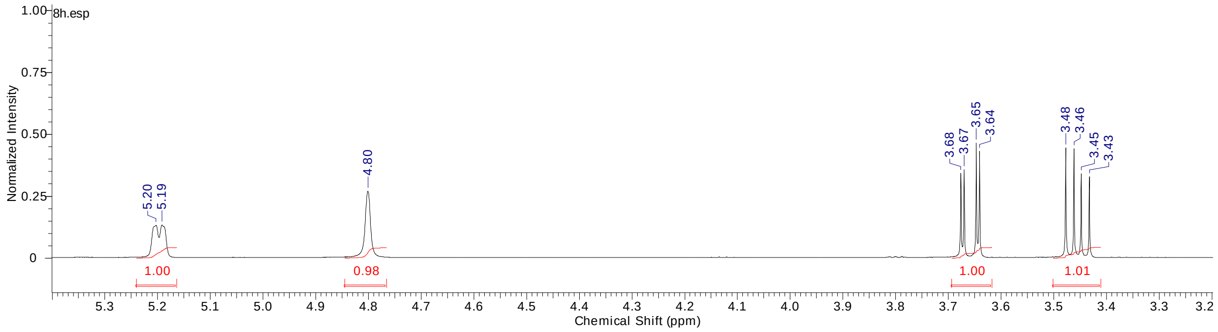

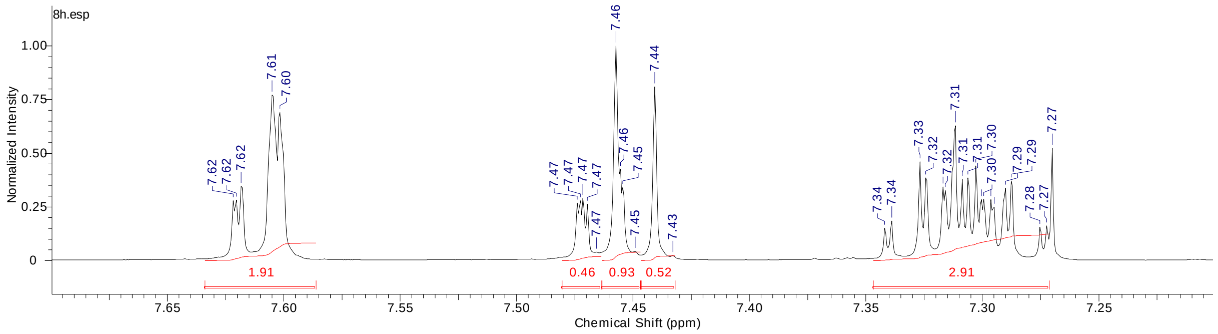

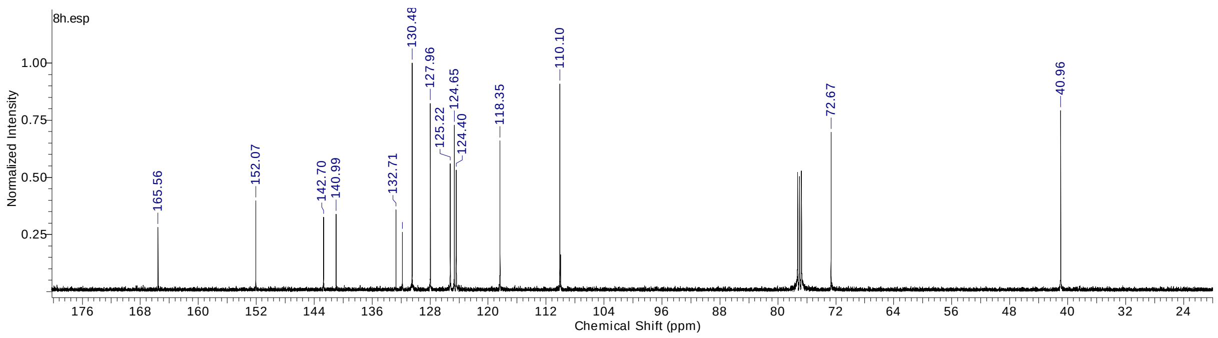

2-(1,3-benzoxazol-2-ylsulfanyl)-1-(2,3,4-trichlorophenyl)ethanol (**8i**)

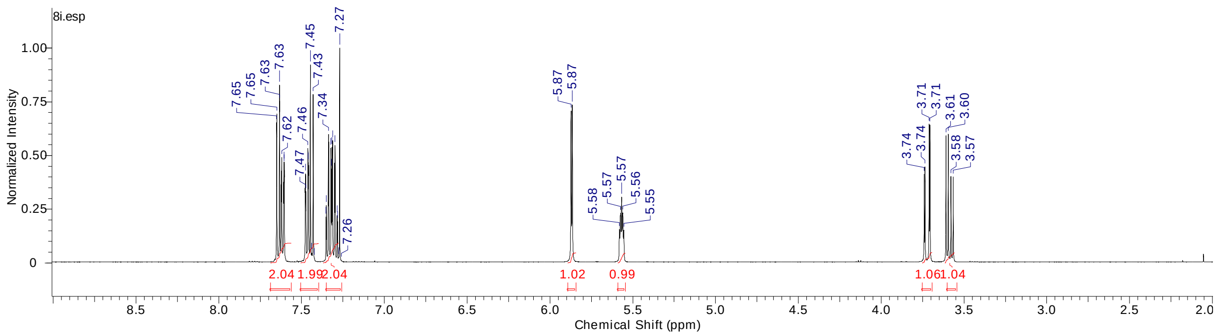

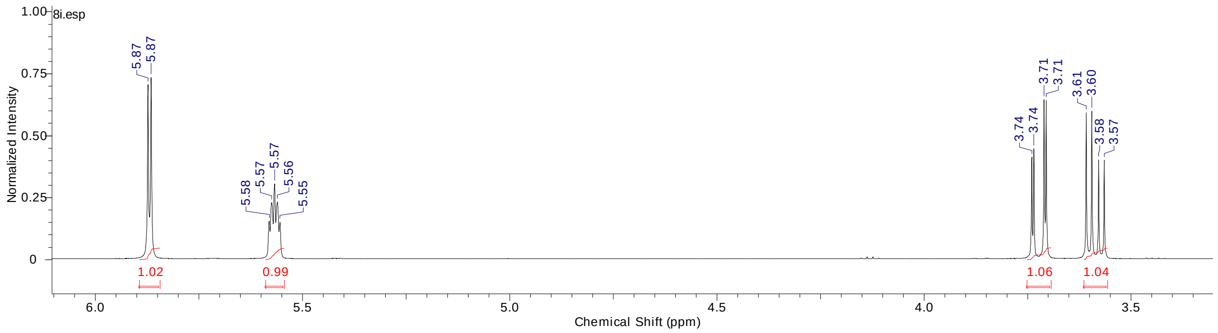

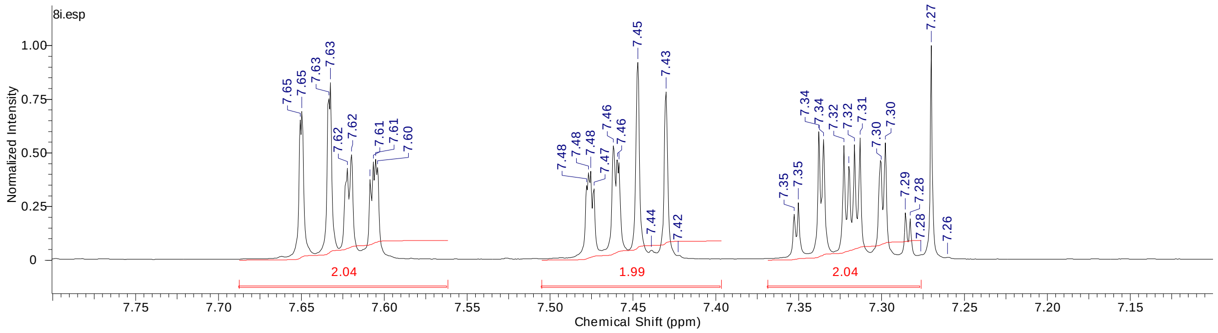

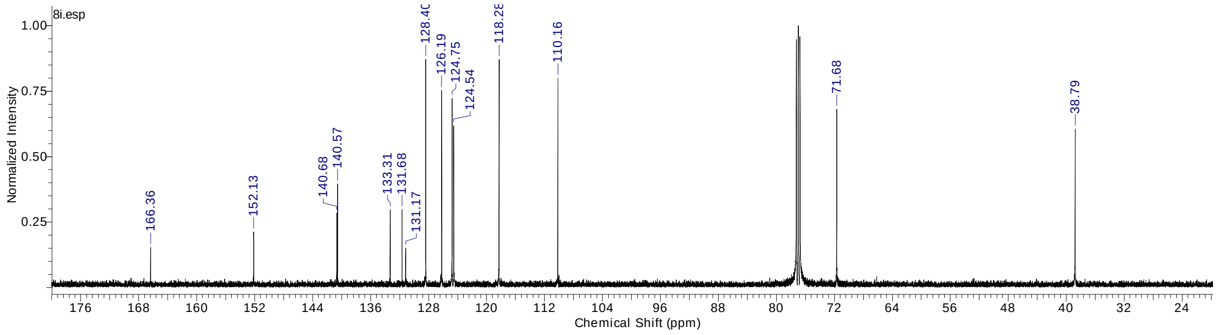

2-(1,3-benzoxazol-2-ylsulfanyl)-1-(2,4,5-trichlorophenyl)ethanol (**8j**)

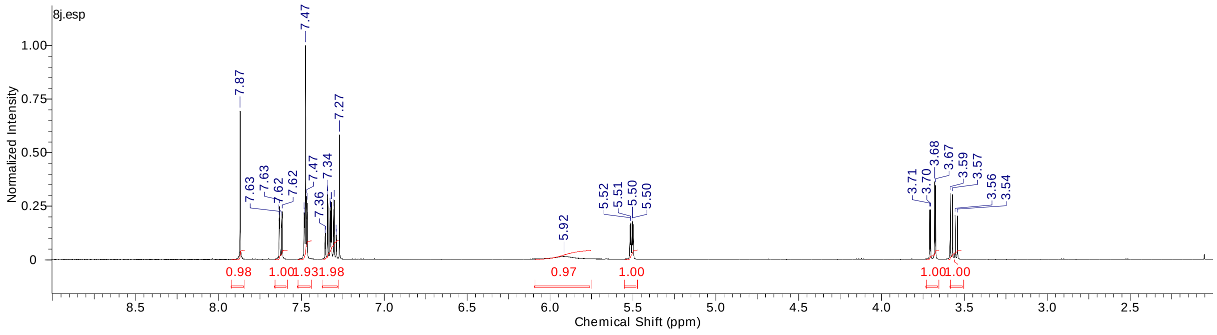

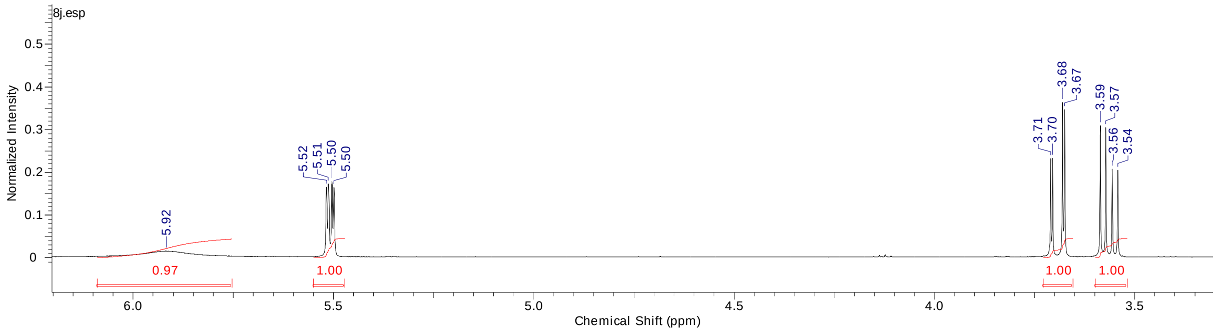

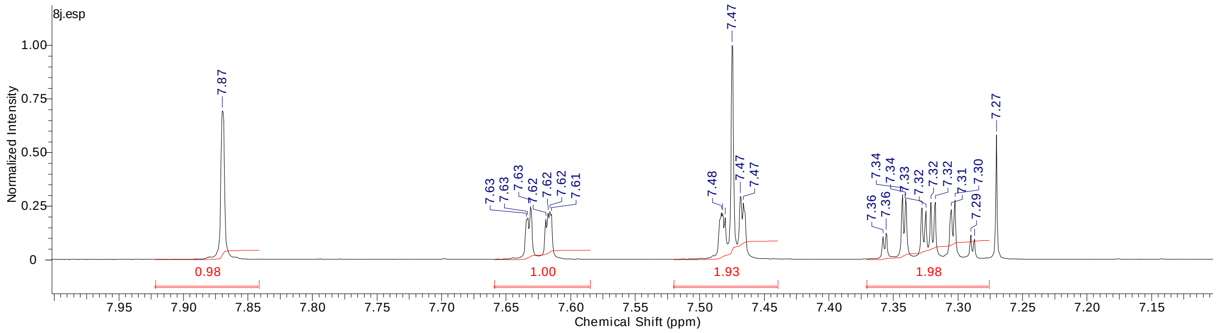

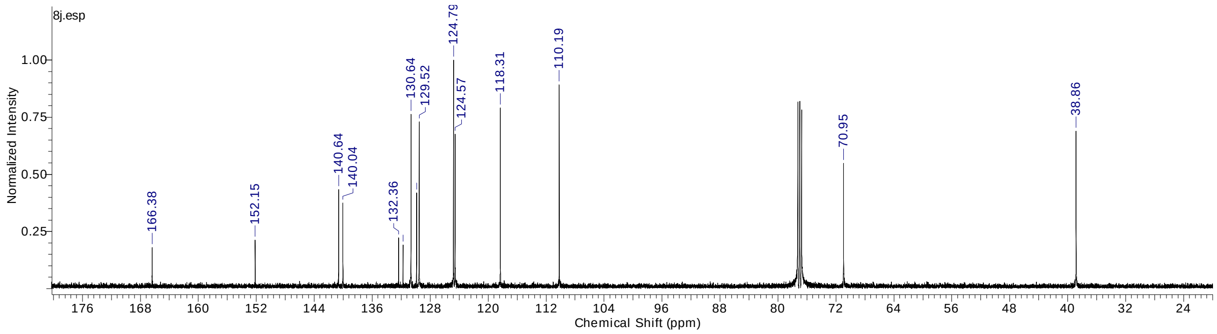

3. HR-MS analysis of compounds 5 – 8

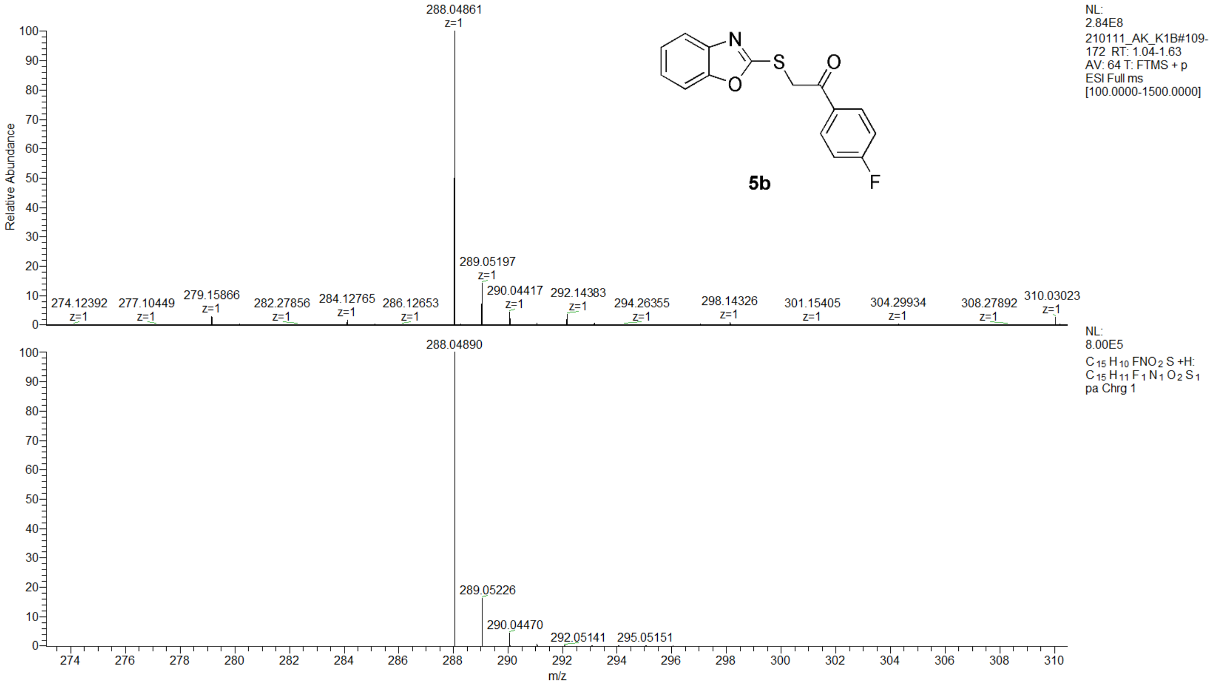

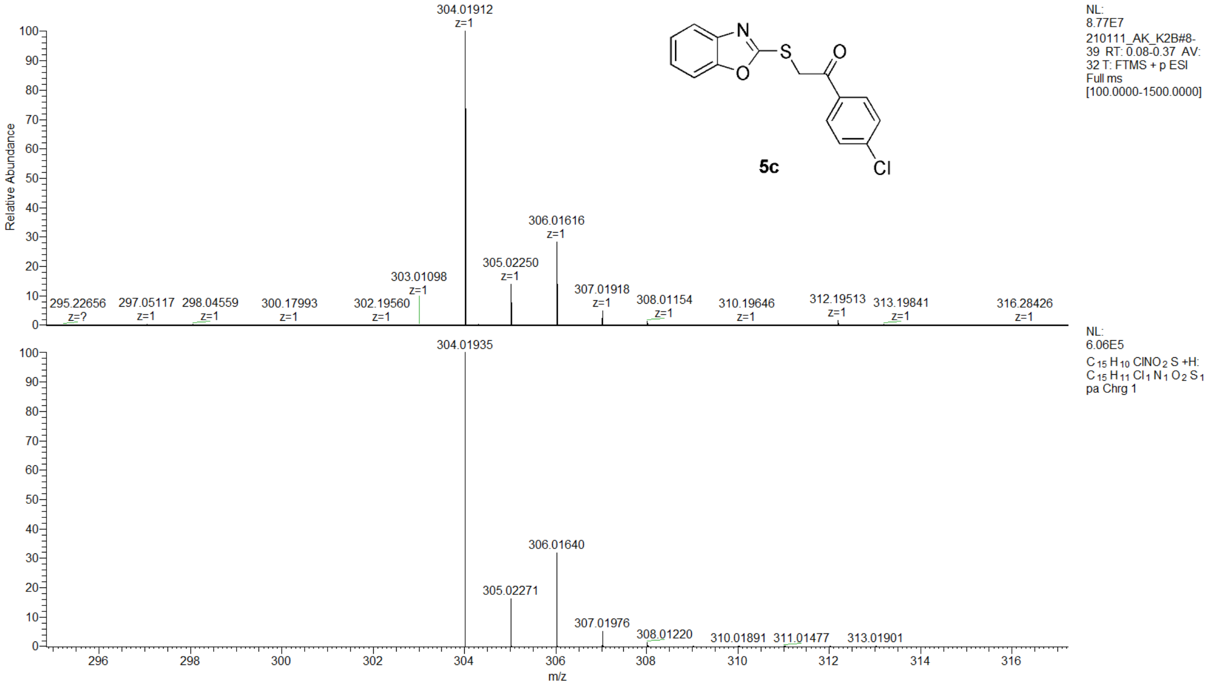

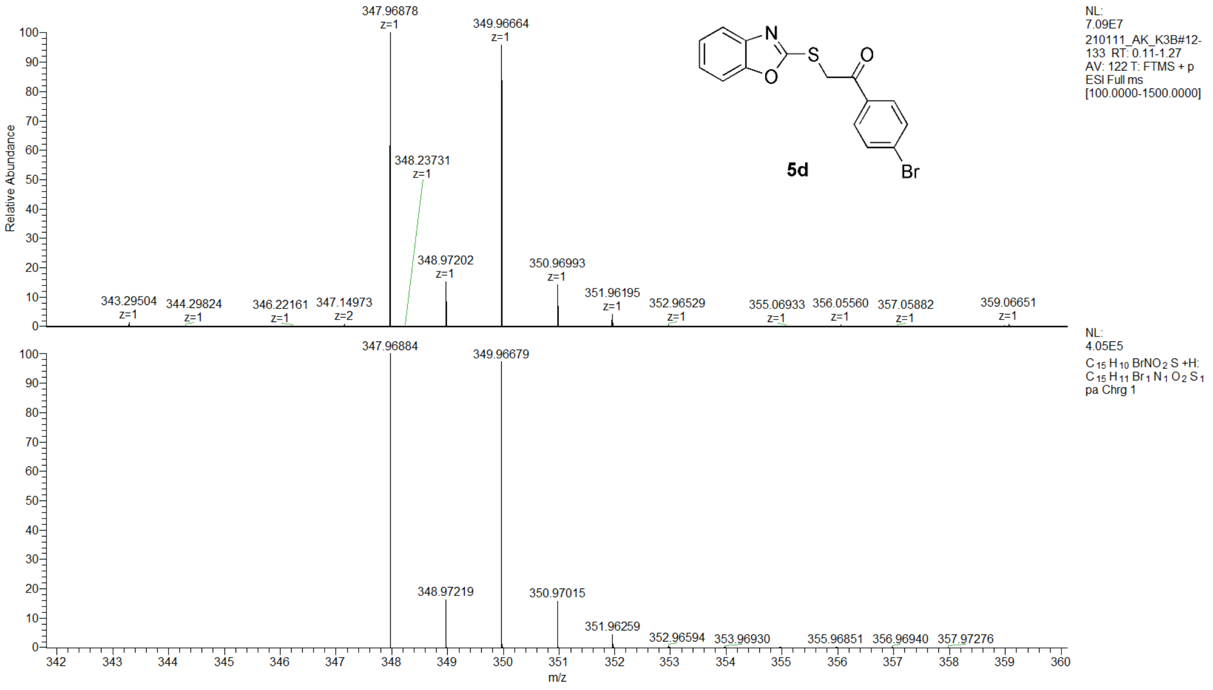
5d
NL:
7.09E7
210111_AK_K3B#12-133 RT: 0.11-1.27 AV: 122 T: FTMS + p ESI Full ms [100.0000-1500.0000]
NL:
4.05E5
C 15 H 10 BrNO 2 S +H:
C 15 H 11 Br 1 N 1 O 2 S 1
pa Chrg 1
Relative Abundance
m/z

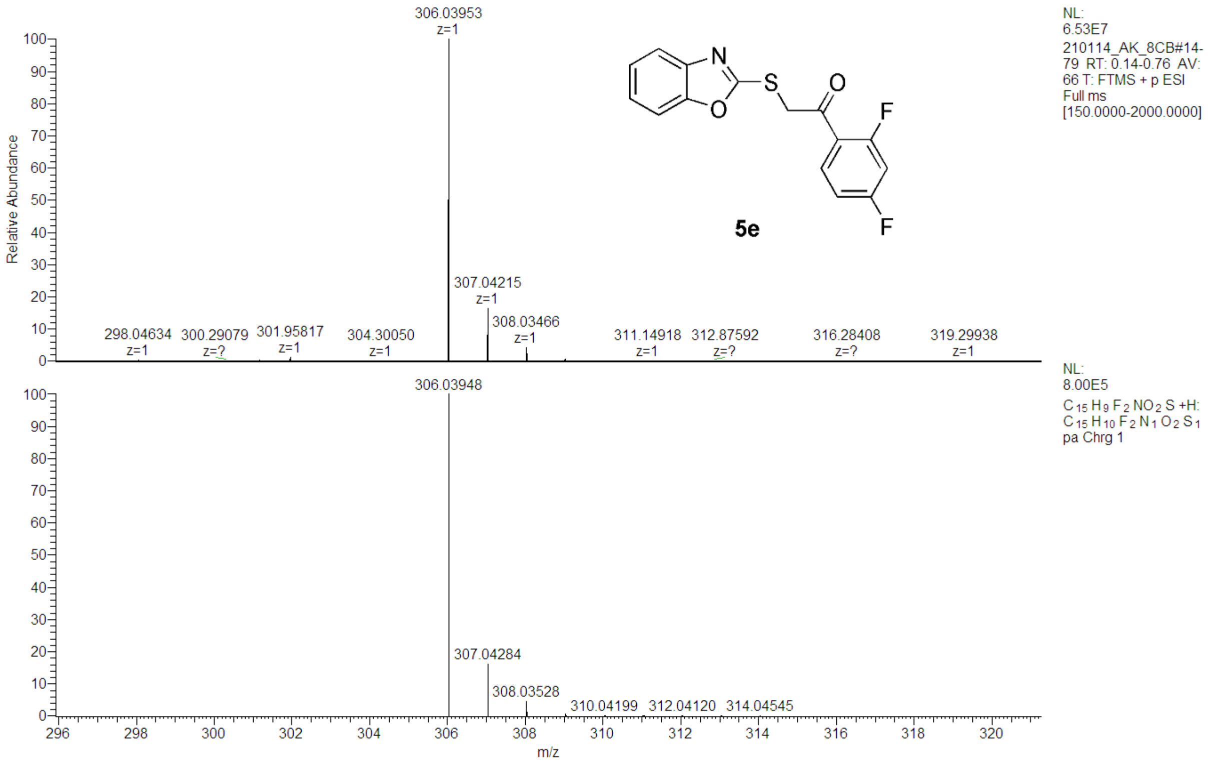
5e
NL:
6.53E7
210114_AK_8CB#14-79 RT: 0.14-0.76 AV: 66 T: FTMS + p ESI Full ms [150.0000-2000.0000]
NL:
8.00E5
C 15 H 9 F 2 NO 2 S +H:
C 15 H 10 F 2 N 1 O 2 S 1
pa Chrg 1
Relative Abundance
m/z

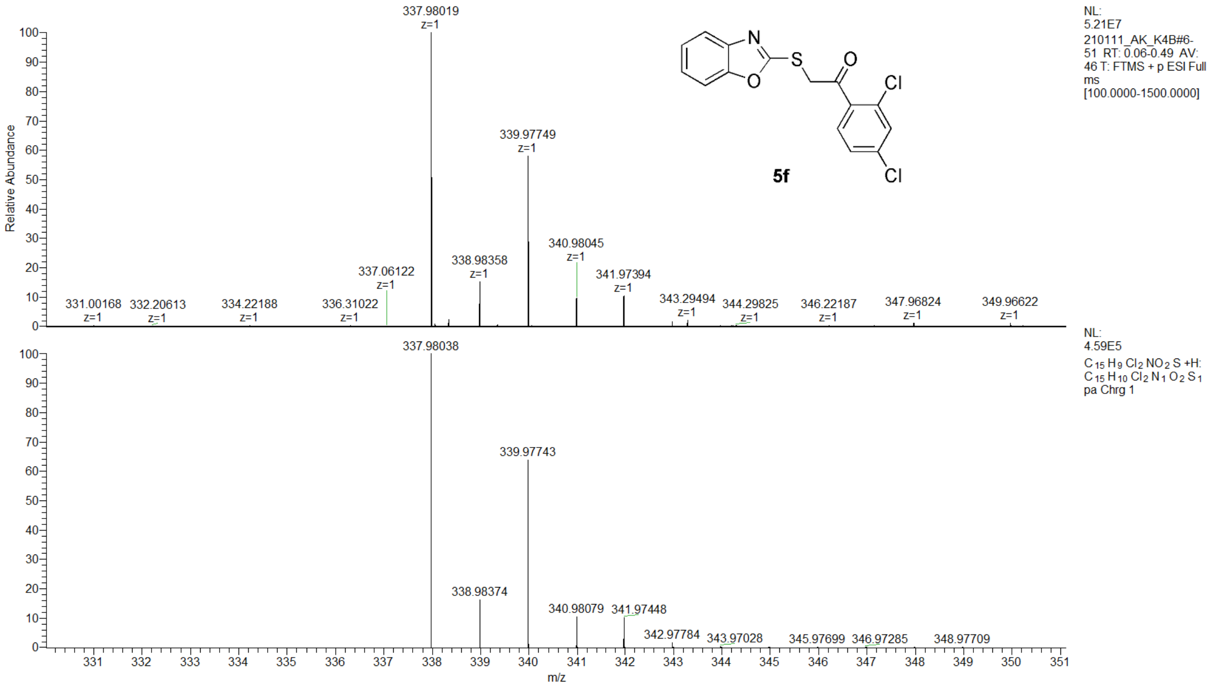

NL: 5.21E7
210111_AK_K4B#6-51 RT: 0.06-0.49 AV: 46 T: FTMS + p ESI Full ms [100.0000-1500.0000]
337.98019 z=1
339.97749 z=1
340.98045 z=1
338.98358 z=1
337.06122 z=1
341.97394 z=1
331.00168 z=1
332.20613 z=1
334.22188 z=1
336.31022 z=1
343.29494 z=1
344.29825 z=1
346.22187 z=1
347.96824 z=1
349.96622 z=1
5f
N
O
S
O
Cl
Cl
Relative Abundance
NL: 4.59E5
C 15 H 9 Cl 2 NO 2 S +H: C 15 H 10 Cl 2 N 1 O 2 S 1 pa Chrg 1
337.98038
339.97743
338.98374
340.98079
341.97448
342.97784
343.97028
345.97699
346.97285
348.97709
m/z

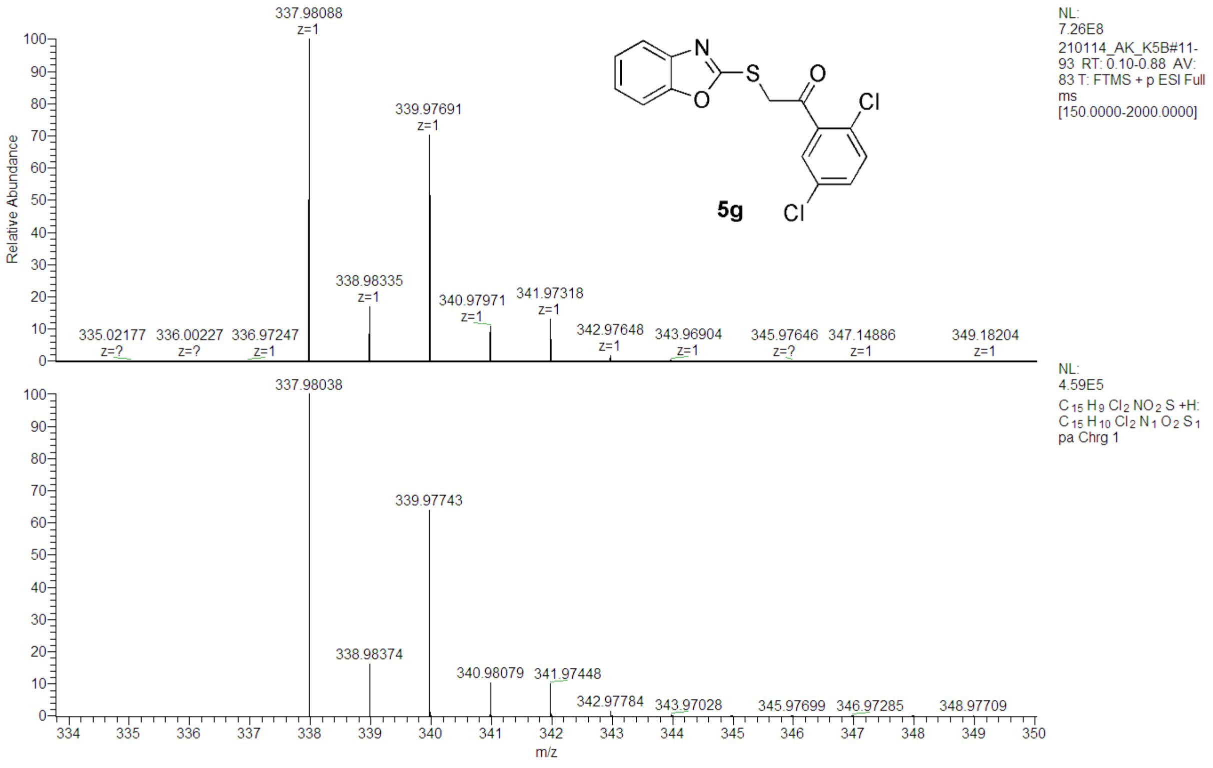

NL: 7.26E8
210114_AK_K5B#11-93 RT: 0.10-0.88 AV: 83 T: FTMS + p ESI Full ms [150.0000-2000.0000]
337.98088 z=1
339.97691 z=1
338.98335 z=1
340.97971 z=1
341.97318 z=1
342.97648 z=1
335.02177 z=?
336.00227 z=?
336.97247 z=1
343.96904 z=1
345.97646 z=?
347.14886 z=1
349.18204 z=1
5g
N
O
S
O
Cl
Cl
Relative Abundance
NL: 4.59E5
C 15 H 9 Cl 2 NO 2 S +H: C 15 H 10 Cl 2 N 1 O 2 S 1 pa Chrg 1
337.98038
339.97743
338.98374
340.98079
341.97448
342.97784
343.97028
345.97699
346.97285
348.97709
m/z

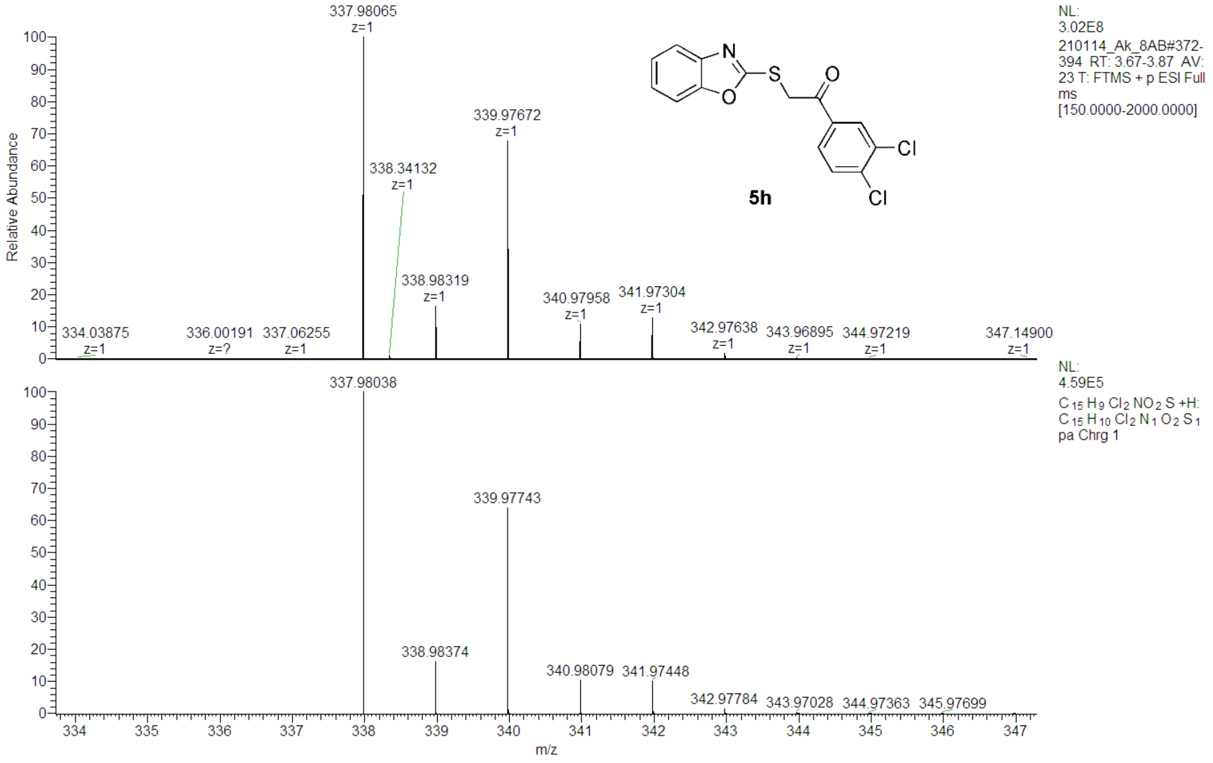

5h
NL: 3.02E8
210114_Ak_8AB#372-394 RT: 3.67-3.87 AV: 23 T: FTMS + p ESI Full ms [150.0000-2000.0000]
NL: 4.59E5
C 15 H 9 Cl 2 NO 2 S +H: C 15 H 10 Cl 2 N 1 O 2 S 1 pa Chrg 1
Relative Abundance
m/z

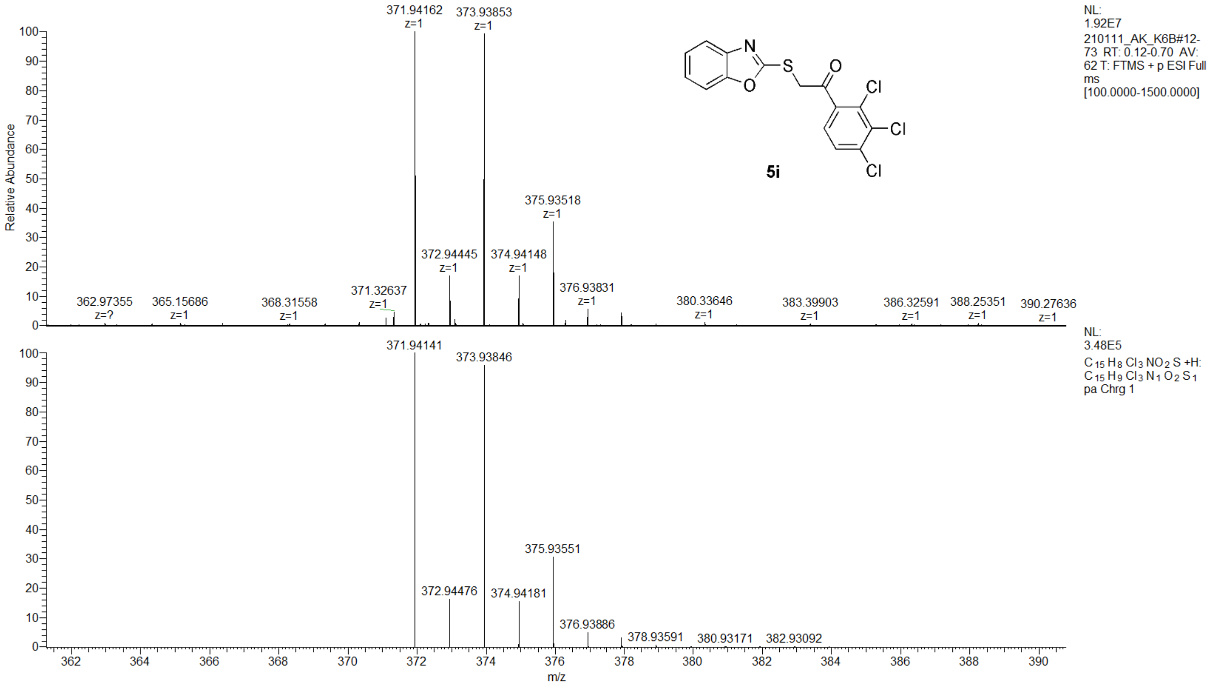

5i
NL: 1.92E7
210111_AK_K6B#12-73 RT: 0.12-0.70 AV: 62 T: FTMS + p ESI Full ms [100.0000-1500.0000]
NL: 3.48E5
C 15 H 8 Cl 3 NO 2 S +H: C 15 H 9 Cl 3 N 1 O 2 S 1 pa Chrg 1
Relative Abundance
m/z

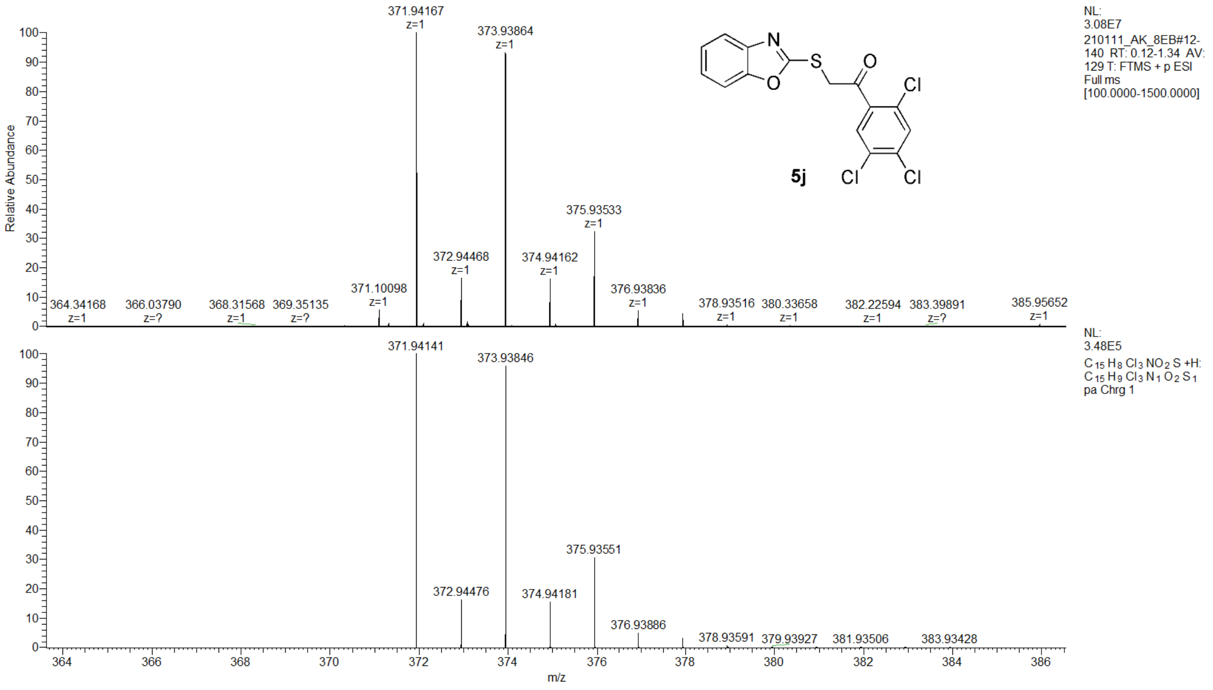
5j
NL: 3.08E7
210111_AK_8EB#12-140 RT: 0.12-1.34 AV: 129 T: FTMS + p ESI Full ms [100.0000-1500.0000]
NL: 3.48E5
C 15 H 8 Cl 3 NO 2 S +H: C 15 H 9 Cl 3 N 1 O 2 S 1 pa Chrg 1
Relative Abundance
m/z
371.94167 z=1
373.93864 z=1
375.93533 z=1
372.94468 z=1
374.94162 z=1
371.10098 z=1
376.93836 z=1
364.34168 z=1
366.03790 z=?
368.31568 z=1
369.35135 z=?
378.93516 z=1
380.33658 z=1
382.22594 z=1
383.39891 z=?
385.95652 z=1
371.94141
373.93846
375.93551
372.94476
374.94181
376.93886
378.93591
379.93927
381.93506
383.93428

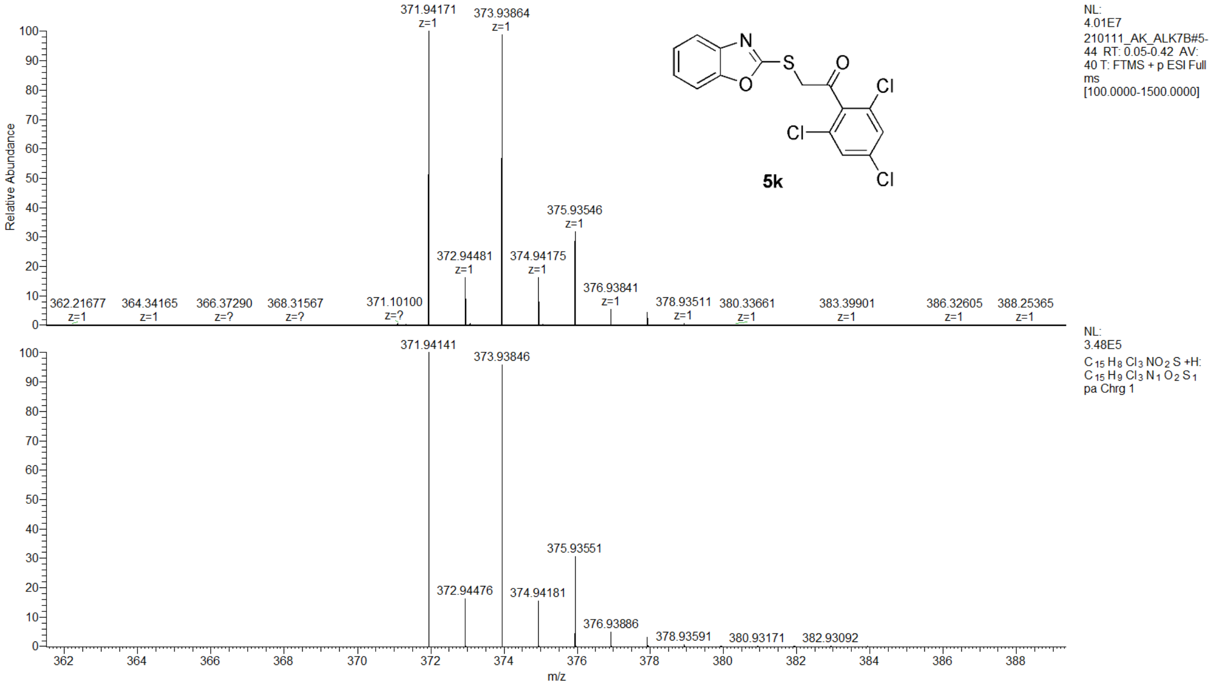
5k
NL: 4.01E7
210111_AK_ALK7B#5-44 RT: 0.05-0.42 AV: 40 T: FTMS + p ESI Full ms [100.0000-1500.0000]
NL: 3.48E5
C 15 H 8 Cl 3 NO 2 S +H: C 15 H 9 Cl 3 N 1 O 2 S 1 pa Chrg 1
Relative Abundance
m/z
371.94171 z=1
373.93864 z=1
375.93546 z=1
372.94481 z=1
374.94175 z=1
376.93841 z=1
362.21677 z=1
364.34165 z=1
366.37290 z=?
368.31567 z=?
371.10100 z=?
378.93511 z=1
380.33661 z=1
383.39901 z=1
386.32605 z=1
388.25365 z=1
371.94141
373.93846
375.93551
372.94476
374.94181
376.93886
378.93591
380.93171
382.93092

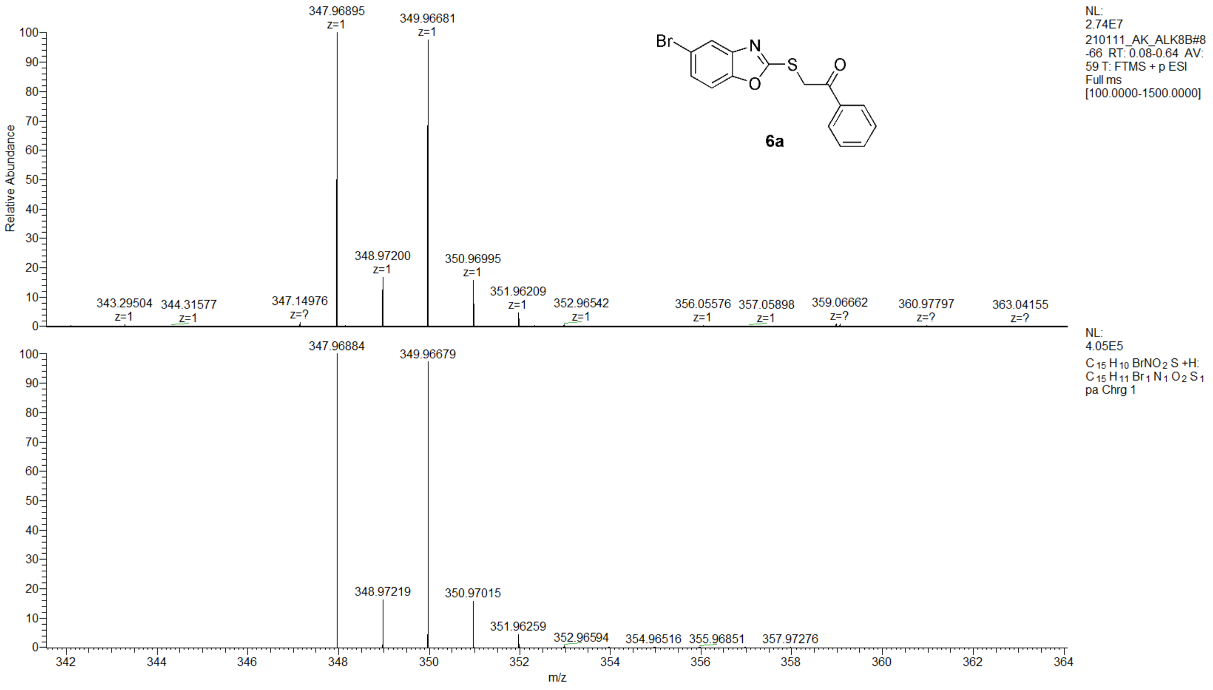
6a
Br
N
O
S
O
NL:
2.74E7
210111_AK_ALK8B#8
-66 RT: 0.08-0.64 AV:
59 T: FTMS + p ESI
Full ms
[100.0000-1500.0000]
NL:
4.05E5
C 15 H 10 BrNO 2 S +H:
C 15 H 11 Br 1 N 1 O 2 S 1
pa Chrg 1
Relative Abundance
m/z
347.96895
349.96681
348.97200
350.96995
351.96209
352.96542
343.29504
344.31577
347.14976
356.05576
357.05898
359.06662
360.97797
363.04155
347.96884
349.96679
348.97219
350.97015
351.96259
352.96594
354.96516
355.96851
357.97276

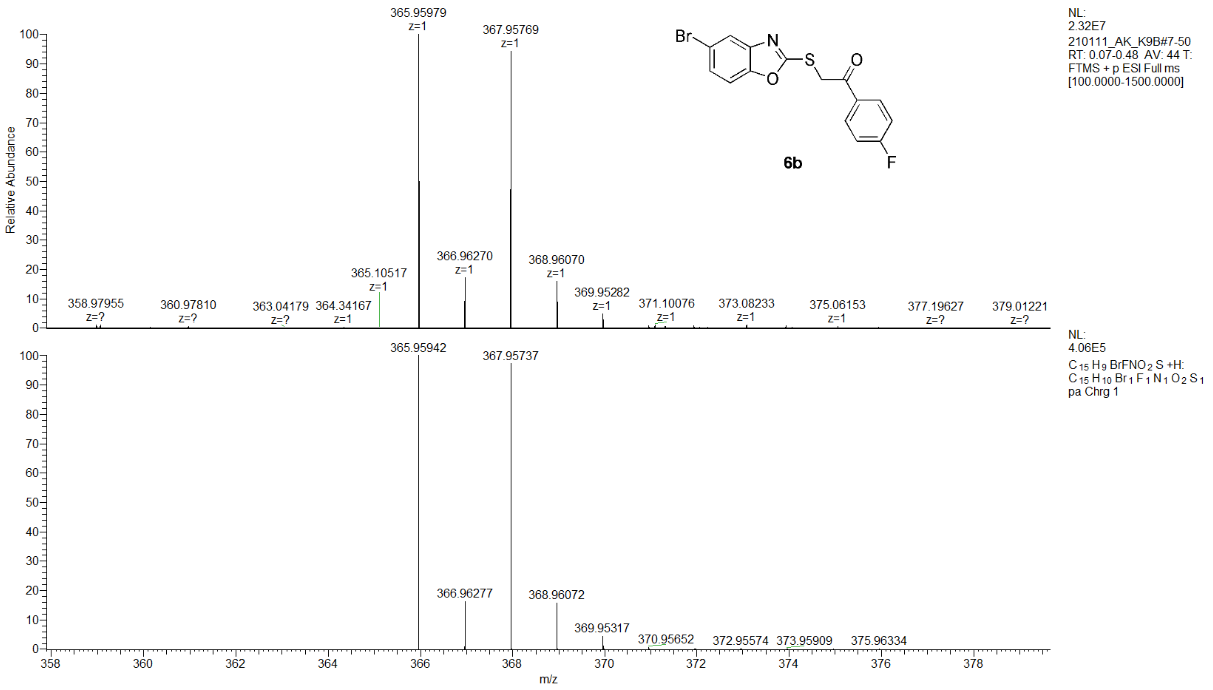
6b
Br
N
O
S
O
F
NL:
2.32E7
210111_AK_K9B#7-50
RT: 0.07-0.48 AV: 44 T:
FTMS + p ESI Full ms
[100.0000-1500.0000]
NL:
4.06E5
C 15 H 9 BrFNO 2 S +H:
C 15 H 10 Br 1 F 1 N 1 O 2 S 1
pa Chrg 1
Relative Abundance
m/z
365.95979
367.95769
365.10517
366.96270
368.96070
369.95282
358.97955
360.97810
363.04179
364.34167
371.10076
373.08233
375.06153
377.19627
379.01221
365.95942
367.95737
366.96277
368.96072
369.95317
370.95652
372.95574
373.95909
375.96334

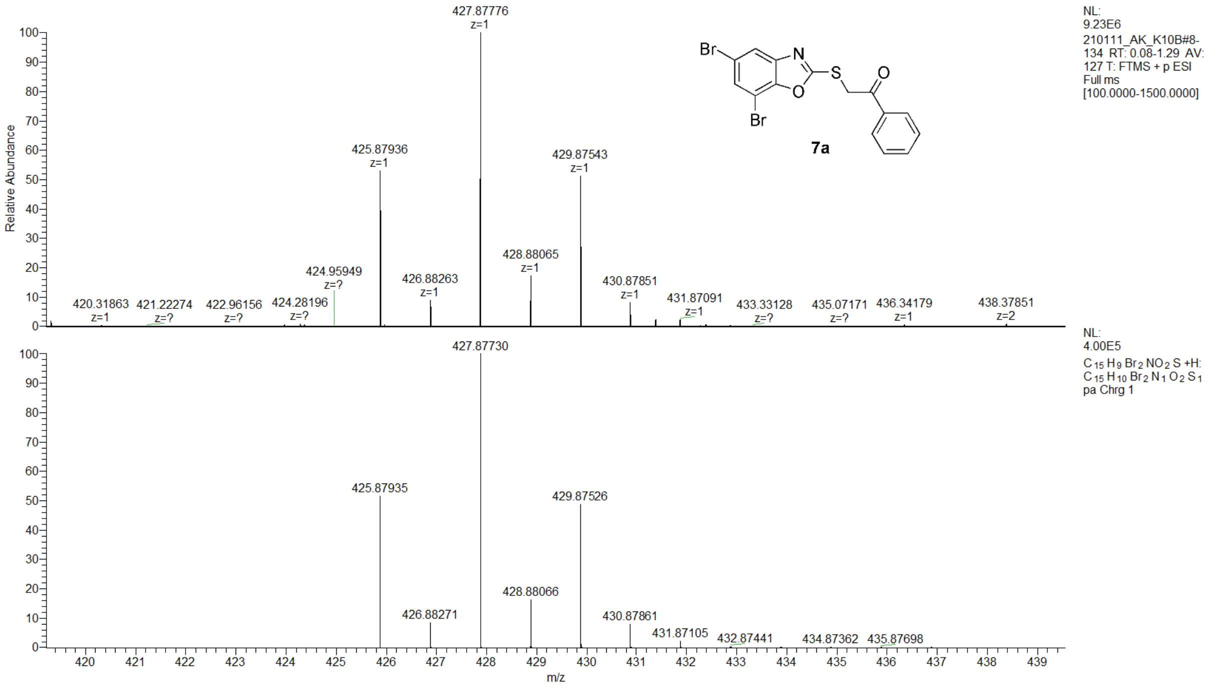

7a
NL: 9.23E6 210111_AK_K10B#8-134 RT: 0.08-1.29 AV: 127 T: FTMS + p ESI Full ms [100.0000-1500.0000]
NL: 4.00E5 C 15 H 9 Br 2 NO 2 S +H: C 15 H 10 Br 2 N 1 O 2 S 1 pa Chrg 1
Relative Abundance
m/z
427.87776 z=1
425.87936 z=1
429.87543 z=1
428.88065 z=1
426.88263 z=1
424.95949 z=?
430.87851 z=1
431.87091 z=1
420.31863 z=1
421.22274 z=?
422.96156 z=?
424.28196 z=?
433.33128 z=?
435.07171 z=?
436.34179 z=1
438.37851 z=2
427.87730
425.87935
429.87526
428.88066
426.88271
430.87861
431.87105
432.87441
434.87362
435.87698

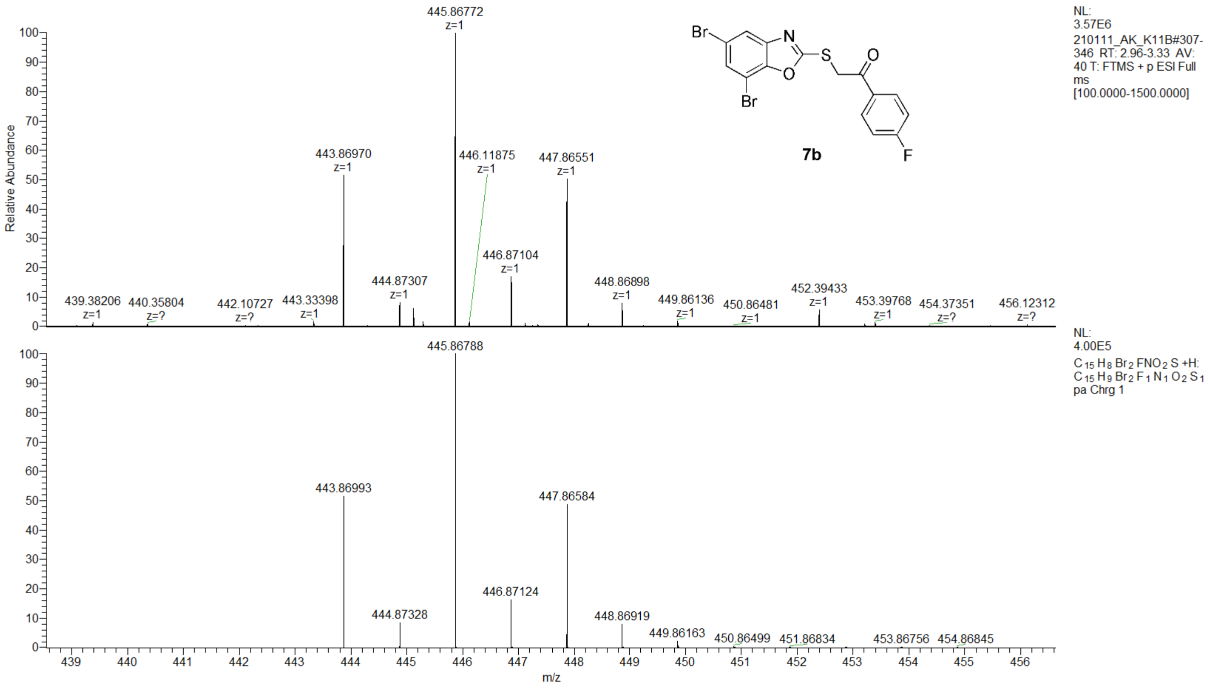

7b
NL: 3.57E6 210111_AK_K11B#307-346 RT: 2.96-3.33 AV: 40 T: FTMS + p ESI Full ms [100.0000-1500.0000]
NL: 4.00E5 C 15 H 8 Br 2 FNO 2 S +H: C 15 H 9 Br 2 F 1 N 1 O 2 S 1 pa Chrg 1
Relative Abundance
m/z
445.86772 z=1
443.86970 z=1
446.11875 z=1
447.86551 z=1
446.87104 z=1
444.87307 z=1
448.86898 z=1
452.39433 z=1
439.38206 z=1
440.35804 z=?
442.10727 z=?
443.33398 z=1
449.86136 z=1
450.86481 z=1
453.39768 z=1
454.37351 z=?
456.12312 z=?
445.86788
443.86993
447.86584
446.87124
444.87328
448.86919
449.86163
450.86499
451.86834
453.86756
454.86845

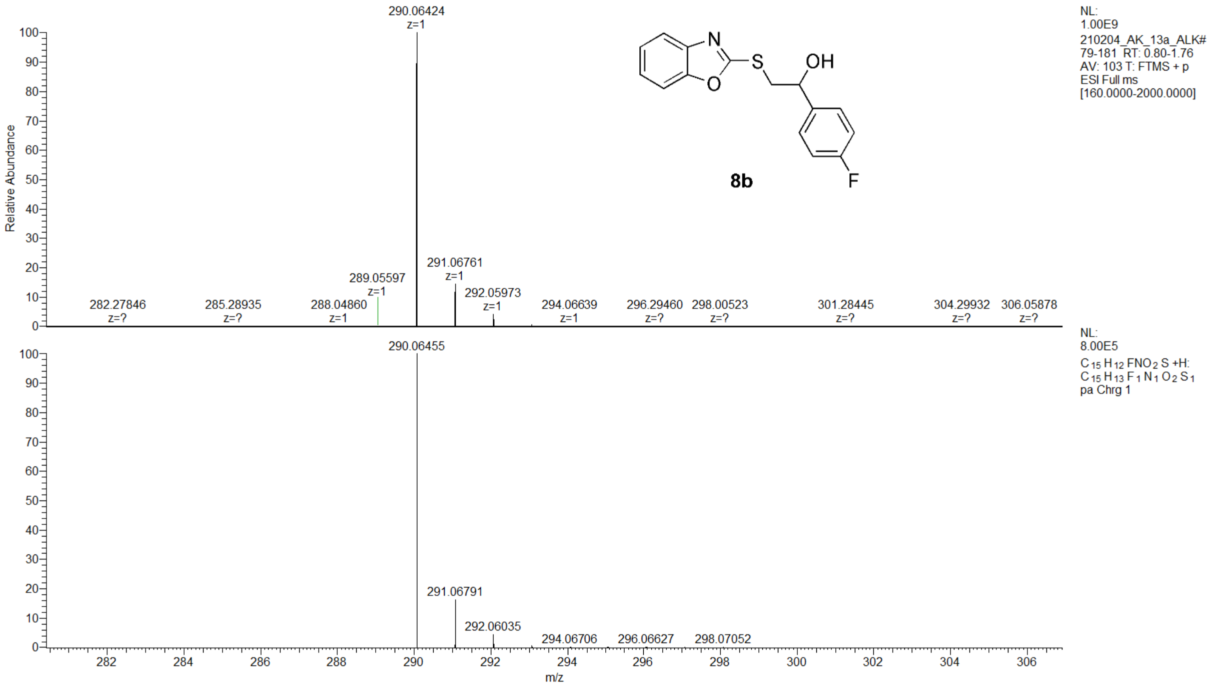
290.06424
z=1
8b
NL: 1.00E9
210204_AK_13a_ALK#79-181 RT: 0.80-1.76 AV: 103 T: FTMS + p ESI Full ms [160.0000-2000.0000]
291.06761
289.05597
292.05973
282.27846
285.28935
288.04860
294.06639
296.29460
298.00523
301.28445
304.29932
306.05878
NL: 8.00E5
C 15 H 12 FNO 2 S +H: C 15 H 13 F 1 N 1 O 2 S 1 pa Chrg 1
290.06455
291.06791
292.06035
294.06706
296.06627
298.07052
Relative Abundance
m/z

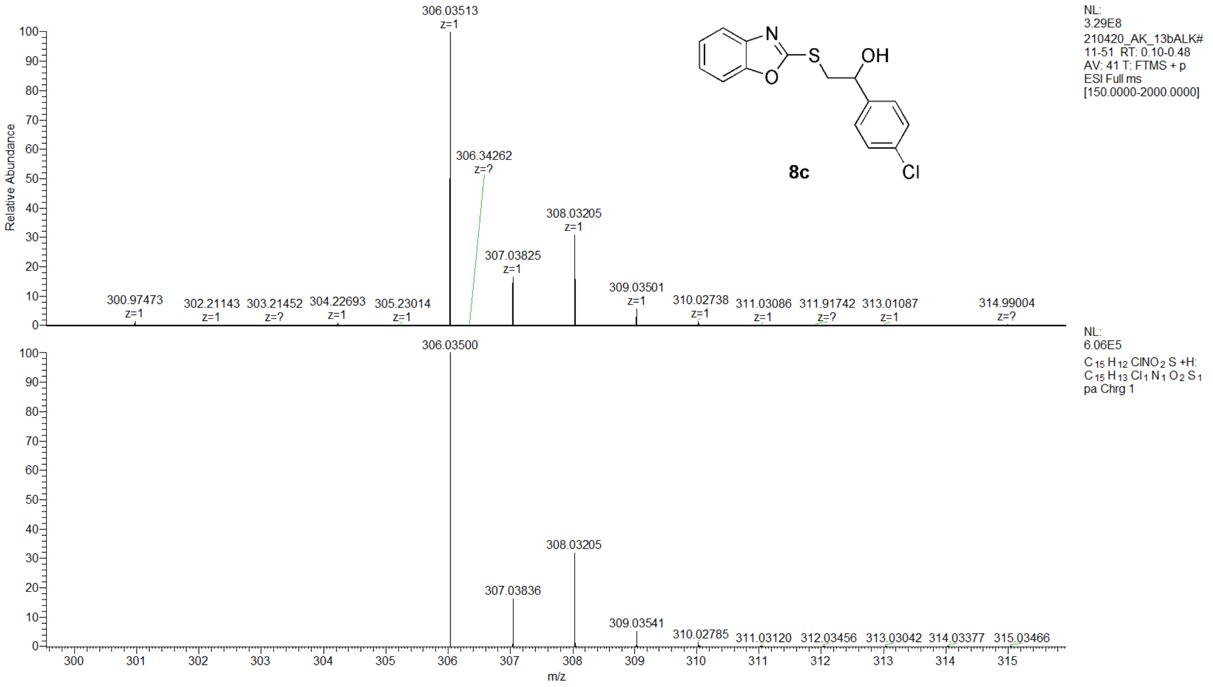
306.03513
z=1
8c
NL: 3.29E8
210420_AK_13bALK#11-51 RT: 0.10-0.48 AV: 41 T: FTMS + p ESI Full ms [150.0000-2000.0000]
306.34262
308.03205
307.03825
309.03501
300.97473
302.21143
303.21452
304.22693
305.23014
310.02738
311.03086
311.91742
313.01087
314.99004
NL: 6.06E5
C 15 H 12 ClNO 2 S +H: C 15 H 13 Cl 1 N 1 O 2 S 1 pa Chrg 1
306.03500
308.03205
307.03836
309.03541
310.02785
311.03120
312.03456
313.03042
314.03377
315.03466
Relative Abundance
m/z

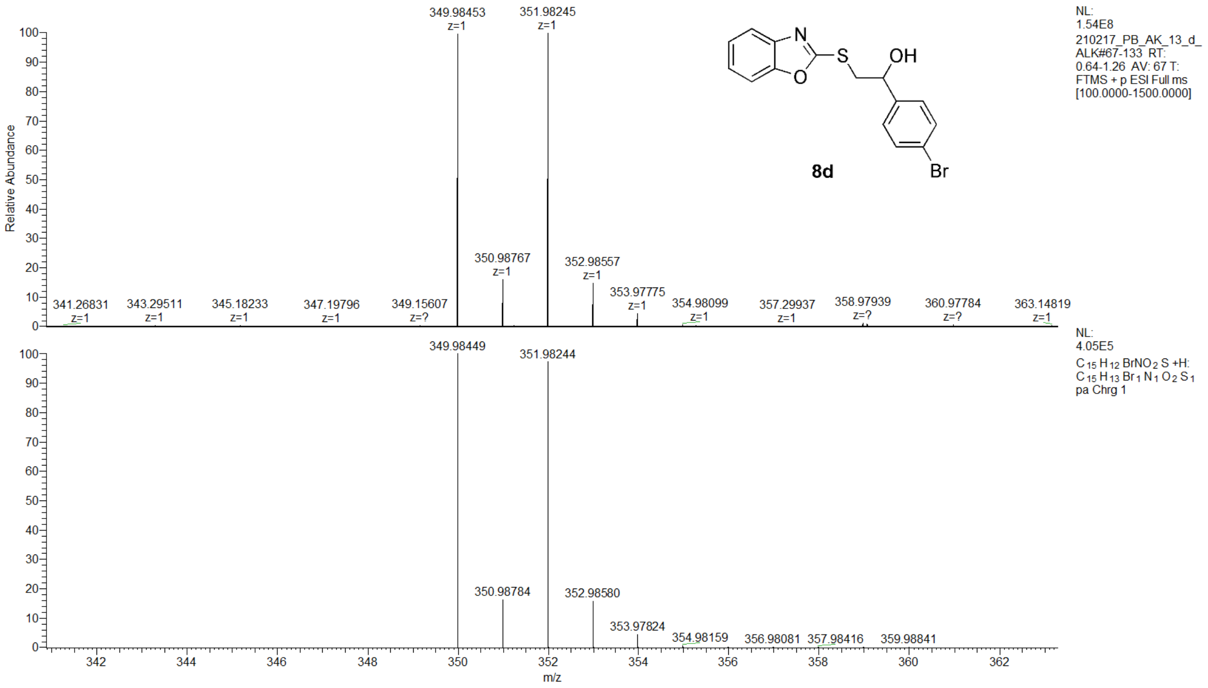
349.98453 z=1
351.98245 z=1
NL: 1.54E8
210217_PB_AK_13_d_ ALK#67-133 RT: 0.64-1.26 AV: 67 T: FTMS + p ESI Full ms [100.0000-1500.0000]
8d
N
O
S
OH
Br
Relative Abundance
350.98767 z=1
352.98557 z=1
353.97775 z=1
341.26831 z=1
343.29511 z=1
345.18233 z=1
347.19796 z=1
349.15607 z=?
354.98099 z=1
357.29937 z=1
358.97939 z=?
360.97784 z=?
363.14819 z=1
NL: 4.05E5
C 15 H 12 BrNO 2 S +H: C 15 H 13 Br 1 N 1 O 2 S 1 pa Chrg 1
349.98449
351.98244
350.98784
352.98580
353.97824
354.98159
356.98081
357.98416
359.98841
m/z

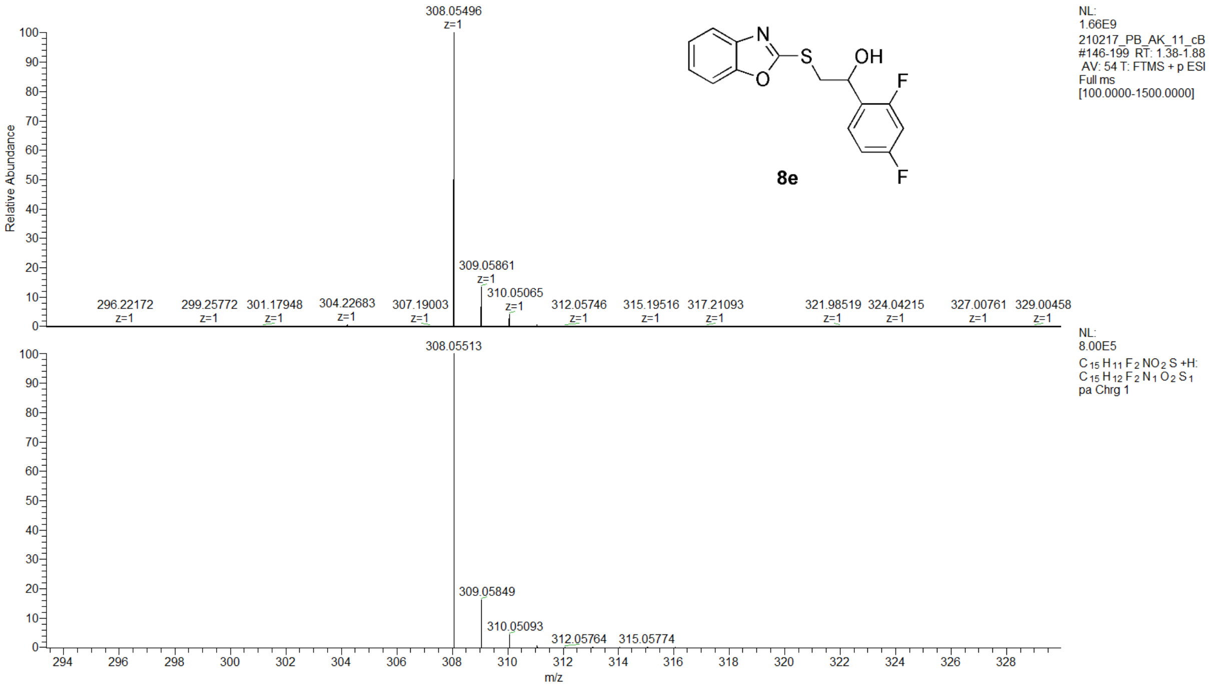
308.05496 z=1
NL: 1.66E9
210217_PB_AK_11_cB #146-199 RT: 1.38-1.88 AV: 54 T: FTMS + p ESI Full ms [100.0000-1500.0000]
8e
N
O
S
OH
F
F
Relative Abundance
309.05861 z=1
310.05065 z=1
296.22172 z=1
299.25772 z=1
301.17948 z=1
304.22683 z=1
307.19003 z=1
312.05746 z=1
315.19516 z=1
317.21093 z=1
321.98519 z=1
324.04215 z=1
327.00761 z=1
329.00458 z=1
NL: 8.00E5
C 15 H 11 F 2 NO 2 S +H: C 15 H 12 F 2 N 1 O 2 S 1 pa Chrg 1
308.05513
309.05849
310.05093
312.05764
315.05774
m/z

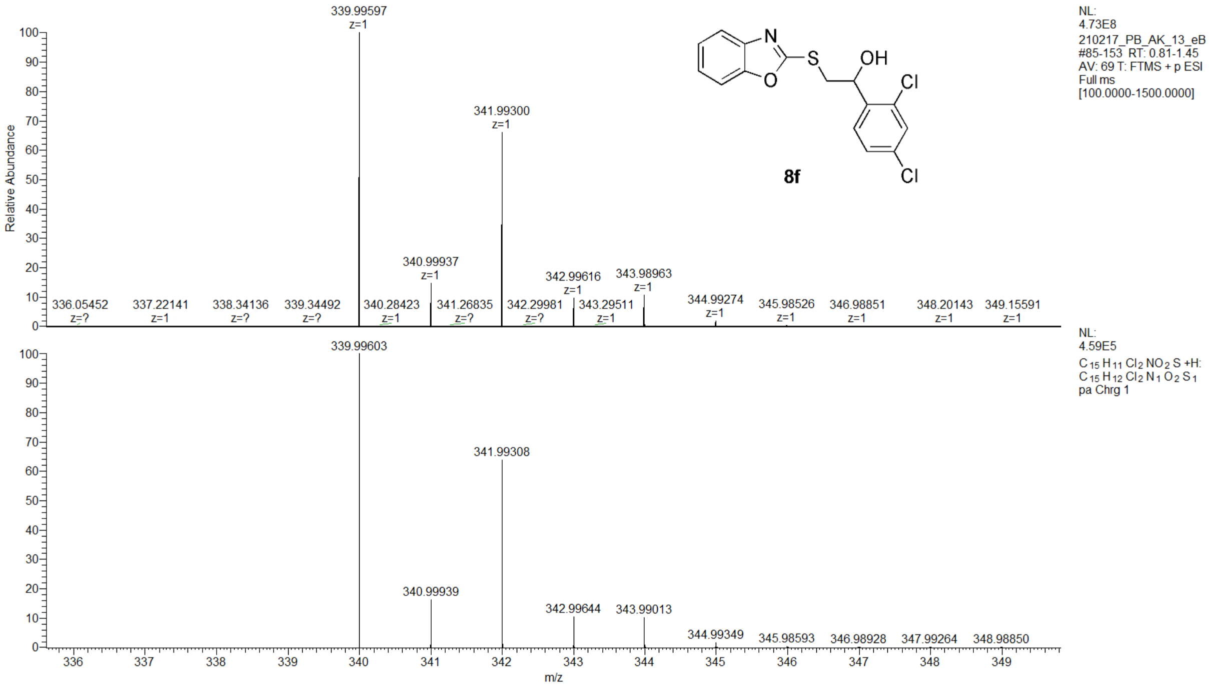

8f
NL: 4.73E8
210217_PB_AK_13_eB #85-153 RT: 0.81-1.45 AV: 69 T: FTMS + p ESI Full ms [100.0000-1500.0000]
NL: 4.59E5
C 15 H 11 Cl 2 NO 2 S +H: C 15 H 12 Cl 2 N 1 O 2 S 1 pa Chrg 1
Relative Abundance
m/z

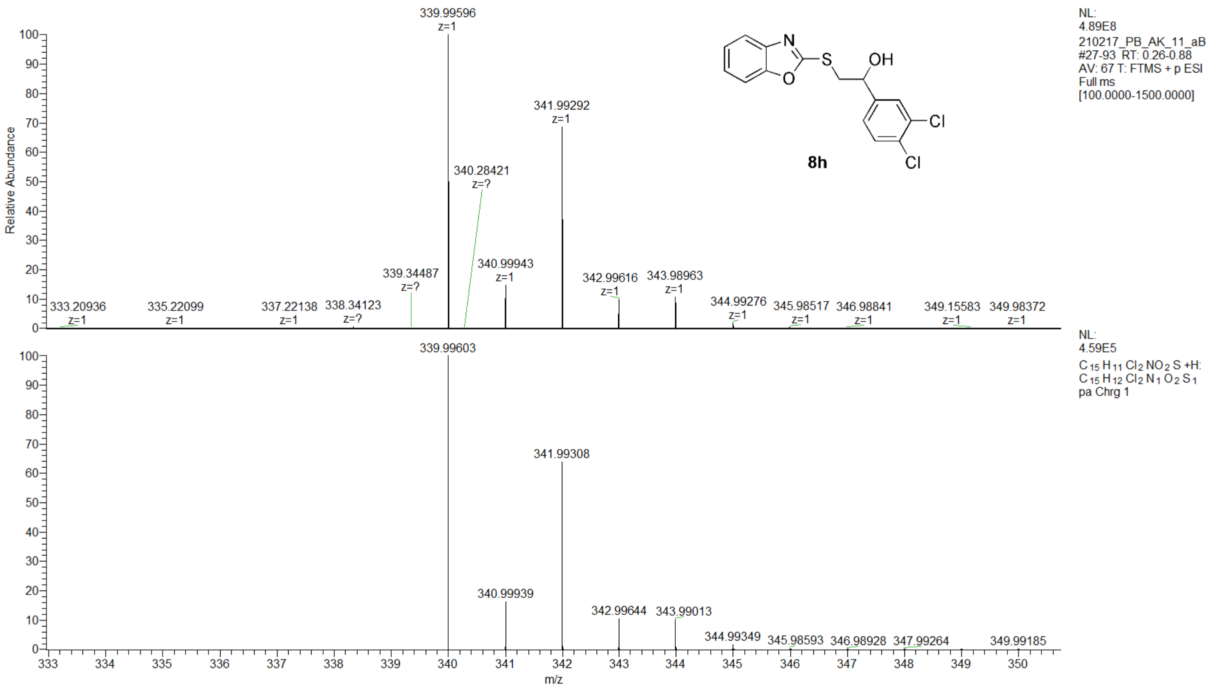

8h
NL: 4.89E8
210217_PB_AK_11_aB #27-93 RT: 0.26-0.88 AV: 67 T: FTMS + p ESI Full ms [100.0000-1500.0000]
NL: 4.59E5
C 15 H 11 Cl 2 NO 2 S +H: C 15 H 12 Cl 2 N 1 O 2 S 1 pa Chrg 1
Relative Abundance
m/z

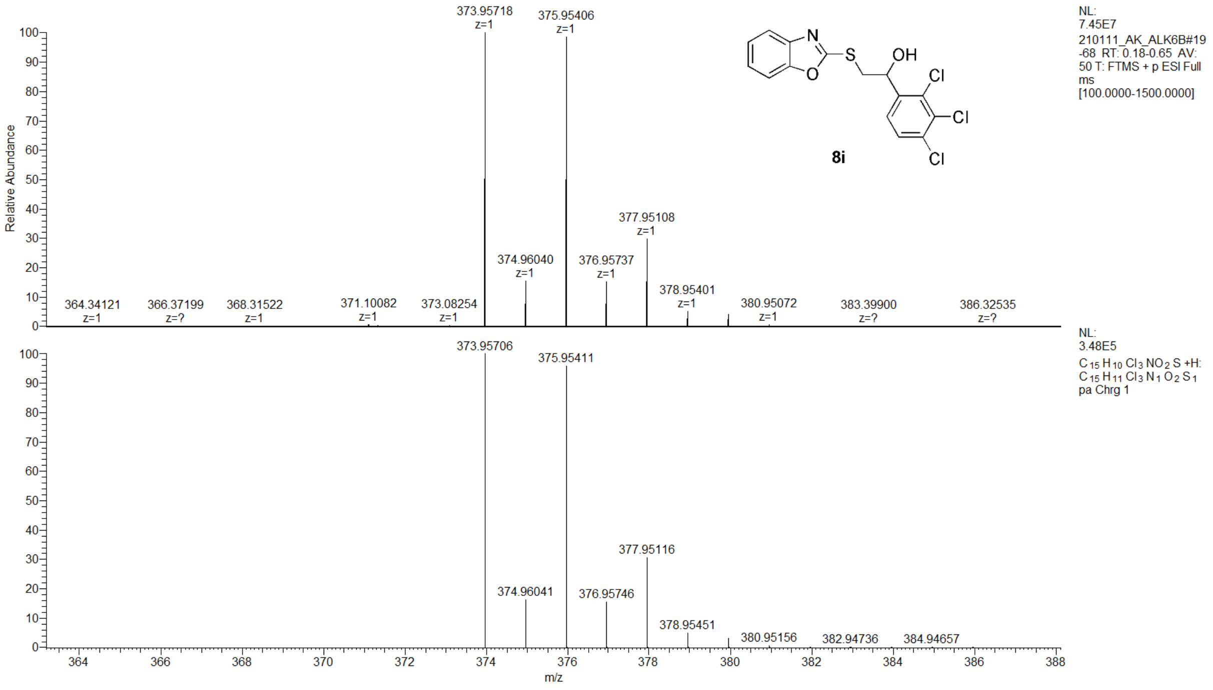
NL:
7.45E7
210111_AK_ALK6B#19
-68 RT: 0.18-0.65 AV:
50 T: FTMS + p ESI Full
ms
[100.0000-1500.0000]
NL:
3.48E5
C 15 H 10 Cl 3 NO 2 S +H:
C 15 H 11 Cl 3 N 1 O 2 S 1
pa Chrg 1
8i
Relative Abundance
m/z

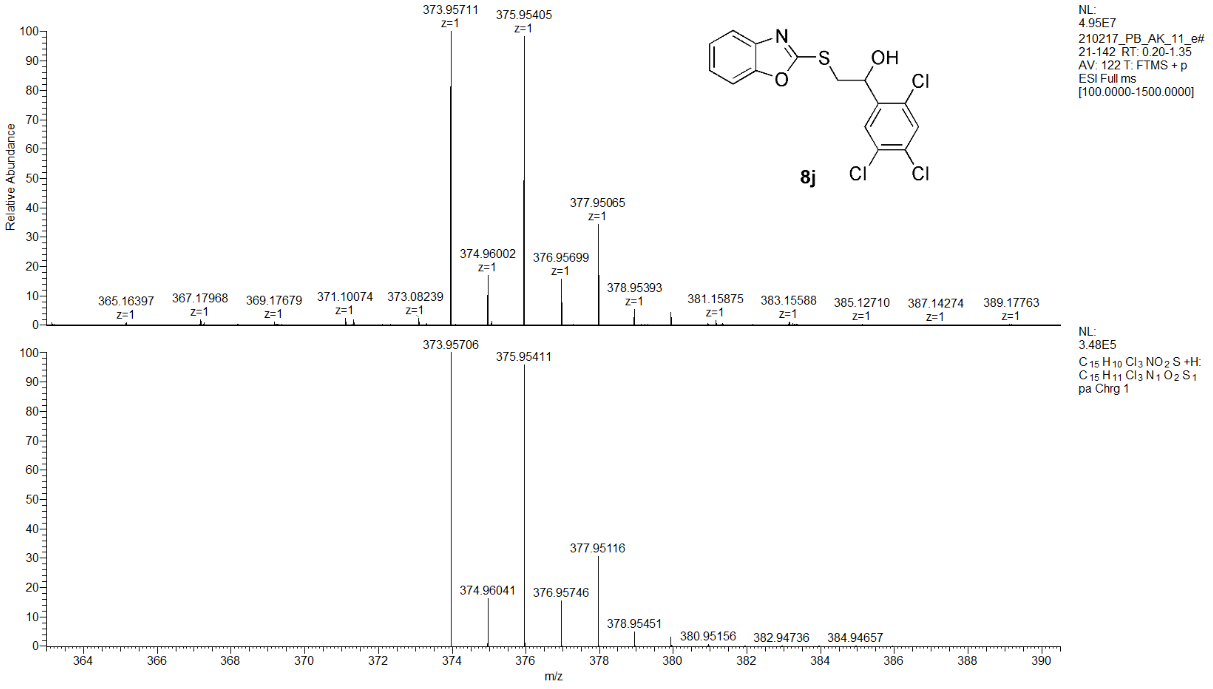
NL:
4.95E7
210217_PB_AK_11_e#
21-142 RT: 0.20-1.35
AV: 122 T: FTMS + p
ESI Full ms
[100.0000-1500.0000]
NL:
3.48E5
C 15 H 10 Cl 3 NO 2 S +H:
C 15 H 11 Cl 3 N 1 O 2 S 1
pa Chrg 1
8j
Relative Abundance
m/z

4. Antifungal assays

Table S1. Antifungal activity of benzoxazole derivatives against *C. albicans* ATCC SC5314.

% cell growth inhibition (mean±SD)							
**Compound no.**	**Incubation time [h]**	**Conc. [μg/mL]**					
		**16**	**8**	**4**	**2**	**1**	**0.5**
**5b**	18	89.9±1.8	84.5±0.9	81.1±5.1	79.8±0.8	73.6±1.0	68.5±13.4
**5c**		94.6±6.7	93.9±3.0	91.3±1.6	78.0±1.8	61.9±19.0	61.8±18.6
**5d**		100.4±4.0**	92.0±0.5	86.9±1.2	83.4±1.6	75.9±12.1	74.8±0.2
**5f***		99.3±3.9*	90.9±1.8	78.4±0.9	77.3±0.9	77.1±2.3	72.7±13.6
**6b**		94.5±1.4	91.2±0.7	90.5±3.5	79.3±4.3	18.6±4.5	15.2±0.4
**7a**		98.7±2.4*	92.8±4.1	88.0±3.7	87.3±5.5	85.7±1.1	49.5±7.8
**7b**		87.5±2.6	82.8±2.0	67.4±6.8	59.4±7.0	18.8±0.3	15.4±2.2

Absorbance at 405 nm, ** total MIC_T, * partial MIC_P

Table S2. Antifungal activity of benzoxazole derivatives against *C. albicans* ATCC SC5314.

Percentage of cells growth inhibition (mean±SD) [%]							
**Compound no.**	**Incubation time [h]**	**Conc [μg/ml]**					
		**16**	**8**	**4**	**2**	**1**	**0.5**
**5b**	18	74.2±1.3	69.4±13.2	81.2±0.7	85.1±1.5	81.9±5.5	91.2±1.5
	48	100.5±4.8	88.8±11.0	71.7±17.1	82.8±13.6	74.5±21.8	84.3±1.2
**5c**	18	61.6±18.4	63.7±18.6	79.9±1.8	95.8±7.1	95.4±2.5	92.1±2.1
	48	48.5±1.4	81.1±7.3	60.7±12.7	98.2±18.5	87.4±27.0	87.3±15.9
**5d**	18	86.5±0.7	75.8±0.6	107.3±3.9	93.1±0.3	83.9±1.4	77.0±11.6
	48	95.7±4.4**	76.2±9.3	88.7±25.6	92.2±16.0	78.6±24.1	73.7±15.3
**5f**	18	78.3±0.9	79.8±0.6	77.9±2.1	91.6±1.6	100.2±4.0	73.5±14.2
	48	88.8±4.6*	83.5±2.0	81.6±24.4	89.6±15.1	94.8±29.3	70.9±20.5
**5g**	18	69.8±0.6	69.4±0.6	71.3±2.7	73.6±0.5	71.9±5.8	74.4±0.2
	48	-	-	-	-	-	-
**5i**	18	75.9±1.6	79.4±2.1	73.6±3.1	67.5±2.5	66.9±16.3	68.4±11.5
	48	-	-	-	-	-	-
**5k**	18	72.3±27.4	74.1±0.9	66.3±10.7	77.0±1.0	75.2±1.7	70.1±14.2
	48	-	-	-	-	-	-
**6a**	18	71.5±7.2	71.6±0.2	75.8±0.9	75.2±2.5	73.9±15.3	79.8±2.8
	48	-	-	-	-	-	-
**6b**	18	73.9±6.0	14.3±0.4	85.8±0.6	12.2±4.8	87.7±5.1	90.1±2.2
	48	29.9±8.0	-	42.4±12.7	6.0±1.7	46.3±11.2	61.7±13.0
**7a**	18	80.5±1.4	86.7±6.7	96.3±3.9*	83.7±8.7	46.1±11.4	81.8±5.5
	48	43.7±7.7	40.1±22.9	79.1±10.3*	26.1±3.8	19.0±4.2	45.5±21.0
**7b**	18	66.5±9.5	17.2±0.7	89.2±4.0	78.8±4.4	21.5±4.6	61.4±9.4
	48	22.9±5.6	18.5±0.2	65.9±20.9	40.7±10.5	-	34.6±14.0

*means partial MIC_P, symbol „-„ means that no inhibition was detected, % of growth inhibition assessed at 460 nm

Table S3. Antifungal activity of benzoxazole derivatives against the *C. albicans* clinical strain.

Percentage of cells growth inhibition (mean±SD) [%]						
Comp.	Conc. [μg/mL]					
	**16**	**8**	**4**	**2**	**1**	**0.5**
**5d**	42.5±9.6	49.8±15.9	14.6±2.5	37.4±17.7	12.5±3.2	17.3±11.1
**5i**	50.4±14.7	3.3±1.9	9.2±3.1	19.1±13.0	39.0±14.3	6.7±2.3
**5k**	62.1±9.9*	35.8±16.4	53.4±20.6	42.0±17.1	-	1.7±0.4
**6a**	85.9±9.9*	28.7±4.7	68.0±7.8	46.2±14.9	3.3±1.8	22.4±10.1

*- partial inhibition MIC_P, % of growth inhibition assessed at 460 nm. Incubation time 48h. Symbol „-„ means that no inhibition was detected

Table S4. Antifungal activity of benzoxazole derivatives against the *C. glabrata* clinical strain.

Percentage of cells growth inhibition (mean±SD) [%]							
**Comp.**	**Incubation time [h]**	**Conc. [μg/mL]**					
		**16**	**8**	**4**	**2**	**1**	**0,5**
**5f**	18	31.3±2.6	45.0±5.1	35.6±3.5	6.8±0.8	46.8±3.9	63.5±3.8
	48	9.3±1.2	10.4±2.8	17.3±5.8	-	28.6±6.9	39.3±4.6
**5i**	18	51.7±1.1	57.1±7.1	14.4±0.9	1.5±0.2	-	7.8±1.8
	48	36.9±4.1	54.5±3.3	16.6±4.1	11.2±2.1	13.1±2.7	14.9±2.0
**5k**	18	7.8±1.1	-	-	-	-	-
	48	37.0±7.3	21.6±2.5	28.3±3.7	28.4±5.0	4.6±0.8	14.2±2.3
**6a**	18	-	-	-	-	-	-
	48	28.5±8.2	8.9±0.9	26.5±4.8	-	12.4±2.1	-

No partial MIC_P was noted, % of growth inhibition assessed at 460 nm. Symbol „-„ means that no inhibition was detected

Table S5. Viability of the Vero cells-treated with benzoxazole derivatives.

Viability of Vero cells (mean±SD) [%]			
**Conc. [μg/mL]**	**Comp.**		
	**5d**	**6a**	**7a**
**256**	38.7±0.6	39.8±2.2	36.2±4.9
**128**	81.6±1.8	82.8±2.0	90.9±6.7
**64**	83.0±4.8	89.9±4.8	85.6±2.9
**32**	100.0±4.9	100.0±0.9	100.0±5.3
**16**	100.0±6.8	97.1±3.8	98.4±6.2
**8**	95.6±5.0	95.0±3.5	95.8±0.2
**4**	97.9±6.8	100.0±1.6	100.0±1.5
**2**	96.6±3.5	100.0±1.0	100.0±2.8
**1**	96.4±6.3	100.0±3.0	100.0±2.2
**0.5**	100.0±6.8	100.0±2.0	99.1±4.9
**0.25**	93.9±4.5	99.9±2.7	98.6±4.9
**0.125**	91.3±2.1	94.6±4.7	94.5±6.3

Table S6. Viability of the MRC-5 cells (mean%±SD) treated with benzoxazole derivatives.

Conc. [µg/mL]	Comp.						
	**5a**	**5e**	**5j**	**8c**	**8d**	**8h**	**8i**
**512**	100.0±16.7	100.0±34.6	53.8±0.0	22.0±0.0	36.7±0.2	18.9±0.0	28.8±0.1
**256**	84.5±14.0	97.4±8.7	49.2±0.0	18.1±0.0	16.5±0.0	18.6±0.0	16.1±0.0
**128**	66.8±3.2	91.6±4.7	61.1±0.1	15.7±0.0	16.0±0.0	18.0±0.0	17.0±0.0
**64**	67.4±3.2	86.0±4.3	71.3±0.1	91.1±0.2	77.9±0.1	93.9±0.0	38.3±0.3
**32**	61.3±3.6	90.0±5.0	60.4±0.0	98.3±0.2	84.5±0.1	100.0±0.1	82.4±0.1
**16**	61.8±5.2	96.6±6.0	59.8±0.1	92.6±0.1	86.8±0.0	98.0±0.0	90.3±0.0
**8**	61.0±4.9	97.5±8.5	64.2±0.1	95.0±0.1	100±0.1	98.1±0.1	88.5±0.1
**4**	63.9±4.7	99.9±3.2	69.2±0.0	99.4±0.2	87.6±0.2	86.8±0.1	84.7±0.1
**2**	63.3±7.1	91.9±6.0	65.5±0.0	85.7±0.1	96.4±0.0	92.2±0.0	88.6±0.0
**1**	63.3±5.2	90.8±5.2	62.3±0.0	80.0±0.1	93.2±0.2	86.7±0.0	85.4±0.0
**0.5**	59.4±4.4	90.4±4.0	55.0±0.1	81.6±0.2	95.9±0.1	90.2±0.0	85.7±0.1
**0.25**	61.1±8.7	87.7±2.9	56.0±0.0	82.1±0.1	92.1±0.1	79.5±0.1	78.6±0.0

Table S7. Ergosterol content measured by HPLC in *C. albicans* ATCC SC5314 treated with benzoxazole derivatives compared to the growth control.

Ergosterol content [%]*		
Conc. [µg/mL]	Comp. no.	
	8c	8f
16	49.2±5.4%	47.7±4.2%

*Compared to the untreated growth control.

Table S8. Fraction of the *C. albicans* ATCC SC5314 cells in accidental cell death (ACD) caused by treatment with benzoxazole derivatives.

Cells (with cell wall)												
Conc. [µg/ml]	5d			6a			7a			Control		
	Necrosis	Apoptosis	Live	Necrosis	Apoptosis	Live	Necrosis	Apoptosis	Live	Necrosis	Apoptosis	Live
4	99.15	0	0.85	99.52	0	0.48	99.35	0.01	0.64	0.33	0	99.77
Protoplasts												
16	99.38	0	0.62	99.33	0	0.67	99.34	0	0.66	0.44	0	99.56
4	99.32	0	0.68	99.53	0.01	0.45	99.41	0	0.59			

Legend: 0 means that no cells were detected

Table S9. Rho123 efflux in cells treated with benzoxazoles in the MIC's and sub-MIC's concentrations.

Conc. [µg/mL]	Comp. no.						
	5a	5e	5j	8c	8d	8h	8i
0.125	-	-	37±5.9	-	-	5±0.8	-10±1.5
1.25	-	-	33±1.6	-	-	23±4.9	-3±0.2
16	18±2.6	14±0.6	-	5±0.8	7±1.5	-	-
160	11±2.6	8±0.7	-	-3±0.4	-6±1.4	-	-

Figure 1S. Interaction between benzoxazoles and ergosterol.

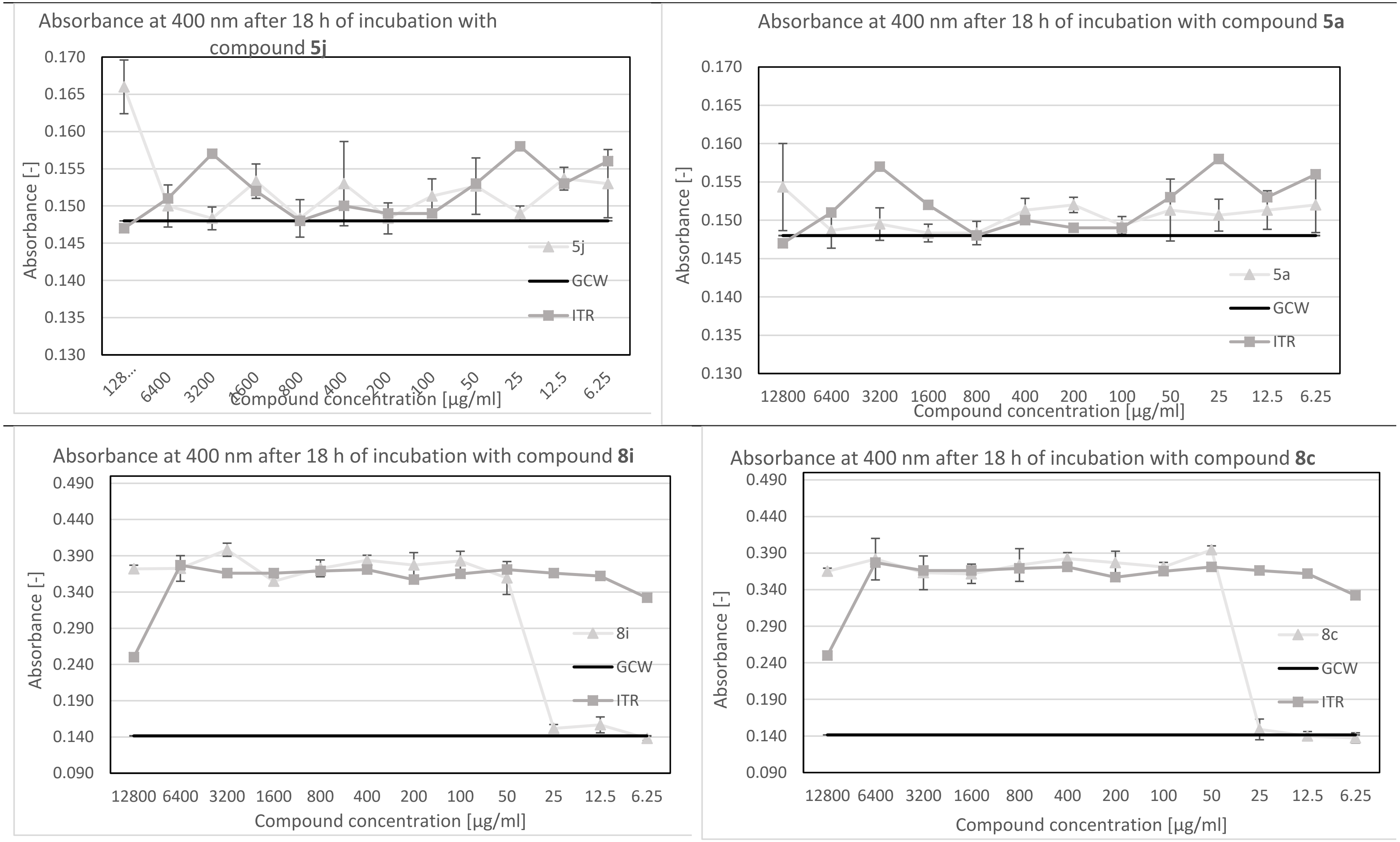

Supplement: Supplementary file 1 [file molecules-26-05008-s001.zip › molecules-1321955-supplementary.pdf]
